# Inflammatory bowel disease and cardiovascular disease: A two-sample Mendelian randomization analysis

**DOI:** 10.3389/fcvm.2022.927120

**Published:** 2022-09-02

**Authors:** Kaiwen Wu, Aoshuang Li, Lei Liu, Tao Shu, Demeng Xia, Xiaobin Sun

**Affiliations:** ^1^Department of Gastroenterology, The Third People’s Hospital of Chengdu, The Affiliated Hospital of Southwest Jiaotong University, Chengdu, Sichuan, China; ^2^Medical Research Center, The Third People’s Hospital of Chengdu, The Affiliated Hospital of Southwest Jiaotong University, Chengdu, Sichuan, China; ^3^Luodian Clinical Drug Research Center, Shanghai Baoshan Luodian Hospital, Shanghai University, Shanghai, China

**Keywords:** cardiovascular diseases, inflammatory bowel disease, Mendelian randomization, genome-wide association study, causation

## Abstract

**Background:**

Although epidemiological studies have shown a positive relationship between inflammatory bowel disease (IBD) and risk of cardiovascular disease (CVD) outcomes, a solid causal relationship has not been established. Thus, a two-sample Mendelian randomization (MR) study was conducted to explore the potential causal effect between IBD and CVD outcomes.

**Methods:**

We performed a two-sample MR analysis to analyze the causal effect of the IBD on CVD outcome by using summary-level genome-wide association studies of European descent. The inverse-variance weighted (IVW) method was used as the main MR analysis, with complementary analyses of MR Egger, maximum likelihood, weighted median, penalized weighted media, simple mode, weighted mode, and MR-PRESSO methods. Multiple sensitivity analyses were used to evaluate the robustness of our results.

**Results:**

All *P*-values were greater than 0.05 in the IVW method, showing no evidence of a causal association between circulating IBD and CVD. Similar results were observed by using other MR methods. No evidence of heterogeneity, pleiotropy, or outlier single-nucleotide polymorphisms was detected. Sensitivity analyses demonstrated the robustness of the results.

**Conclusion:**

The findings of this study provided no evidence to support that IBD has a large effect on risk of CVD outcomes, which is in contrast to many previous observational reports. Further studies are needed to determine the potential mechanism of association identified in observational studies.

## Introduction

Cardiovascular disease (CVD) is a general term for all cardiovascular and brain diseases related to vasculopathy, mainly heart failure, coronary heart disease, arrhythmia, stroke and cerebral infarction (1, 2). Globally, CVDs are the leading cause of death, morbidity and disability worldwide, causing the increase of social and economic burden. Owing to the severe clinical and social consequences of CVD, there is an urgent need for concerted efforts to identify risk factors leading to CVD for early prevention and intervention (3). The occurrence and progression of CVD may be driven by genetic or environmental factors. Besides, some epidemiological studies have shown a strong association between inflammatory disease and CVD outcomes (4).

Inflammatory bowel disease (IBD), including ulcerative colitis and Crohn’s disease, is an immune-mediated chronic inflammatory disease of the gastrointestinal tract, characterized by abdominal pain and diarrhea (5). IBD is often accompanied by systemic inflammation and other extraintestinal manifestations. More than 6.8 million patients are suffering from IBD worldwide (6).

Several studies have proved that IBD patients have higher rates of CVD outcomes. The high levels of C-reactive protein, circulating inflammatory cytokines, immunoglobulins G and M, antineutrophil cytoplasmic antibody, vascular endothelial growth factor, and interleukin-1 in patients with IBD can lead to elevated level of oxidative stress, endothelial dysfunction, prothrombotic state, microvascular, and macrovascular dysfunction, which could further promote the formation of atherosclerosis, hence, resulting in an increased risk of CVD outcomes (7–9). A cohort study of 28,833 IBD patients from Denmark with a 13-year follow-up showed that IBD is associated with the development of coronary atherosclerotic heart disease (10). Findings from previous systematic reviews and meta-analyses also indicated that patients with IBD may possess a higher risk of CVD outcomes (10–12). However, Sridhar et al. found no increased risk of myocardial ischemia, cardiomyopathy, conduction disorders, and embolic strokes in patients with IBD after adjusting for confounding factors such as hypertension, diabetes, and hyperlipidemia (13). Studies evaluating the association between IBD and risk of CVD outcomes have reported inconsistent results, most likely due to sample size limitations. Furthermore, observational epidemiological studies are susceptible to confounding and reverse causation (14). The causal relationship between IBD and CVD outcomes requires further investigations. Although randomized controlled studies (RCTs) can prevent selection bias and blinded analysis is able to improve the reliability of the results, RCTs are difficult or impractical to perform for the sake of high economic cost, intensive labor, a great deal of resource and time, as well as ethical limitations (15).

Mendelian randomization (MR) is a method of studying genetic epidemiology that utilizes one or multiple genetic variants, such as single nucleotide polymorphisms (SNPs), to assess causal effects between exposures and outcomes (16). Compared with traditional observational epidemiological studies, MR follows the principle of allele separation and free recombination of non-alleles and is not affected by confounding factors (17). Because germline genetic variants are fixed at conception, MR studies are unaffected by the disease process and can avoid confounding and reverse causality, further mitigating the influence of confounding factors (18). Besides, the high measurement precision of genetic variation can prevent regression dilution bias caused by measurement error (19). In this study, a two-sample MR study was performed to explore the genetic association between IBD and CVD outcomes using large-scale publicly available genome-wide association study (GWAS) data.

## Materials and methods

### Data resources

**FIGURE 1:**
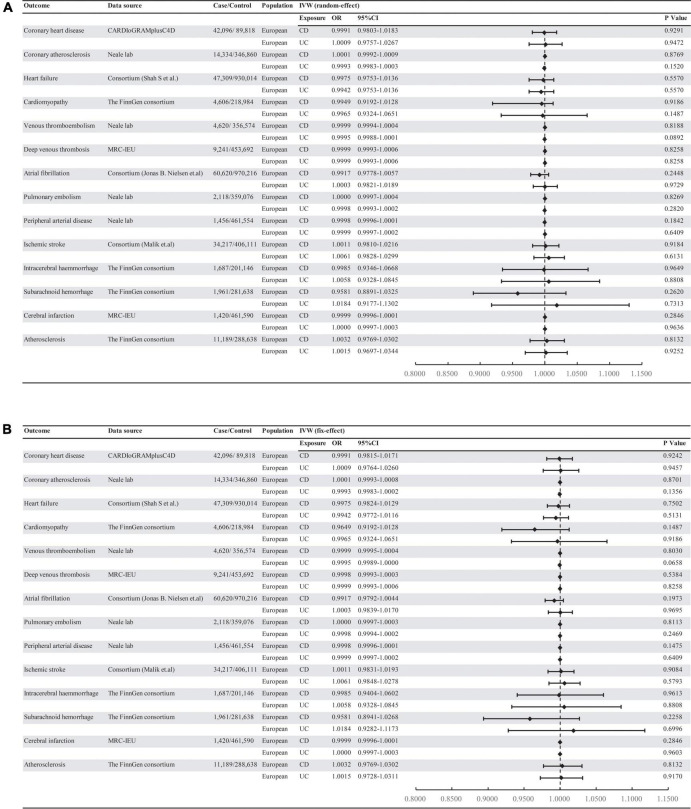
Associations of genetically predicted IBD with risk of 14 CVD outcomes. **(A)** Random-effect. **(B)** Fix-effect. UC, ulcerative colitis; CD, Crohn’s disease; MRC-IEU: MRC Integrative Epidemiology Unit.

Inflammatory bowel disease GWAS summary statistics were obtained from the International Inflammatory Bowel Disease Genetics Consortium (IIBDGC),^1^ which included a total sample size of 65,642 participants of predominantly European ancestry (cases/controls for ulcerative colitis: 13,768/33,977 and Crohn’s disease: 17,897/33,977) (20). The large-scale GWAS summary datasets of about 14 CVD outcomes phenotypes were obtained from United Kingdom Biobank, the FinnGen consortium, and large genetic consortia (21–24). Shah et al. (21) conducted a GWAS comprising 47,309 cases and 930,014 controls of European ancestry across 26 studies from the Heart Failure Molecular Epidemiology for Therapeutic Targets Consortium. Malik et al. (24) performed a genome-wide association meta-analysis in 440,328 European individuals (34,217 ischemic stroke patients and 406,111 controls), and discovered 22 new stroke risk loci. These datasets were used for MR analysis. The full data set can be downloaded from https://gwas.mrcieu.ac.uk/ and https://www.finngen.fi/en. The detailed information is provided in Figure 1. MR studies were conducted by using summary statistics from GWAS, which were ethically approved and publicly available on the website. The data are free to download and can be used without restrictions.

**FIGURE 2:**
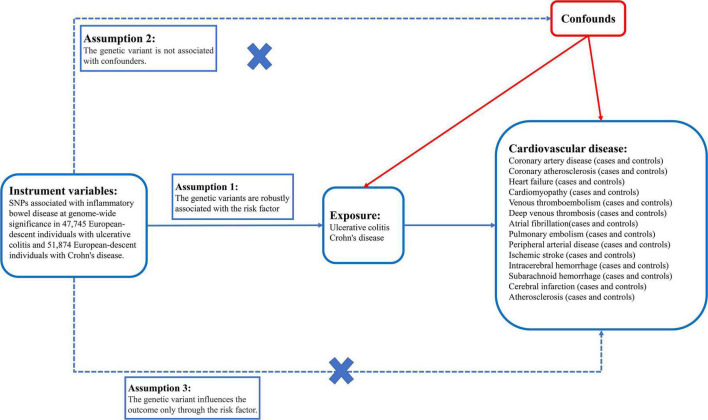
There are three core instrumental variable assumptions for Mendelian randomization analyses.

We selected SNPs that were strongly associated with IBD of genome-wide significance (*P* < 5 × 10^–8^) and pruned SNPs in linkage disequilibrium (*R*^2^ > 0.001 within a 10,000 kb window) with the clump data function in the TwoSampleMR^2^ software package in R (25) (Supplementary material 1). In addition, we used the PhenoScanner^3^ tool to exclude any of the selected SNPs associated with other phenotypes at risk of affecting CVD outcomes. These rigorously selected SNPs were used as the final SNPs for subsequent MR analysis (Figure 2) (26–28). The strength of the genetic instruments was indicated by the *F*-statistic.

### Mendelian randomization

We used two-sample MR, a powerful statistical method, to infer the causality between the two phenotypes. To obtain MR estimate, an inverse-variance weighted (IVW) meta-analysis of each Wald ratio was performed, which is considered the most reliable when there is no evidence of directional pleiotropy (29). The MR-PRESSO method was used to detect outlier variables in IVW analysis by comparing the actual distance of the genetic variants to the expected distance from the regression, assuming the absence of horizontal pleiotropy, and evaluating the causal estimates after removing outliers (30). We also used alternative analyses, including IVW (fixed effects), MR Egger, maximum likelihood, weighted median, penalized weighted media, simple mode, and weighted mode method. Associations were considered statistically significant at a Bonferroni corrected *P*-value below 0.0018 (correcting for 2 exposures and 14 outcomes).

### Pleiotropy and sensitivity analysis

Inverse-variance weighted and MR-Egger regression were used to detect heterogeneity. Cochran’s *Q* test was used to quantify heterogeneities, and *P* < 0.05 was considered statistically significant. Horizontal pleiotropy tests were performed by judging the intercept term in MR-Egger regression. If the intercept term was close to 0 (<0.1) and *P* > 0.05, it indicated that there was no evidence of horizontal pleiotropy in the analysis, showing that the results of MR analyses were reliable. Asymmetry in a funnel plot is also useful for gauging the reliability of a particular MR analyses. Furthermore, sensitivity analysis was performed for qualified CVDs using the leave-one-out method, where the MR was performed again but leaving out each SNP in turn, to determine whether a single SNP was driving the association.

Mendelian randomization analysis were performed using the “TwoSampleMR” and “MR-PRESSO” R package version 0.4.13.^4^ The power of MR was calculated by mRnD.^5^

## Results

### Single nucleotide polymorphisms selected in Mendelian randomization

We obtained 88 SNPs in ulcerative colitis and 122 SNPs in Crohn’s disease, which met the generally accepted genome-wide significance threshold (*P* < 5 × 10^–8^, *r*^2^ < 0.001, kb = 10,000) for exposure (Supplementary material 1). To remove confounding factors, 7 SNPs (rs11229555, rs13430791, rs1801274, rs2516440, rs3024493, rs6062496, and rs9271255) in ulcerative colitis and 11 SNPs (rs17622378, rs2641348, rs3024505, rs3129871, rs3184504, rs516246, rs6062496, rs61839660, rs6679677, rs77981966, and rs780094) in Crohn’s disease were eliminated, which were strongly associated with diabetes, total cholesterol, and smoking. Moreover, estimates of the *F*-statistic indicated no weak instrument bias (all *F*-statistic > 10).

### Association between inflammatory bowel disease and cardiovascular disease risks

Mendelian randomization analysis showed that there was no significant association of IBD with CVD outcomes (all *P* > 0.05) (Figure 1). The result was consistent with IVW (fixed effects), maximum likelihood, weighted medium, penalized weighted media, and MR-PRESSO (Supplementary material 2). The effect size of each SNP on CVDs is shown in Supplementary materials 3, 4.

### Sensitivity analyses

For sensitivity analyses, Cochran’s *Q* test indicated no evidence of heterogeneity (Supplementary material 5). The horizontal pleiotropy between SNPs and outcome was assessed by MR-Egger regression, and the results showed that there was no evidence of horizontal pleiotropy (Supplementary material 5). The funnel plot displayed symmetric pattern of effect size variation around the point estimate, again indicating no apparent horizontal pleiotropy (Supplementary materials 3, 4). The results of leave-one-out sensitivity analyses demonstrated that there was no potentially influential SNP driving the causal link and the stability of our conclusion (Supplementary materials 3, 4).

## Discussion

In this study, a two-sample MR analysis was performed to evaluate the causal effect of IBD on CVD outcomes for the first time. MR analysis did not indicate detrimental effects of genetic increase in IBD on the risk of CVDs. These results were stable in sensitivity analysis by using different Mendelian tools and statistical models, indicating that our analysis is reliable.

Over the past several years, a growing number of observational studies have indicated that IBD is related to an increased risk of CVD outcomes, especially in the active period of IBD, which has been described in studies on large populations (31–33). Meta-analysis showed that there was a positive correlation between IBD and higher incidence of ischemic heart disease and cerebrovascular accidents (11, 34). However, the mechanism of this increased risk remains unclear. Recent fundamental research has suggested that changes in the innate and adaptive immune systems induce an increase in proinflammatory factors and promote the formation of thrombosis and atherosclerosis (35). In addition, changes in gut microbiota composition in patients with IBD may contribute to intestinal epithelial cell apoptosis and barrier dysfunction (36). Both lipopolysaccharide translocation and initiation of an inflammatory cascade result in dysfunction of microvascular and macrovascular endothelial function (37).

Inflammatory bowel disease is less likely to increase the risk of CVD (38). A study that included 31,175 IBD patients (16,779 ulcerative colitis, 10,721 Crohn’s disease, and 3,675 unclassifiable cases) and 154,412 matched controls showed no significant excess of vascular events for IBD patients overall. After adjusting for confounders such as smoking, the risk of stroke, and coronary heart disease was reduced in patients with IBD (39). A meta-analysis also showed that no increased risk of myocardial infarction among patients with ulcerative colitis or Crohn’s disease (39, 40). These results are somewhat inconsistent with previously published observational studies, with a number of possible explanations for this inconsistency. Firstly, the discrepancy between findings from different study designs may be explained by the susceptibility to confound such as socioeconomic, dietary, and lifestyle behavioral factors in traditional observational studies. Hartwig et al. (41) indicated that the risk of coronary artery disease in IBD patients was not increased compared with normal people after excluding traditional risk factors. Thus, we speculated that IBD patients have increased levels of traditional cardiovascular risk factors, resulting in an increased incidence of CVD in observational studies. Recent MR studies showed a lack of genetic causality between IBD and atrial fibrillation (42), which was consistent with our results. Although pleiotropy may cause the bias of the results in MR analysis, there is little possibility in our study (43). Because our MR analysis had more than 80% power to detect the effect of CVDs development (Supplementary material 5). Nonetheless, a very small effect of IBD on CVD outcomes could not be excluded because of tight confidence intervals.

### Strengths and limitations

The main strength of this study is that we used a two-sample MR analysis to assess independent effects of the IBD on multiple CVD outcomes, offering the possibility to overcome several limitations in conventional epidemiological studies. As alleles are randomly assorted and fixed at conception, bias caused by reverse causation can be largely avoided (44). An additional strong point is the large scale of the samples size that enabled us to conduct well-powered MR analyses. Finally, our results were unlikely to be impacted by population stratification bias for the sake of GWAS, which comprised individuals who were primarily of European ancestry (45). In addition, we used the STROBE-MR checklist of recommended items to address in reports of MR studies to check our study (Supplementary material 6).

Inevitably, this study had certain limitations. Although we used the GWAS dataset, due to the lack of original data, we could not fully analyze the staging of IBD (such as IBD with periods of disease activation and remission). Therefore, additional MR analysis are still required to estimate the causal relationship between different stages of IBD and CVD outcomes. Though our results suggested that IBD does not increase the risk of CVD outcomes, we could not completely rule out the possibility. A definitive causal relationship requires more in-depth mechanism studies and RCTs in the future.

## Conclusion

In this study, we assessed the causal effect of IBD (ulcerative colitis and Crohn’s disease) on the increased risk of CVD outcomes by using a two-sample MR analysis. Our results suggested that genetically predicted IBD has no clear causal effect on CVD outcomes. Therefore, further updated MR analysis should be conducted to confirm our results when more advanced methods and more IBD patients are available to obtain less bias estimation and more accurate accuracy.

## Data availability statement

Publicly available datasets were analyzed in this study. The data of ulcerative colitis (ID: ieu-a-970), Crohn’s disease (ID: ieu-a-12), coronary artery disease (ID: ieu-a-7), coronary atherosclerosis (ID: ukb-d-I9_CORATHER), heart failure (ID: ebi-a-GCST009541), venous thromboembolism (ID: ukb-d-I9_VTE), deep venous thrombosis (ID: ukb-b-12040), atrial fibrillation (ID: ebi-a-GCST006414), pulmonary embolism (ID: ukb-d-I26), peripheral arterial disease (ID: ukb-b-4929), ischemic stroke (ID: ebi-a-GCST006908), intracerebral hemorrhage (ID: finn-b-I9_ICH), and cerebral infarction (ID: ukb-b-19350) can be obtained from https://gwas.mrcieu.ac.uk/. The data of cardiomyopathy (ID: Finngen-R7-I9-CARDMYO), subarachnoid hemorrhage (ID: Finngen-R7-I9- SAH), and atherosclerosis (ID: Finngen-R7-I9-ATHSCLE) can be obtained from https://www.finngen.fi/en. The R code to perform the MR analysis is detailed in Supplementary material 7.

## Ethics statement

Ethical review and approval was not required for this study in accordance with the local legislation and institutional requirements.

## Author contributions

XS designed the study. DX and KW wrote the manuscript. KW and AL contributed to the data analysis and data interpretation. LL and TS contributed to the revision of the manuscript. All authors contributed to the article and approved the submitted version.

## Footnotes

1
https://www.ibdgenetics.org/

2
https://mrcieu.github.io/TwoSampleMR/reference/clump_data.html

3
http://www.phenoscanner.medschl.cam.ac.uk/

4
http://github.com/MRCIEU/TwoSampleMR

5
https://shiny.cnsgenomics.com/mRnd/

## Supplementary material

Supplementary material 1 Instrumental SNPs from ulcerative colitis and Crohn’s disease GWASs.

Supplementary material 2 Mendelian randomization estimates from each method of the causal effect of inflammatory bowel disease on CVDs.

Supplementary material 3 Mendelian randomization effect size, leave-one-out analyses, and funnel plot for ulcerative colitis on CVDs.

Supplementary material 4 Mendelian randomization effect size, leave-one-out analyses, and funnel plot for Crohn’s disease on CVDs.

Supplementary material 5 Heterogeneity, level pleiotropy test, and the power of Mendelian randomization analyses.

Supplementary material 6 STROBE-MR checklist of recommended items to address in reports of MR studies.

Supplementary material 7 The R code was used to perform Mendelian randomization.

## Abbreviations

IBD: inflammatory bowel disease
CVD: cardiovascular disease
MR: Mendelian randomization
SNP: single nucleotide polymorphism
OR: odds ratio
CI: confidence interval
HERMES: Heart Failure Molecular Epidemiology for Therapeutic Targets Consortium
MRC-IEU: Medical Research Council-Integrative Epidemiology Unit.
