# Supplementary material for: Inflammatory bowel disease and cardiovascular disease: A two-sample Mendelian randomization analysis
Source: Front Cardiovasc Med. 2022 Sep 2;9:927120. doi: 10.3389/fcvm.2022.927120 (PMC9478388; doi:10.3389/fcvm.2022.927120)
Supplement: Supplementary material 1 — Instrumental SNPs from ulcerative colitis and Crohn’s disease GWASs. [file Data_Sheet_1.ZIP › Supplementary material 3.pdf]

Figure 1 Leave-one-out analysis, MR effect size and funnel plot for ulcerative colitis on coronary heart disease.

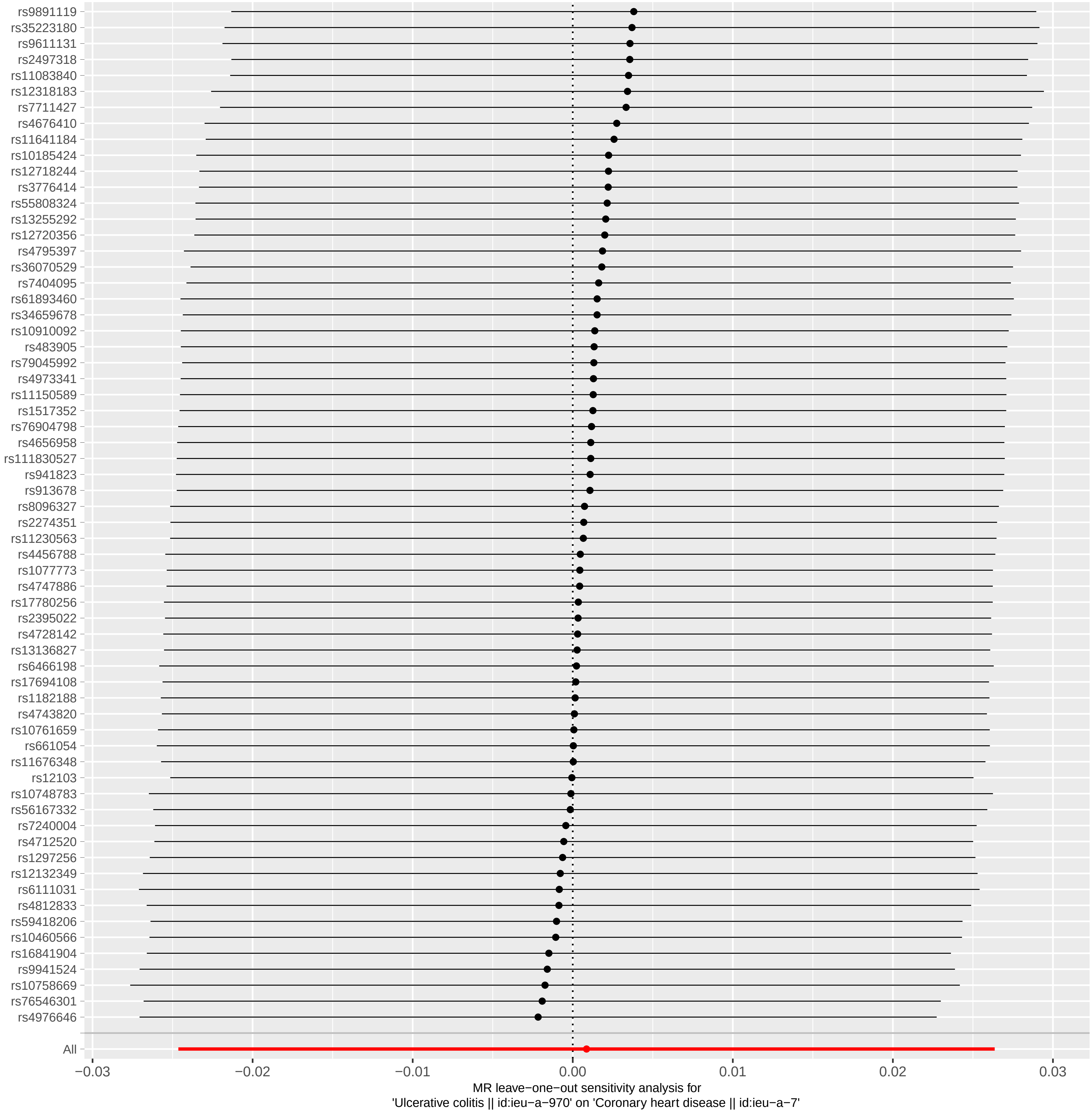

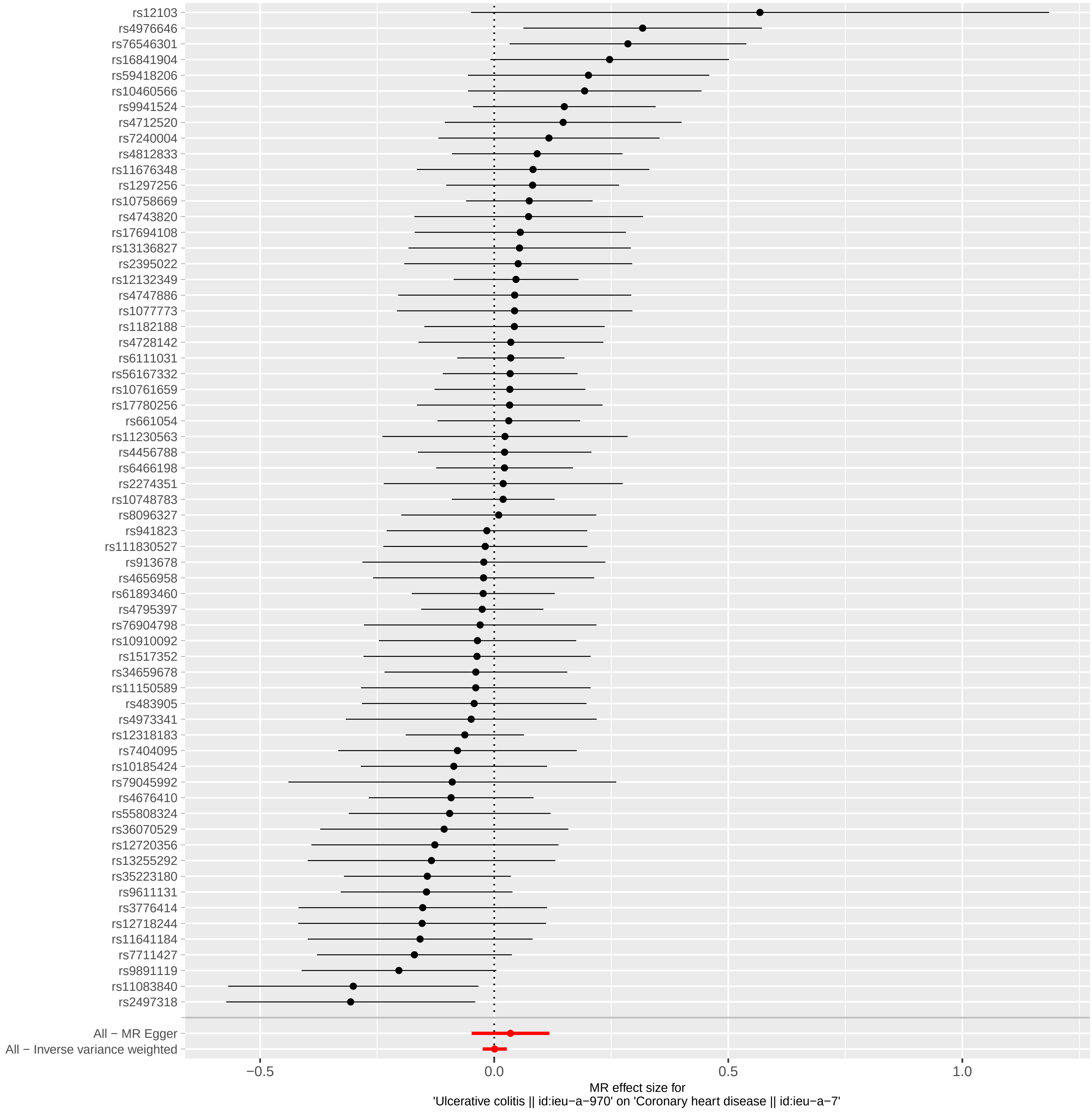

rs12103
rs4976646
rs76546301
rs16841904
rs59418206
rs10460566
rs9941524
rs4712520
rs7240004
rs4812833
rs11676348
rs1297256
rs10758669
rs4743820
rs17694108
rs13136827
rs2395022
rs12132349
rs4747886
rs1077773
rs1182188
rs4728142
rs6111031
rs56167332
rs10761659
rs17780256
rs661054
rs11230563
rs4456788
rs6466198
rs2274351
rs10748783
rs8096327
rs941823
rs111830527
rs913678
rs4656958
rs61893460
rs4795397
rs76904798
rs10910092
rs1517352
rs34659678
rs11150589
rs483905
rs4973341
rs12318183
rs7404095
rs10185424
rs79045992
rs4676410
rs55808324
rs36070529
rs12720356
rs13255292
rs35223180
rs9611131
rs3776414
rs12718244
rs11641184
rs7711427
rs9891119
rs11083840
rs2497318
All − MR Egger
All − Inverse variance weighted
−0.5
0.0
0.5
1.0
MR effect size for
'Ulcerative colitis || id:ieu−a−970' on 'Coronary heart disease || id:ieu−a−7'

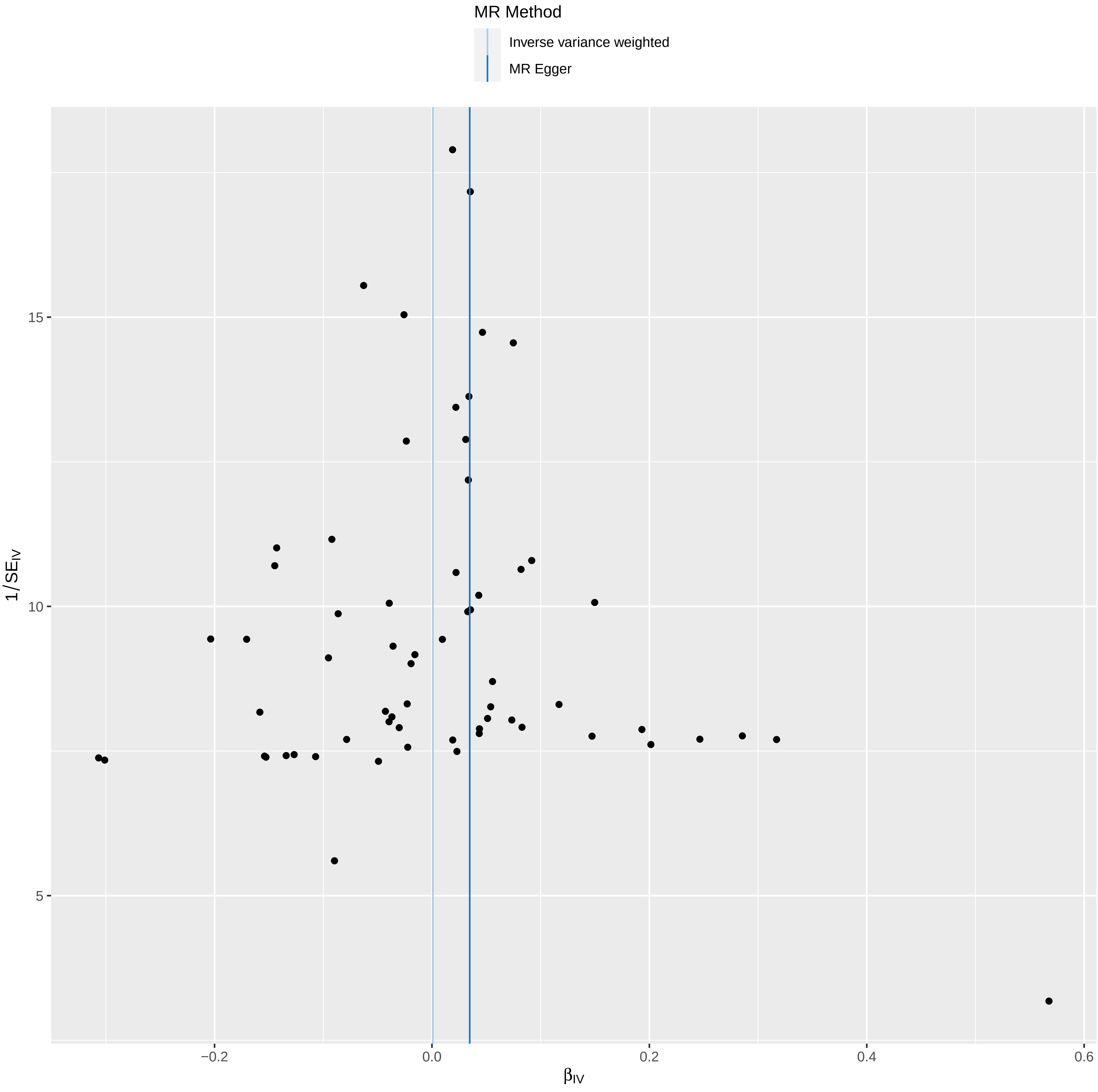

MR Method
Inverse variance weighted
MR Egger
15
10
5
$1/SE_{IV}$
−0.2
0.0
0.2
0.4
0.6
β_{IV}

Figure 2 Leave-one-out analysis, MR effect size and funnel plot for ulcerative colitis on coronary atherosclerosis.

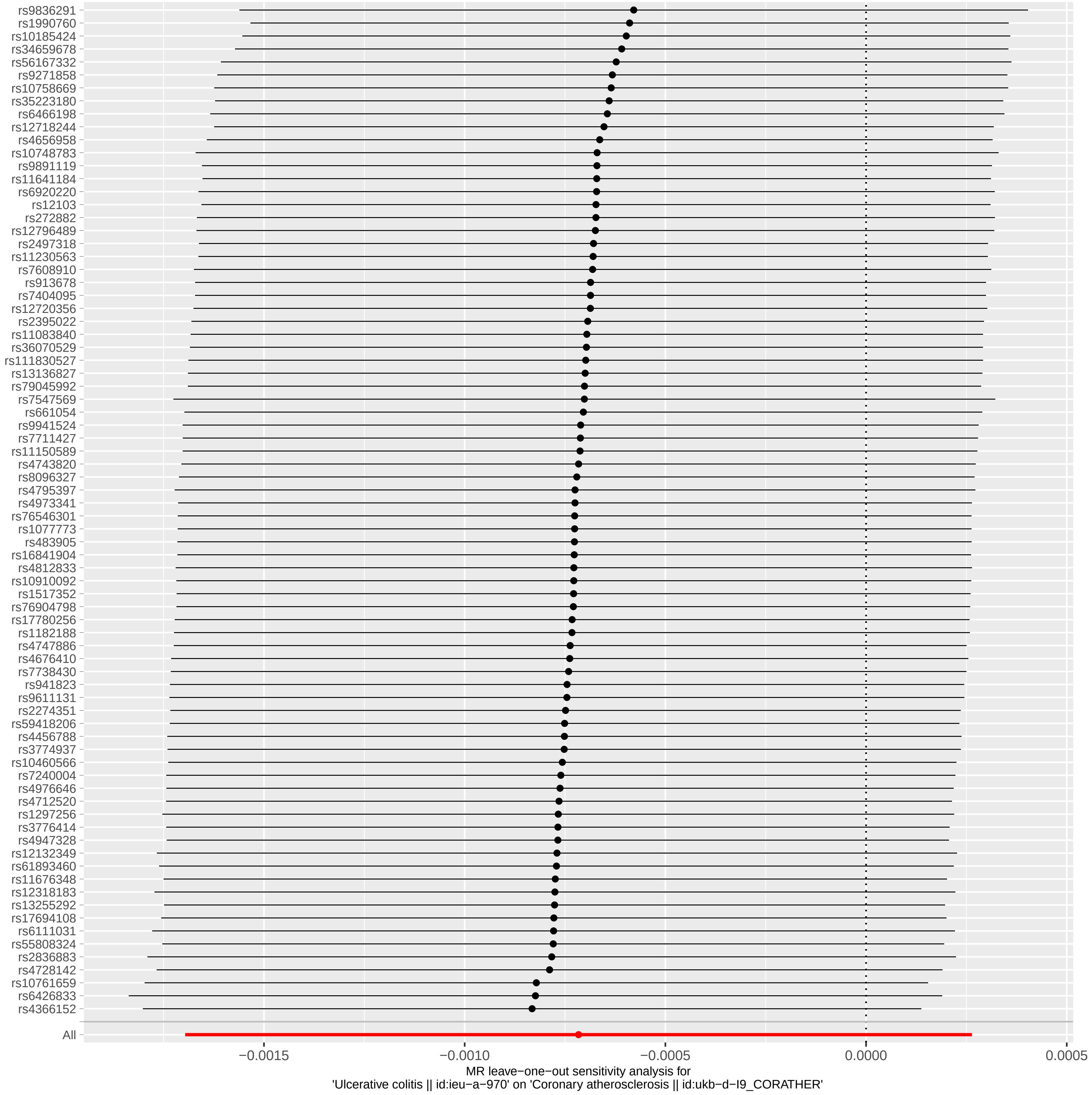

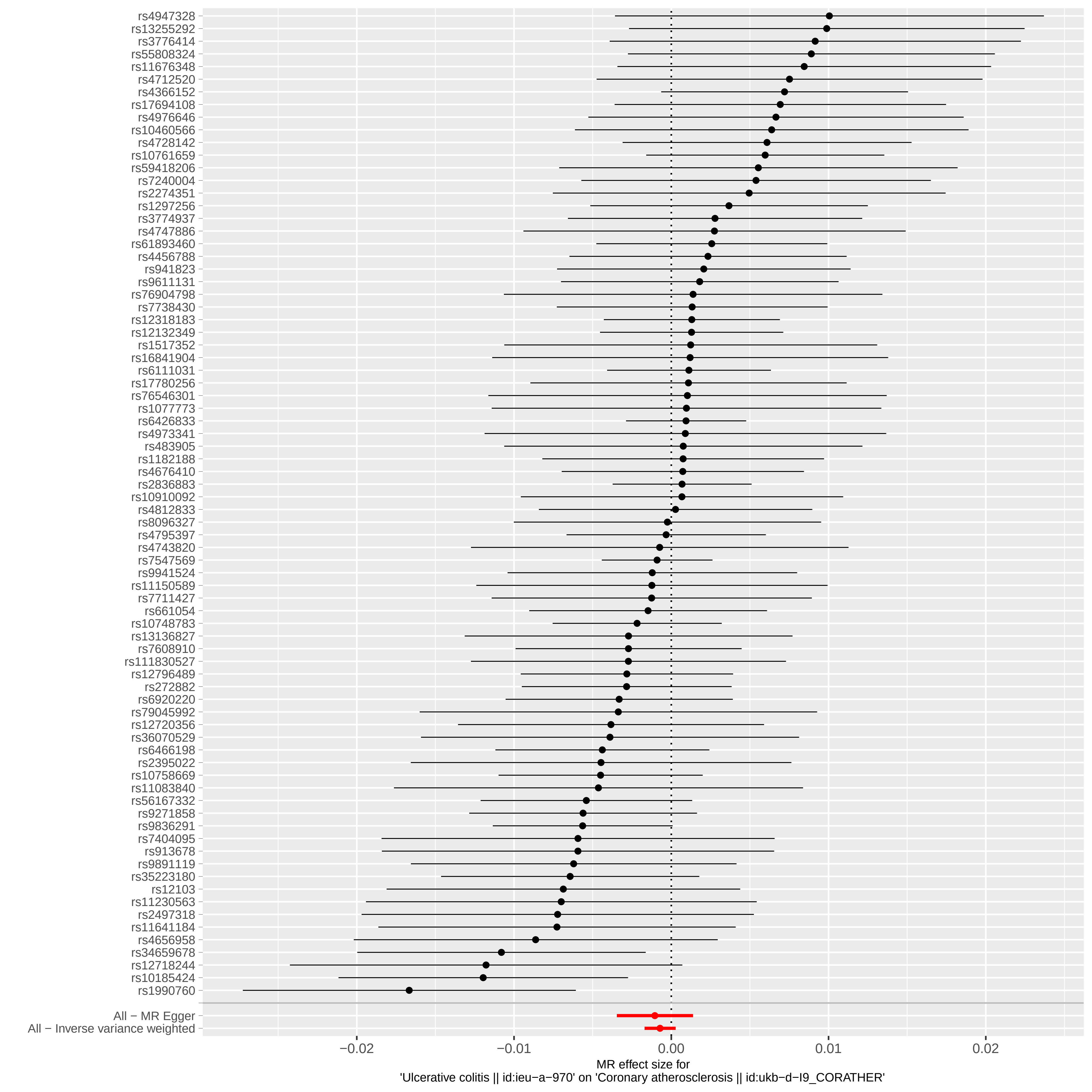

rs4947328
rs13255292
rs3776414
rs55808324
rs11676348
rs4712520
rs4366152
rs17694108
rs4976646
rs10460566
rs4728142
rs10761659
rs59418206
rs7240004
rs2274351
rs1297256
rs3774937
rs4747886
rs61893460
rs4456788
rs941823
rs9611131
rs76904798
rs7738430
rs12318183
rs12132349
rs1517352
rs16841904
rs6111031
rs17780256
rs76546301
rs1077773
rs6426833
rs4973341
rs483905
rs1182188
rs4676410
rs2836883
rs10910092
rs4812833
rs8096327
rs4795397
rs4743820
rs7547569
rs9941524
rs11150589
rs7711427
rs661054
rs10748783
rs13136827
rs7608910
rs111830527
rs12796489
rs272882
rs6920220
rs79045992
rs12720356
rs36070529
rs6466198
rs2395022
rs10758669
rs11083840
rs56167332
rs9271858
rs9836291
rs7404095
rs913678
rs9891119
rs35223180
rs12103
rs11230563
rs2497318
rs11641184
rs4656958
rs34659678
rs12718244
rs10185424
rs1990760
All − MR Egger
All − Inverse variance weighted
−0.02
−0.01
0.00
0.01
0.02
MR effect size for
'Ulcerative colitis || id:ieu−a−970' on 'Coronary atherosclerosis || id:ukb−d−I9_CORATHER'

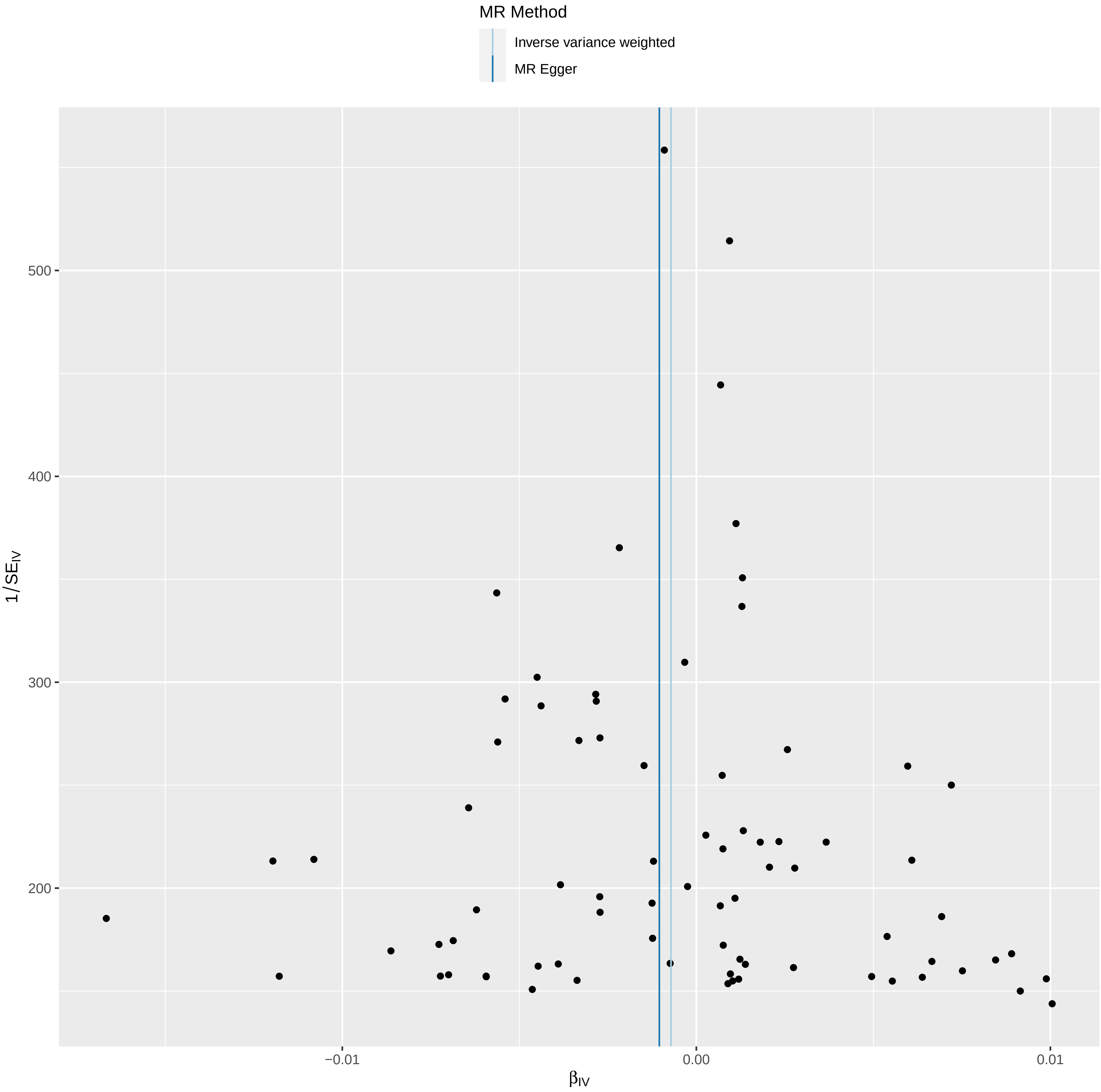

MR Method
Inverse variance weighted
MR Egger
500
400
300
200
1/SE$_{IV}$
−0.01
0.00
0.01
β$_{IV}$

Figure 3 Leave-one-out analysis, MR effect size and funnel plot for ulcerative colitis on heart failure.

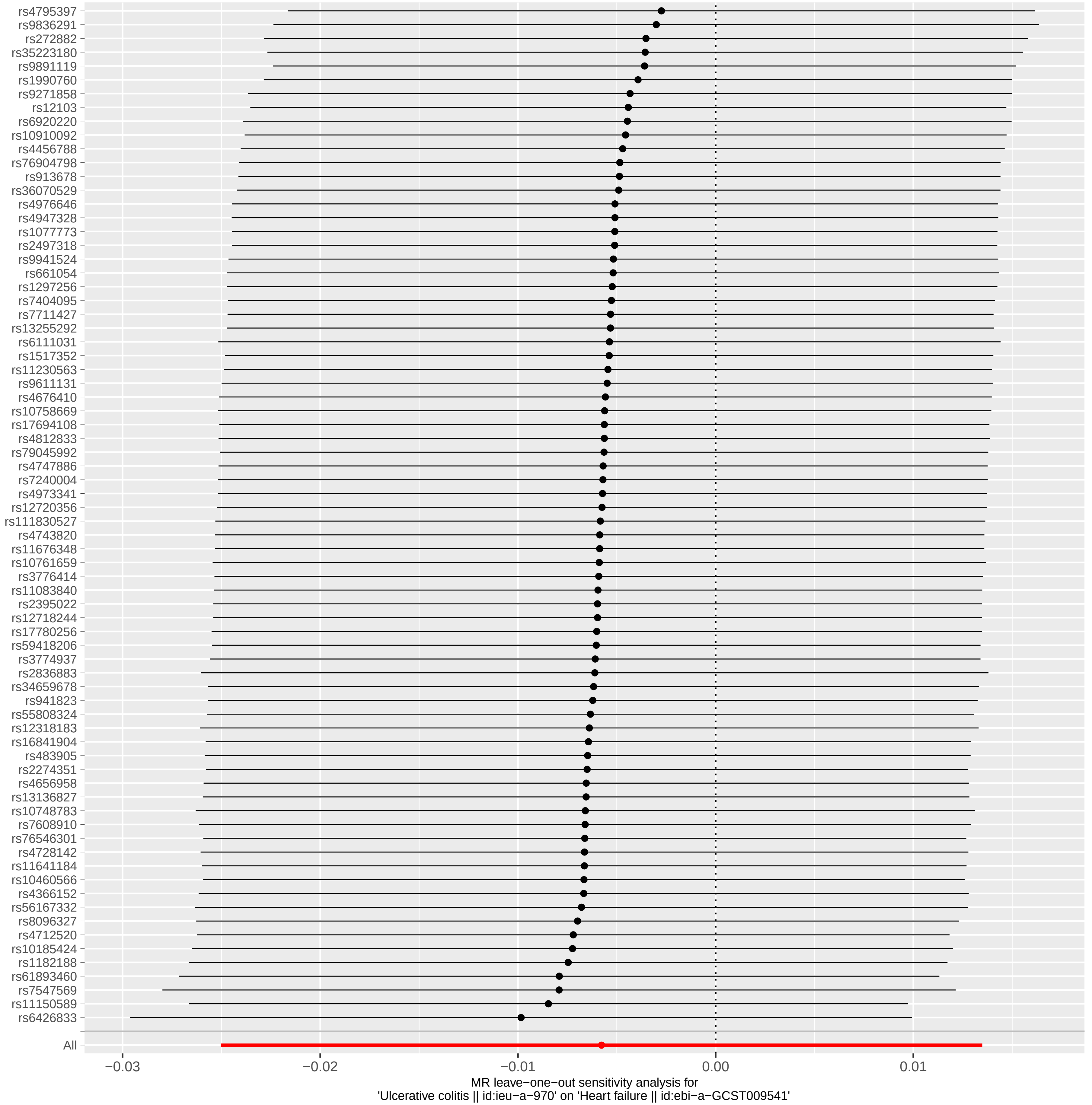

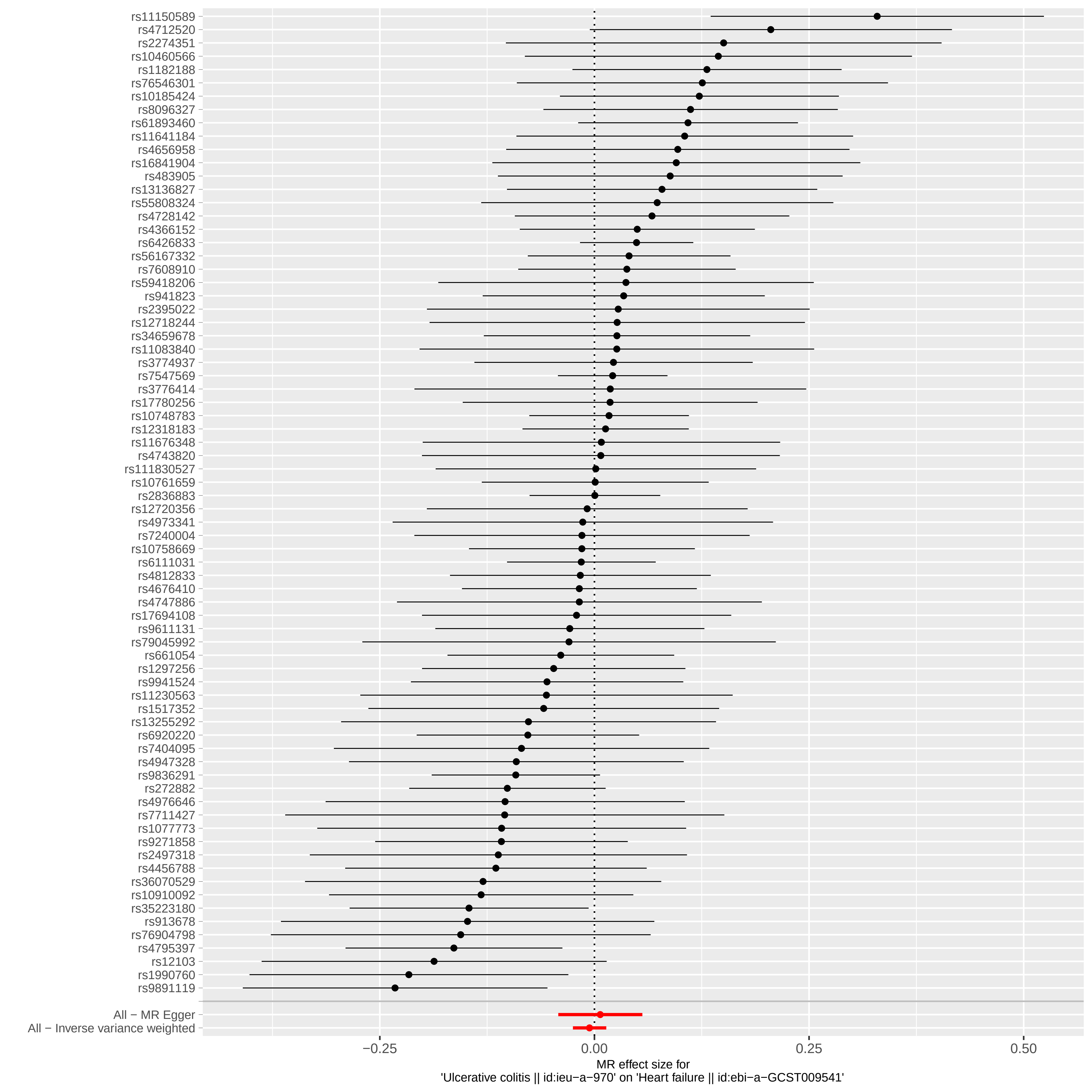

rs11150589
rs4712520
rs2274351
rs10460566
rs1182188
rs76546301
rs10185424
rs8096327
rs61893460
rs11641184
rs4656958
rs16841904
rs483905
rs13136827
rs55808324
rs4728142
rs4366152
rs6426833
rs56167332
rs7608910
rs59418206
rs941823
rs2395022
rs12718244
rs34659678
rs11083840
rs3774937
rs7547569
rs3776414
rs17780256
rs10748783
rs12318183
rs11676348
rs4743820
rs111830527
rs10761659
rs2836883
rs12720356
rs4973341
rs7240004
rs10758669
rs6111031
rs4812833
rs4676410
rs4747886
rs17694108
rs9611131
rs79045992
rs661054
rs1297256
rs9941524
rs11230563
rs1517352
rs13255292
rs6920220
rs7404095
rs4947328
rs9836291
rs272882
rs4976646
rs7711427
rs1077773
rs9271858
rs2497318
rs4456788
rs36070529
rs10910092
rs35223180
rs913678
rs76904798
rs4795397
rs12103
rs1990760
rs9891119
All − MR Egger
All − Inverse variance weighted
−0.25
0.00
0.25
0.50
MR effect size for
'Ulcerative colitis || id:ieu−a−970' on 'Heart failure || id:ebi−a−GCST009541'

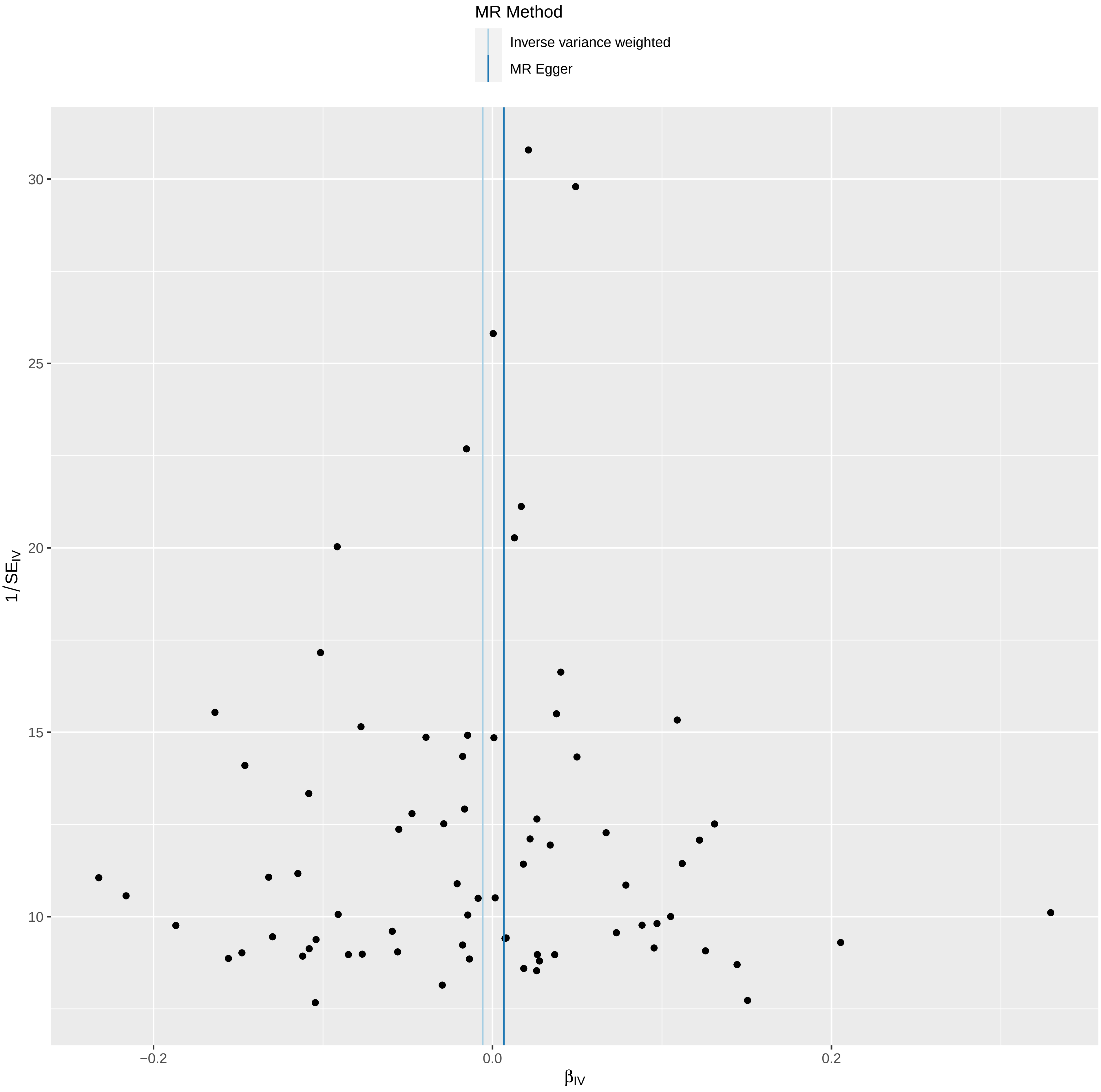
MR Method
Inverse variance weighted
MR Egger
30
25
20
15
10
1/SE_IV
−0.2
0.0
0.2
β_IV

Figure 4 Leave-one-out analysis, MR effect size and funnel plot for ulcerative colitis on cardiomyopathy.

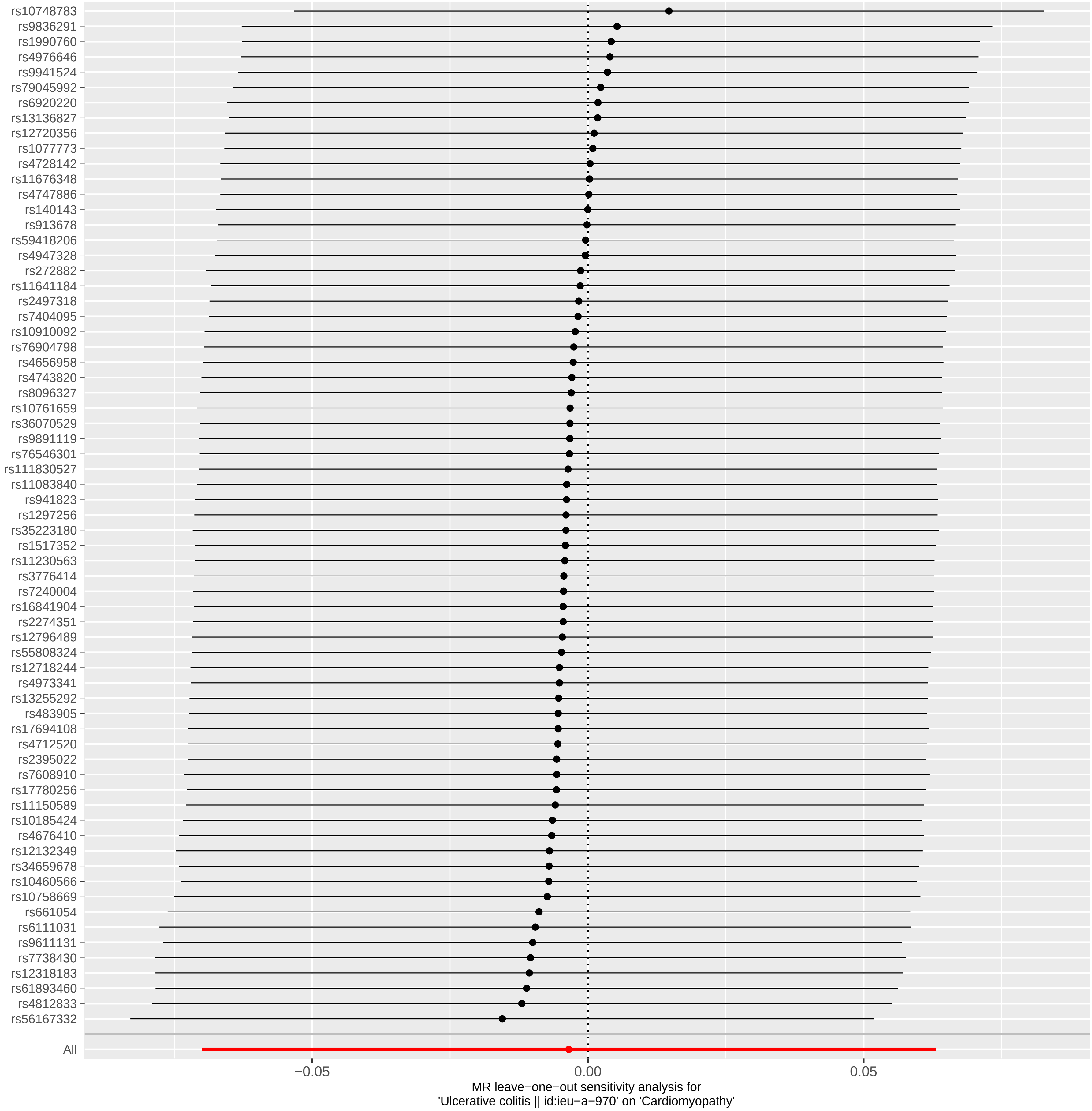

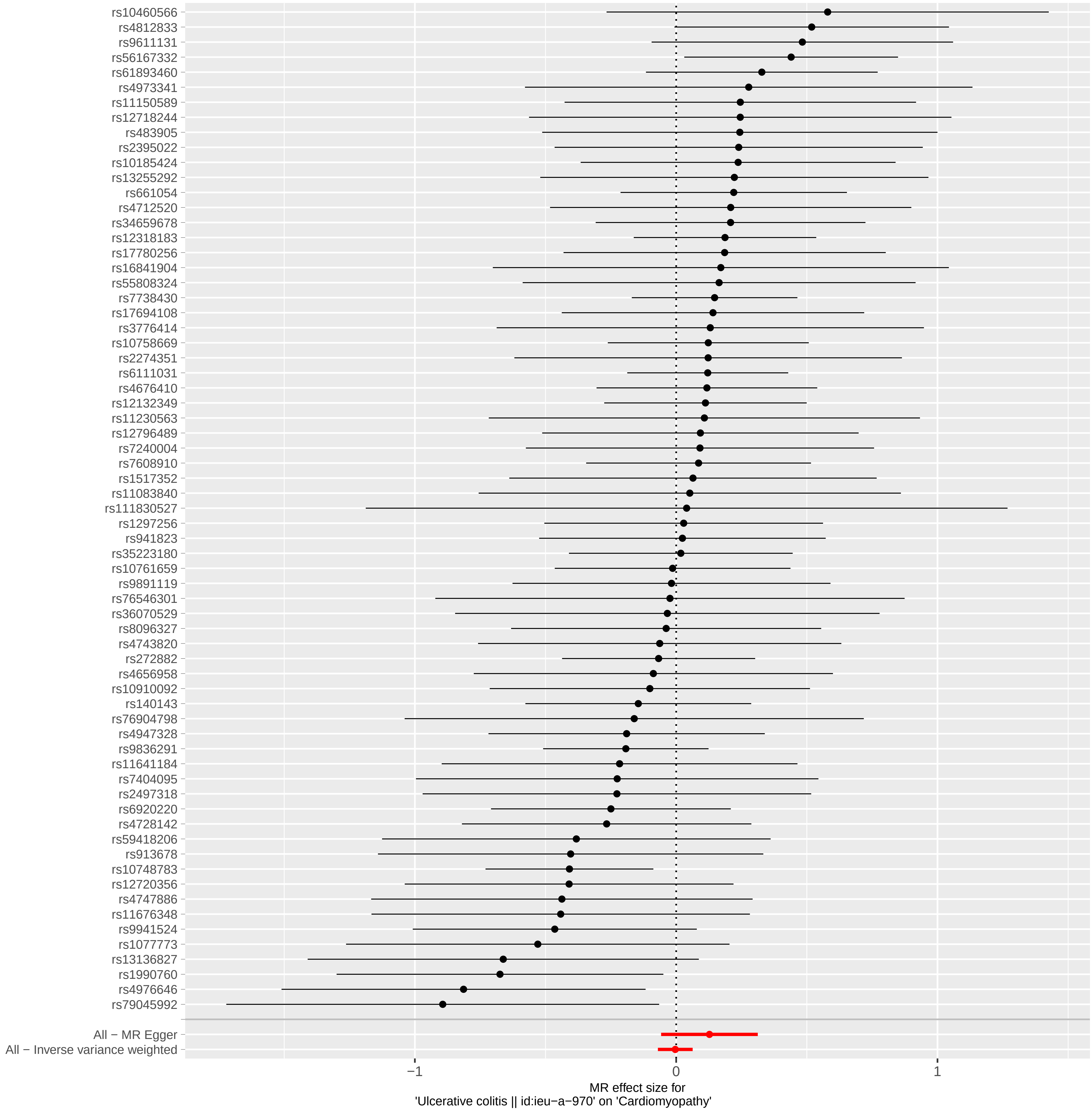
rs10460566
rs4812833
rs9611131
rs56167332
rs61893460
rs4973341
rs11150589
rs12718244
rs483905
rs2395022
rs10185424
rs13255292
rs661054
rs4712520
rs34659678
rs12318183
rs17780256
rs16841904
rs55808324
rs7738430
rs17694108
rs3776414
rs10758669
rs2274351
rs6111031
rs4676410
rs12132349
rs11230563
rs12796489
rs7240004
rs7608910
rs1517352
rs11083840
rs111830527
rs1297256
rs941823
rs35223180
rs10761659
rs9891119
rs76546301
rs36070529
rs8096327
rs4743820
rs272882
rs4656958
rs10910092
rs140143
rs76904798
rs4947328
rs9836291
rs11641184
rs7404095
rs2497318
rs6920220
rs4728142
rs59418206
rs913678
rs10748783
rs12720356
rs4747886
rs11676348
rs9941524
rs1077773
rs13136827
rs1990760
rs4976646
rs79045992
All − MR Egger
All − Inverse variance weighted
−1
0
1
MR effect size for
'Ulcerative colitis || id:ieu−a−970' on 'Cardiomyopathy'

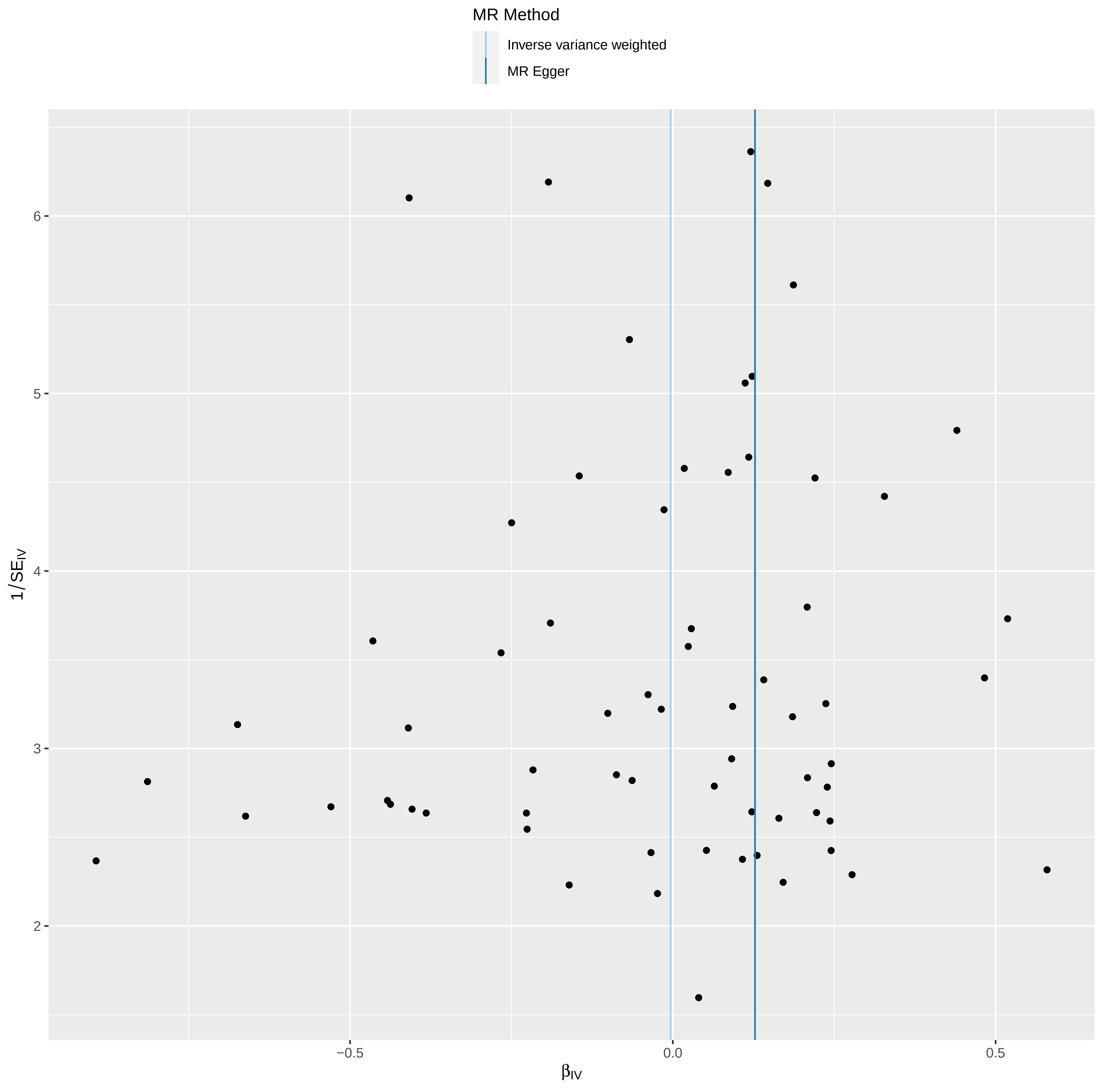

MR Method
Inverse variance weighted
MR Egger
6
5
4
3
2
$1/SE_{IV}$
−0.5
0.0
0.5
β_{IV}

Figure 5 Leave-one-out analysis, MR effect size and funnel plot for ulcerative colitis on venous thromboembolism.

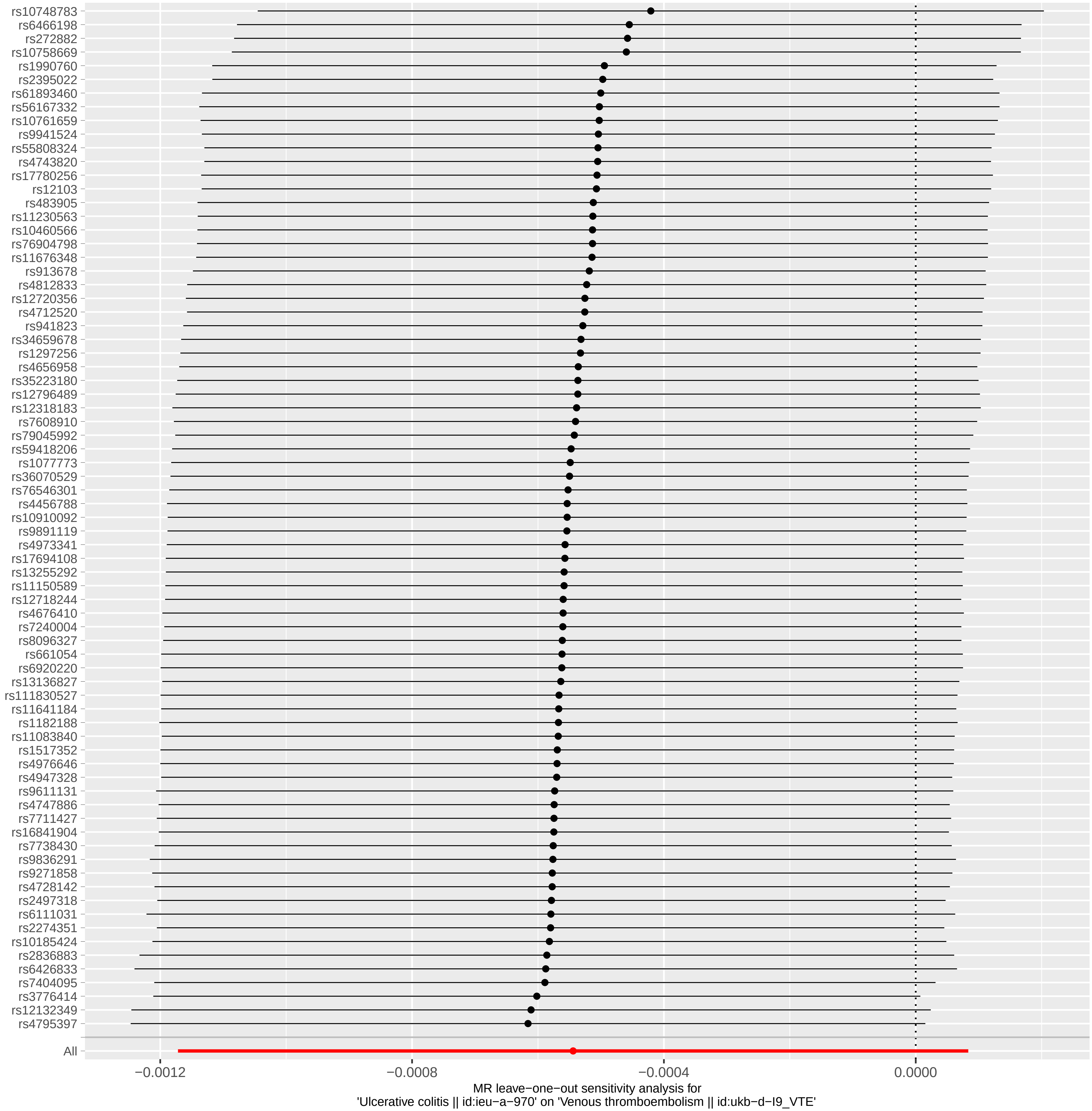

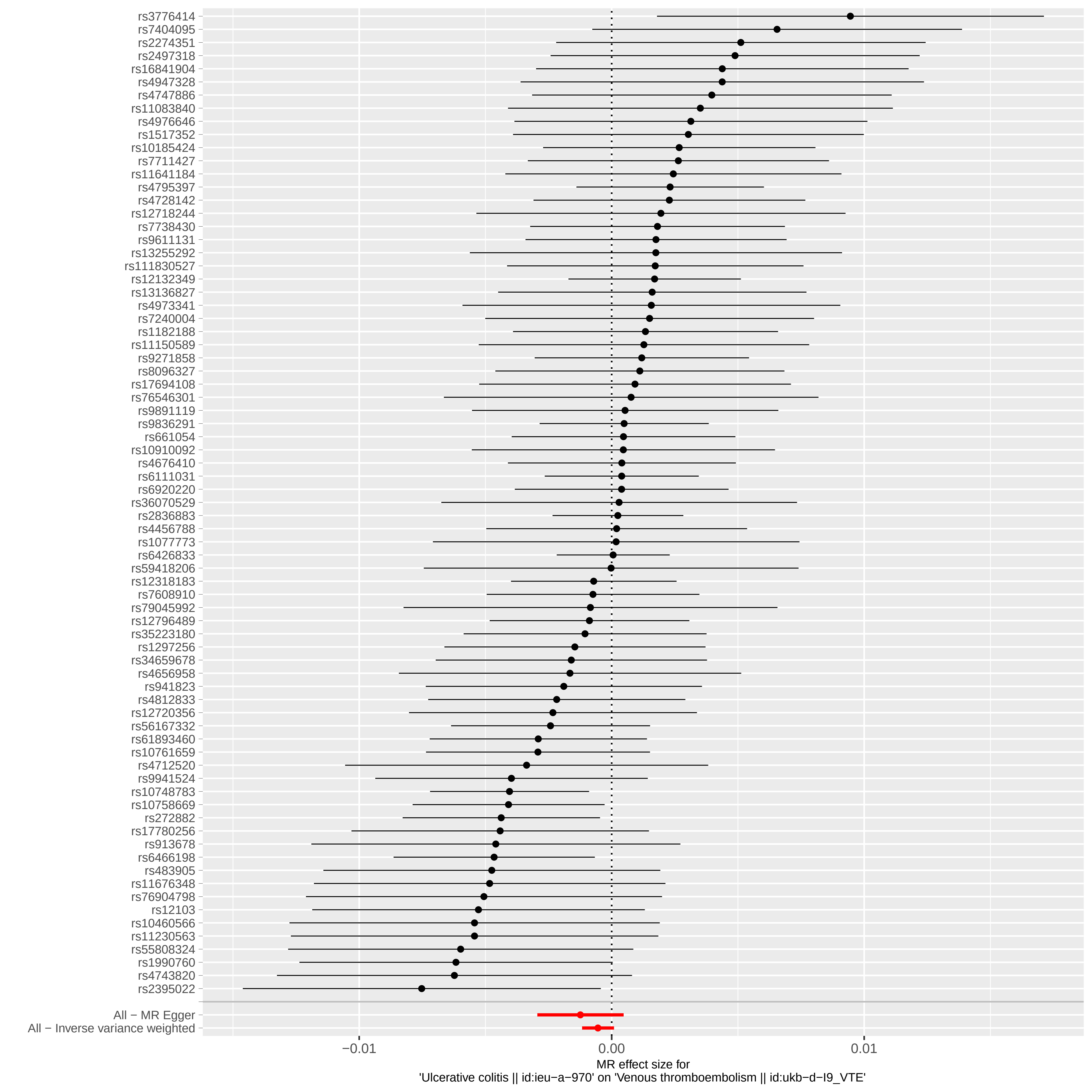
rs3776414
rs7404095
rs2274351
rs2497318
rs16841904
rs4947328
rs4747886
rs11083840
rs4976646
rs1517352
rs10185424
rs7711427
rs11641184
rs4795397
rs4728142
rs12718244
rs7738430
rs9611131
rs13255292
rs111830527
rs12132349
rs13136827
rs4973341
rs7240004
rs1182188
rs11150589
rs9271858
rs8096327
rs17694108
rs76546301
rs9891119
rs9836291
rs661054
rs10910092
rs4676410
rs6111031
rs6920220
rs36070529
rs2836883
rs4456788
rs1077773
rs6426833
rs59418206
rs12318183
rs7608910
rs79045992
rs12796489
rs35223180
rs1297256
rs34659678
rs4656958
rs941823
rs4812833
rs12720356
rs56167332
rs61893460
rs10761659
rs4712520
rs9941524
rs10748783
rs10758669
rs272882
rs17780256
rs913678
rs6466198
rs483905
rs11676348
rs76904798
rs12103
rs10460566
rs11230563
rs55808324
rs1990760
rs4743820
rs2395022
All − MR Egger
All − Inverse variance weighted
−0.01
0.00
0.01
MR effect size for
'Ulcerative colitis || id:ieu−a−970' on 'Venous thromboembolism || id:ukb−d−I9_VTE'

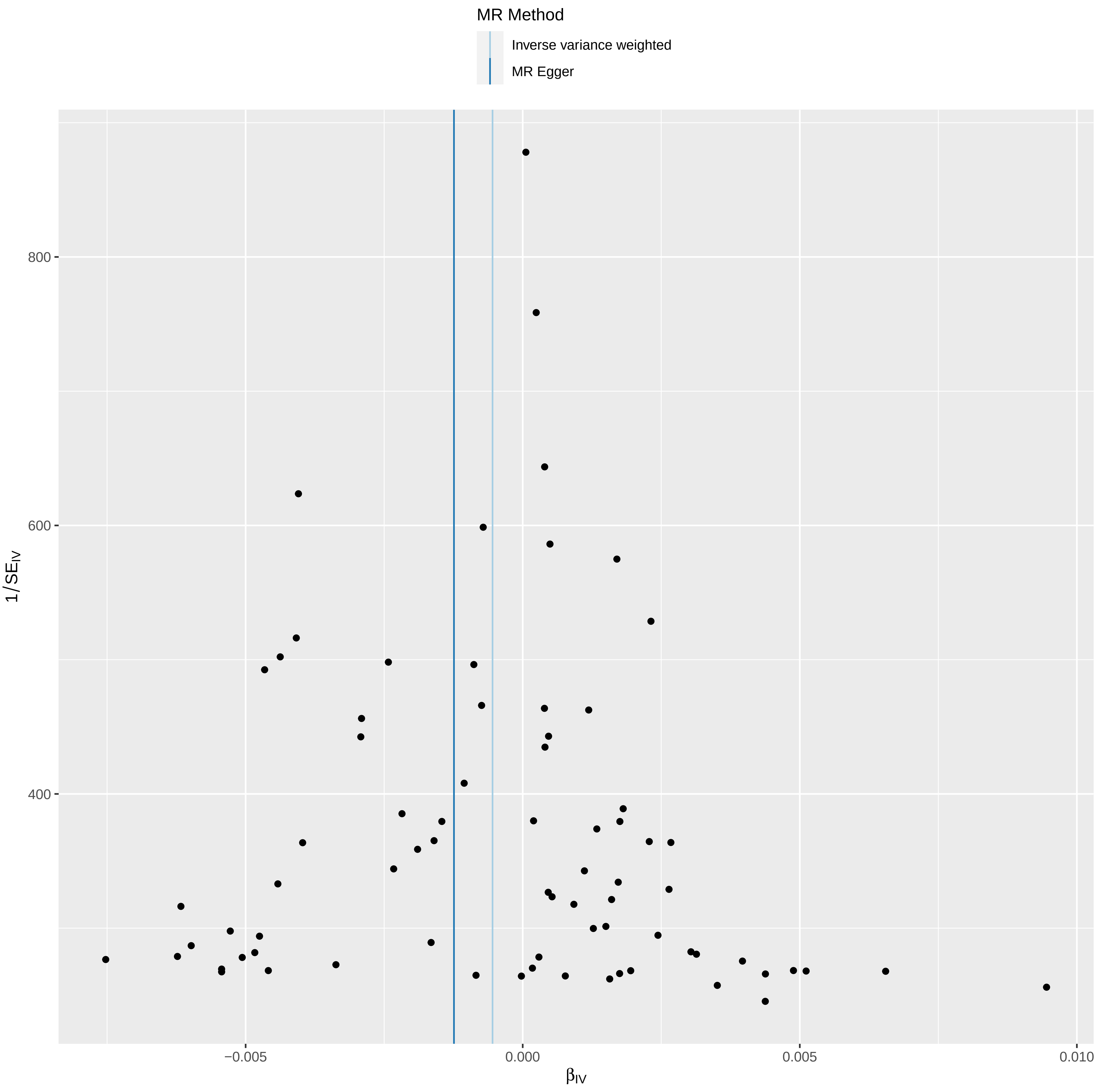
MR Method
Inverse variance weighted
MR Egger
800
600
400
1/SE_IV
−0.005
0.000
0.005
0.010
β_IV

Figure 6 Leave-one-out analysis, MR effect size and funnel plot for ulcerative colitis on deep venous thrombosis.

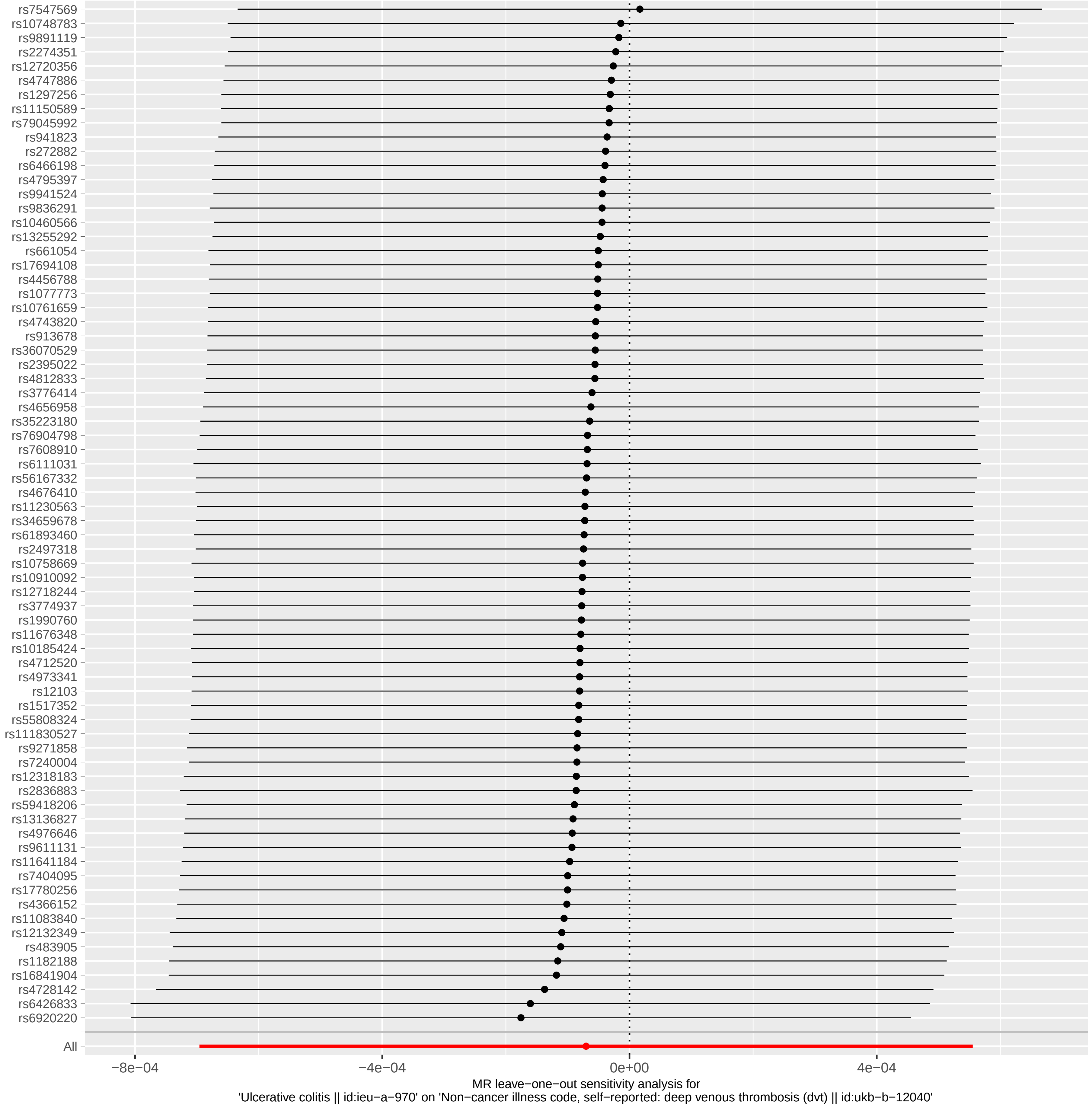

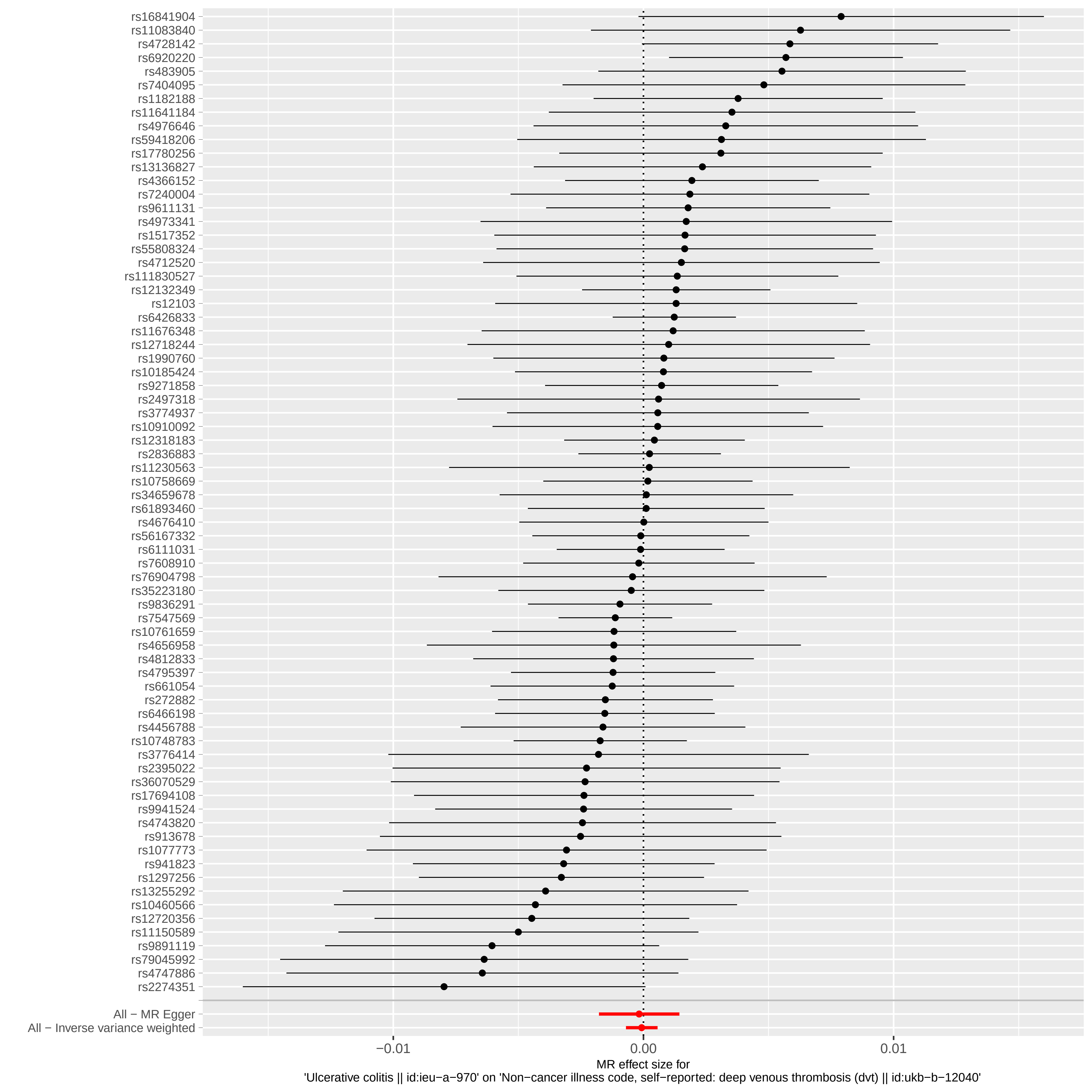

rs16841904
rs11083840
rs4728142
rs6920220
rs483905
rs7404095
rs1182188
rs11641184
rs4976646
rs59418206
rs17780256
rs13136827
rs4366152
rs7240004
rs9611131
rs4973341
rs1517352
rs55808324
rs4712520
rs111830527
rs12132349
rs12103
rs6426833
rs11676348
rs12718244
rs1990760
rs10185424
rs9271858
rs2497318
rs3774937
rs10910092
rs12318183
rs2836883
rs11230563
rs10758669
rs34659678
rs61893460
rs4676410
rs56167332
rs6111031
rs7608910
rs76904798
rs35223180
rs9836291
rs7547569
rs10761659
rs4656958
rs4812833
rs4795397
rs661054
rs272882
rs6466198
rs4456788
rs10748783
rs3776414
rs2395022
rs36070529
rs17694108
rs9941524
rs4743820
rs913678
rs1077773
rs941823
rs1297256
rs13255292
rs10460566
rs12720356
rs11150589
rs9891119
rs79045992
rs4747886
rs2274351
All − MR Egger
All − Inverse variance weighted
−0.01
0.00
0.01
MR effect size for
'Ulcerative colitis || id:ieu−a−970' on 'Non−cancer illness code, self−reported: deep venous thrombosis (dvt) || id:ukb−b−12040'

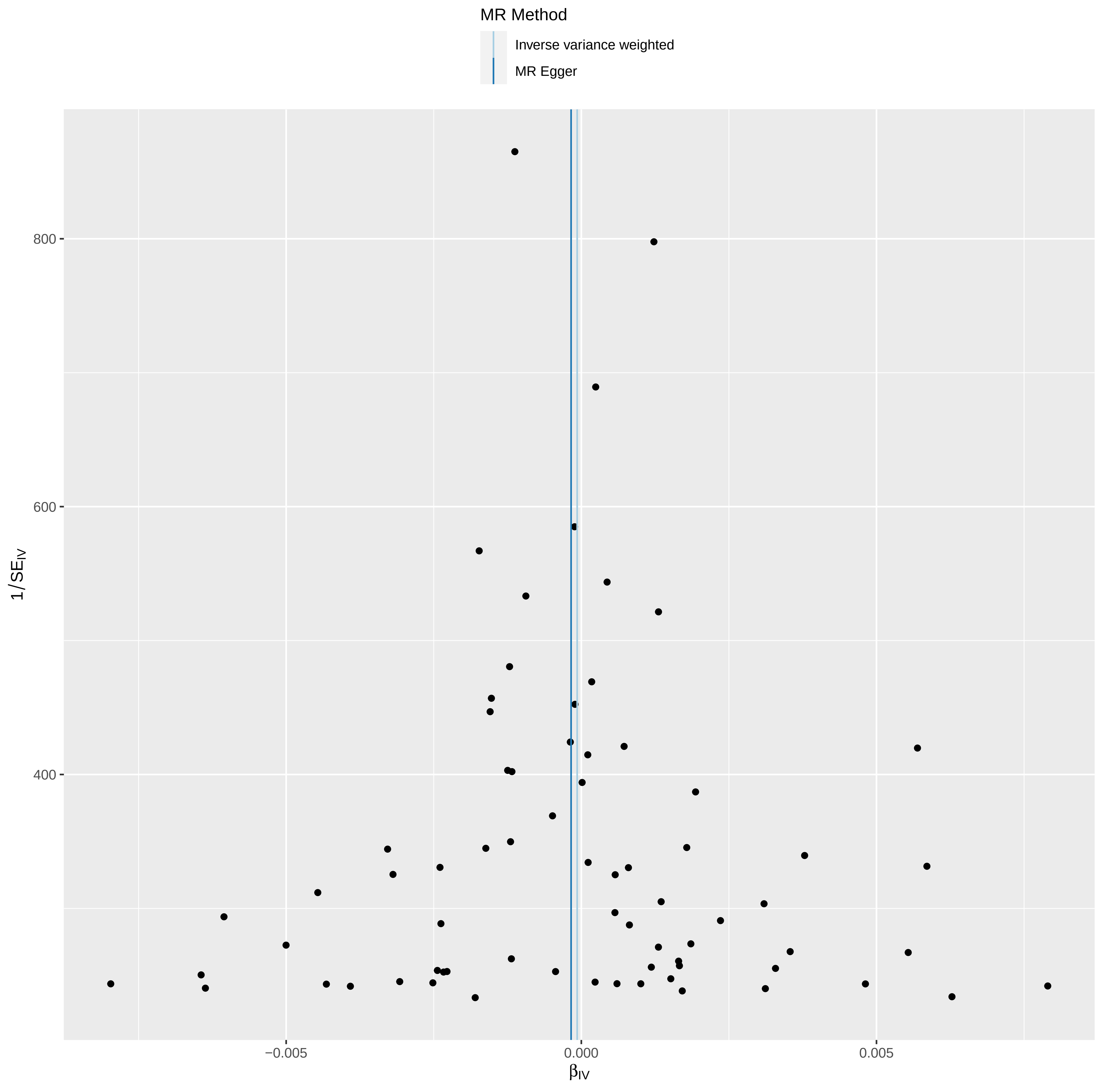
MR Method
Inverse variance weighted
MR Egger
800
600
400
1/SE_IV
−0.005
0.000
0.005
β_IV

Figure 7 Leave-one-out analysis, MR effect size and funnel plot for ulcerative colitis on atrial fibrillation.

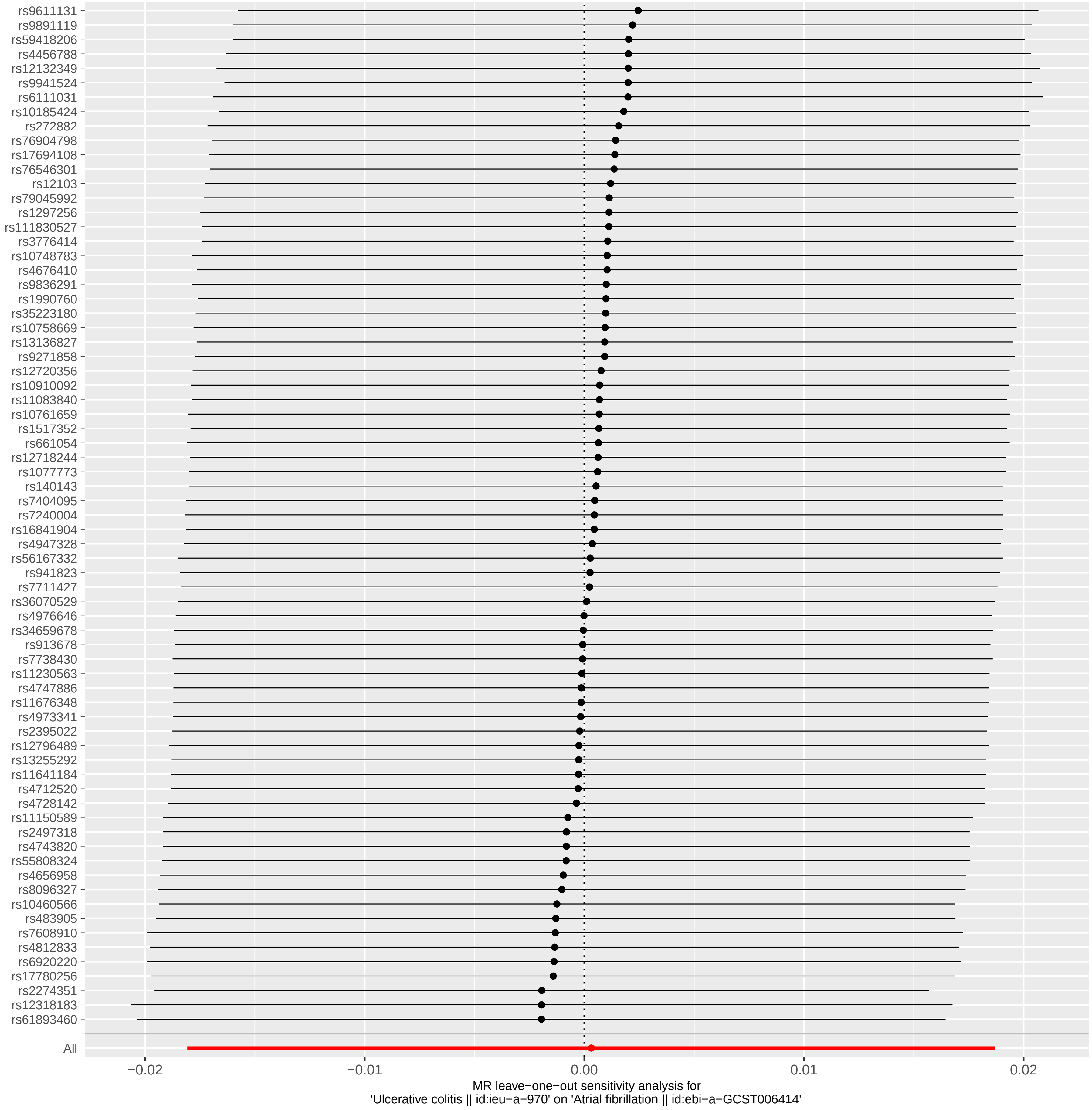

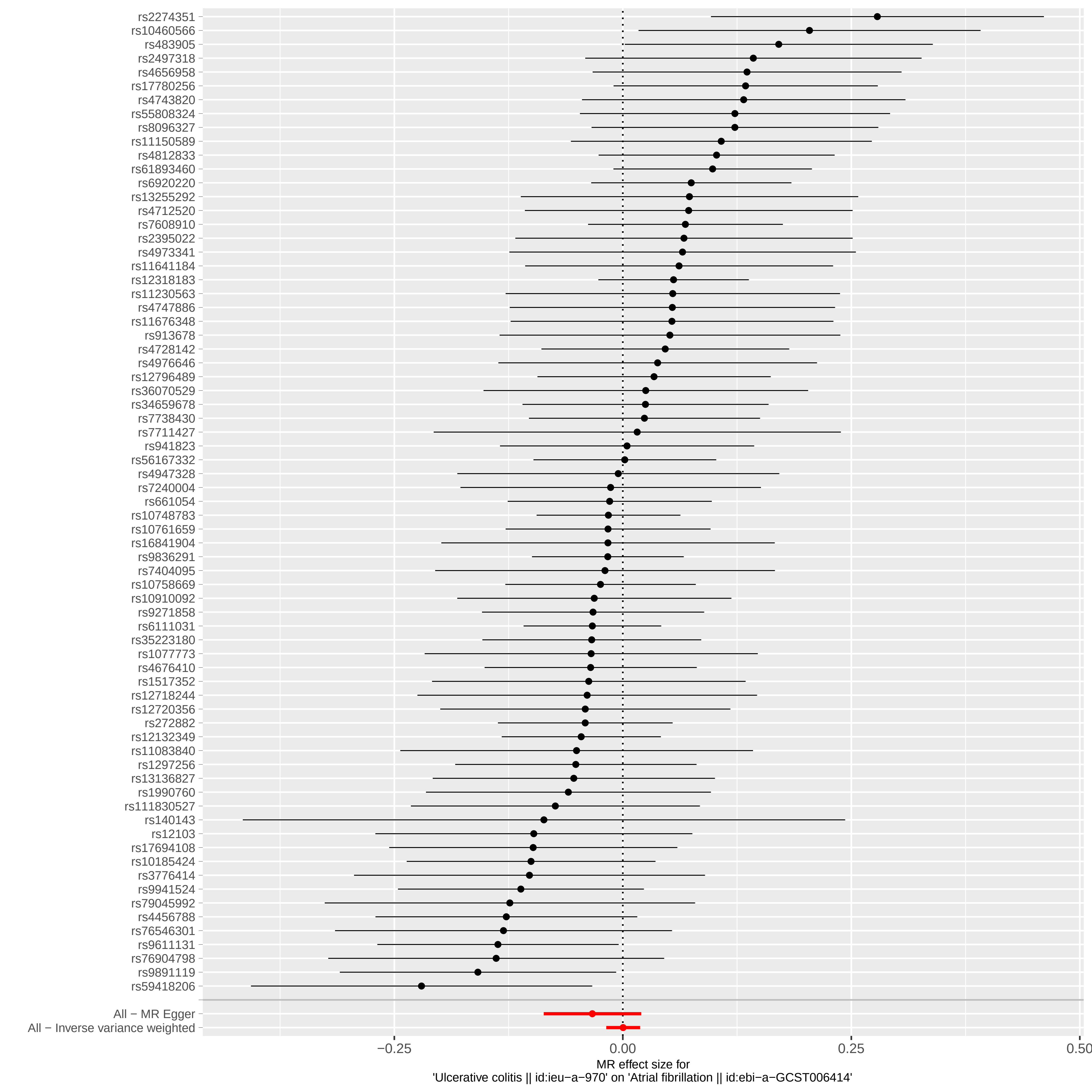

rs2274351
rs10460566
rs483905
rs2497318
rs4656958
rs17780256
rs4743820
rs55808324
rs8096327
rs11150589
rs4812833
rs61893460
rs6920220
rs13255292
rs4712520
rs7608910
rs2395022
rs4973341
rs11641184
rs12318183
rs11230563
rs4747886
rs11676348
rs913678
rs4728142
rs4976646
rs12796489
rs36070529
rs34659678
rs7738430
rs7711427
rs941823
rs56167332
rs4947328
rs7240004
rs661054
rs10748783
rs10761659
rs16841904
rs9836291
rs7404095
rs10758669
rs10910092
rs9271858
rs6111031
rs35223180
rs1077773
rs4676410
rs1517352
rs12718244
rs12720356
rs272882
rs12132349
rs11083840
rs1297256
rs13136827
rs1990760
rs111830527
rs140143
rs12103
rs17694108
rs10185424
rs3776414
rs9941524
rs79045992
rs4456788
rs76546301
rs9611131
rs76904798
rs9891119
rs59418206
All − MR Egger
All − Inverse variance weighted
−0.25
0.00
0.25
0.50
MR effect size for
'Ulcerative colitis || id:ieu−a−970' on 'Atrial fibrillation || id:ebi−a−GCST006414'

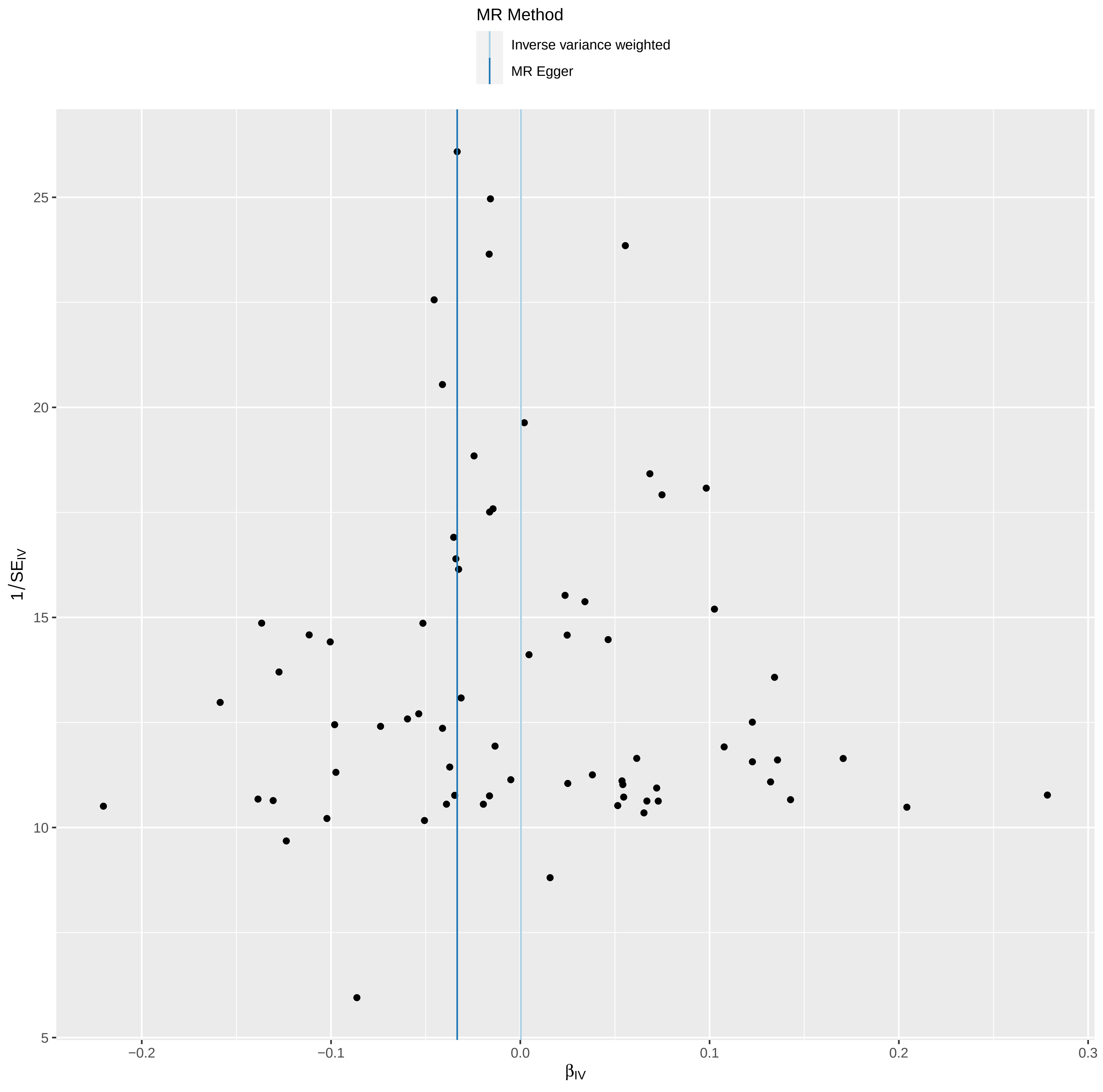
MR Method
Inverse variance weighted
MR Egger
1/SE$_{IV}$
β$_{IV}$
25
20
15
10
5
−0.2
−0.1
0.0
0.1
0.2
0.3

Figure 8 Leave-one-out analysis, MR effect size and funnel plot for ulcerative colitis on pulmonary embolism.

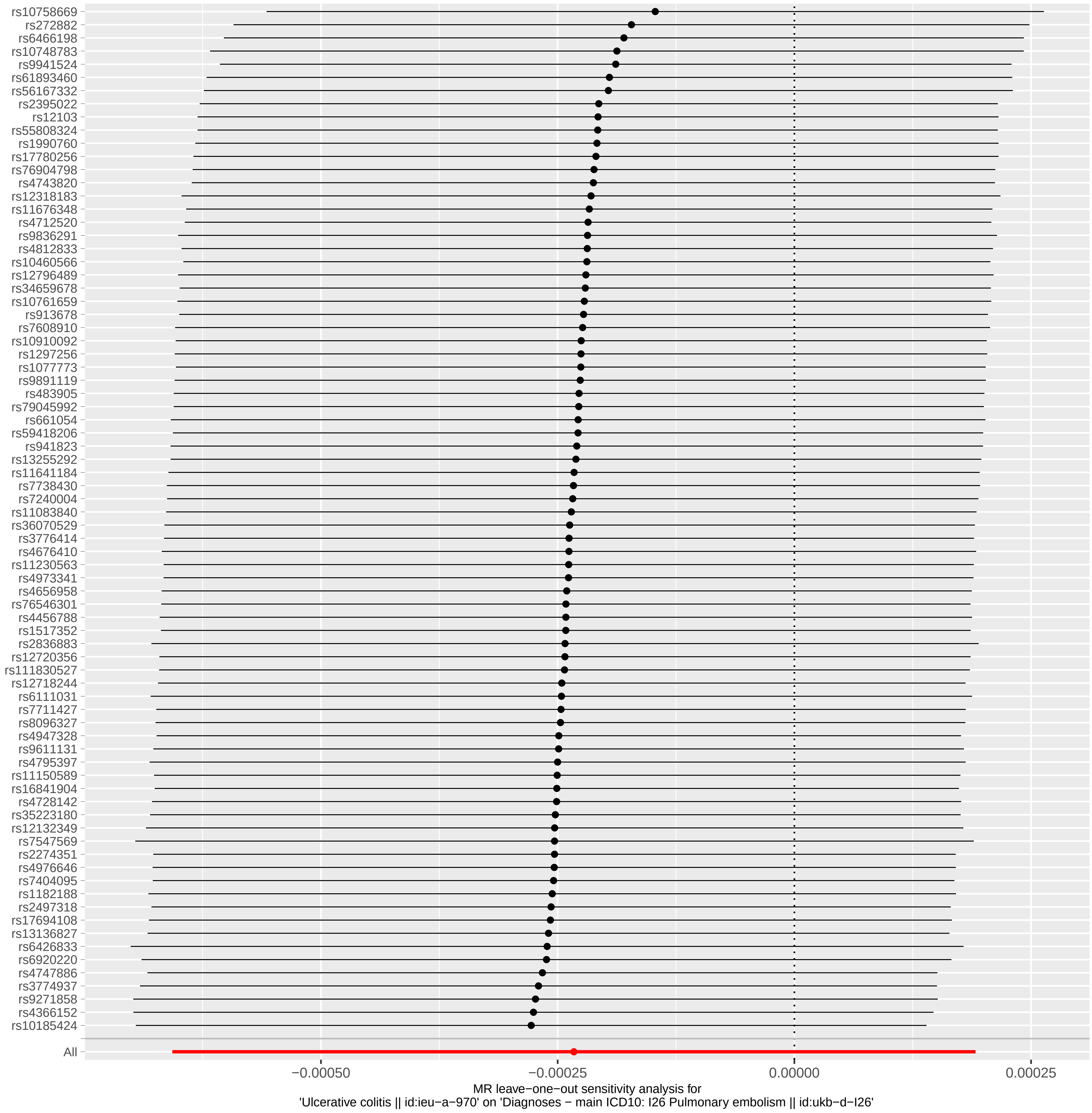

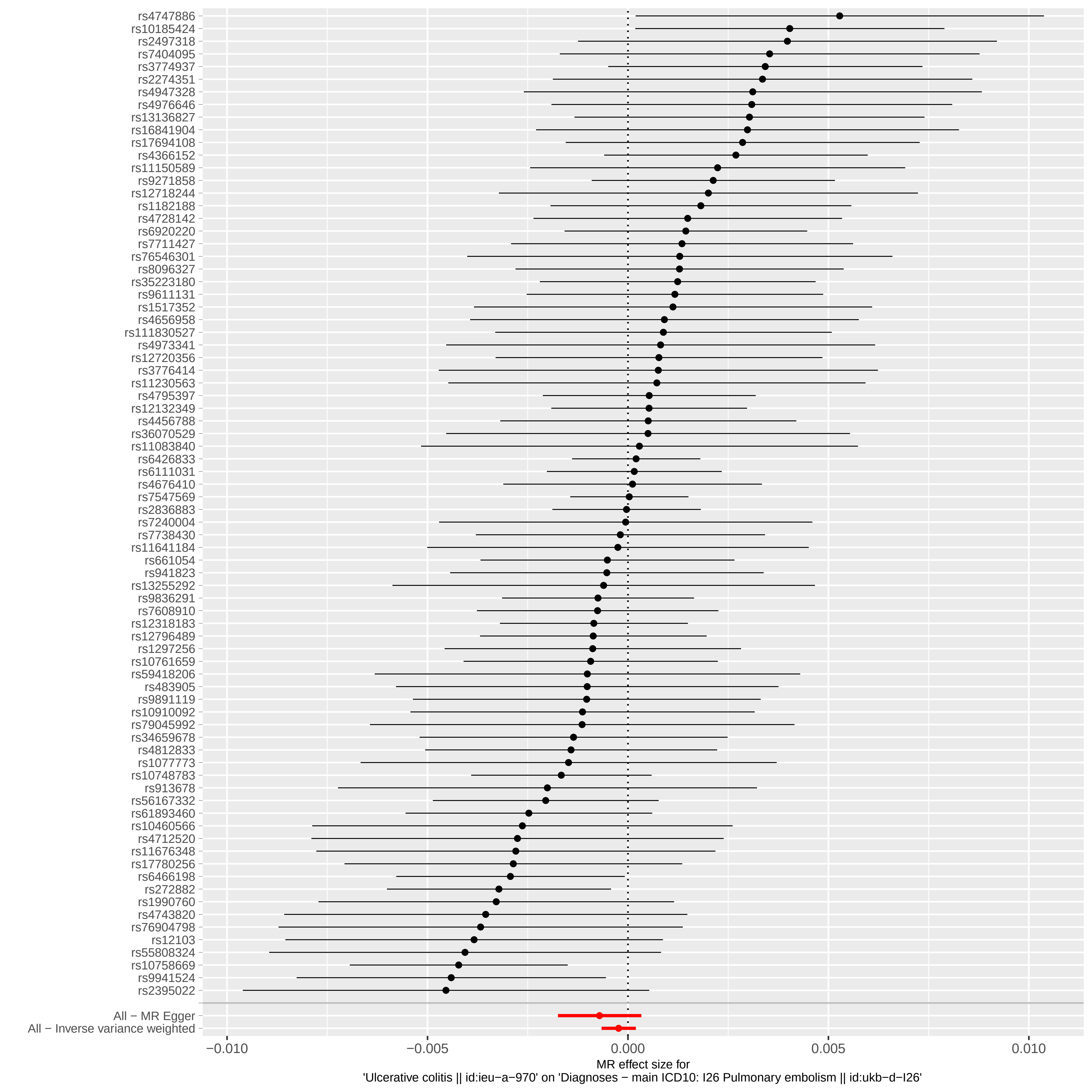

rs4747886
rs10185424
rs2497318
rs7404095
rs3774937
rs2274351
rs4947328
rs4976646
rs13136827
rs16841904
rs17694108
rs4366152
rs11150589
rs9271858
rs12718244
rs1182188
rs4728142
rs6920220
rs7711427
rs76546301
rs8096327
rs35223180
rs9611131
rs1517352
rs4656958
rs111830527
rs4973341
rs12720356
rs3776414
rs11230563
rs4795397
rs12132349
rs4456788
rs36070529
rs11083840
rs6426833
rs6111031
rs4676410
rs7547569
rs2836883
rs7240004
rs7738430
rs11641184
rs661054
rs941823
rs13255292
rs9836291
rs7608910
rs12318183
rs12796489
rs1297256
rs10761659
rs59418206
rs483905
rs9891119
rs10910092
rs79045992
rs34659678
rs4812833
rs1077773
rs10748783
rs913678
rs56167332
rs61893460
rs10460566
rs4712520
rs11676348
rs17780256
rs6466198
rs272882
rs1990760
rs4743820
rs76904798
rs12103
rs55808324
rs10758669
rs9941524
rs2395022
All − MR Egger
All − Inverse variance weighted
−0.010
−0.005
0.000
0.005
0.010
MR effect size for
'Ulcerative colitis || id:ieu−a−970' on 'Diagnoses − main ICD10: I26 Pulmonary embolism || id:ukb−d−I26'

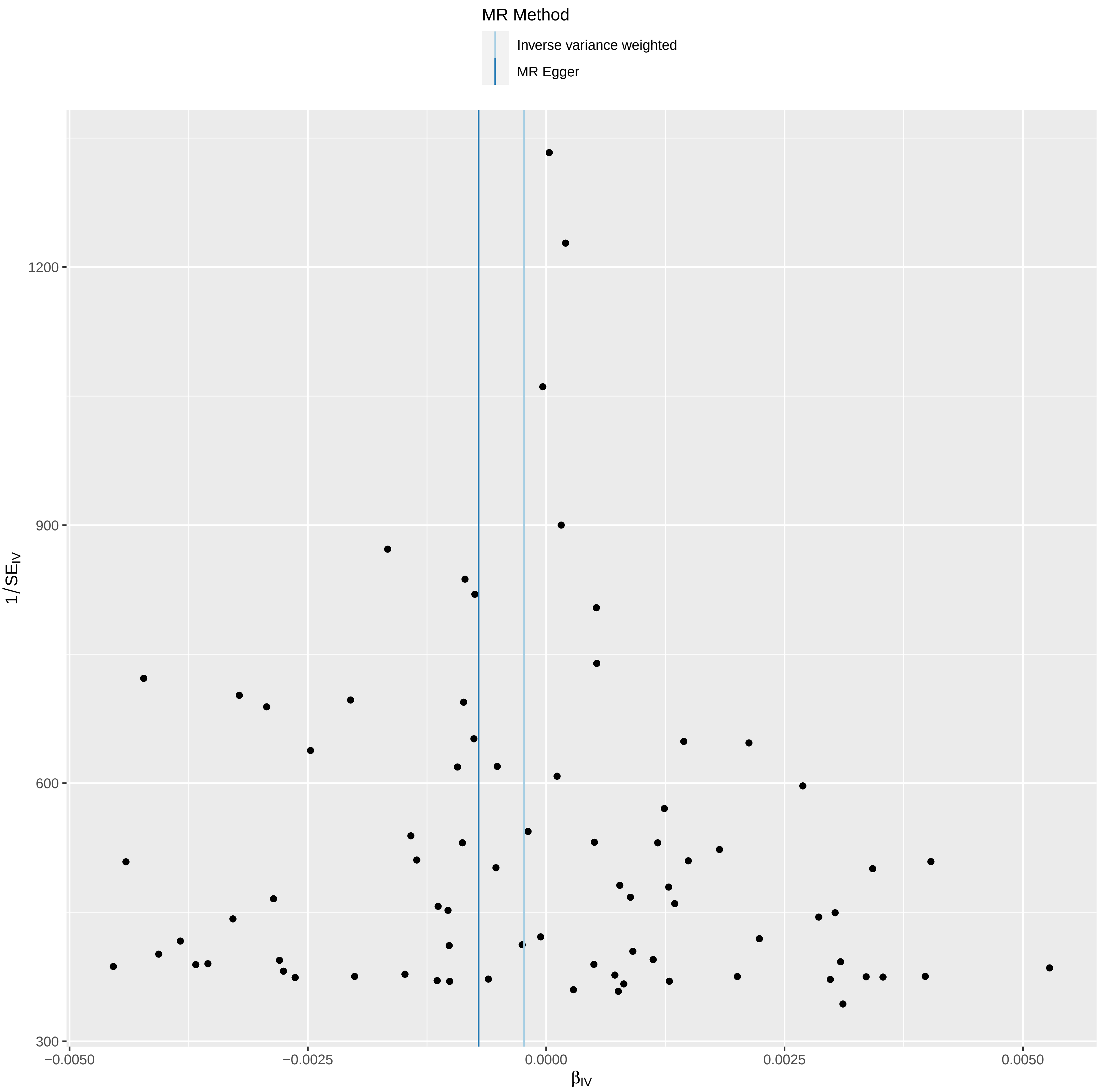
MR Method
Inverse variance weighted
MR Egger
1200
900
600
300
1/SE_IV
−0.0050
−0.0025
0.0000
0.0025
0.0050
β_IV

Figure 9 Leave-one-out analysis, MR effect size and funnel plot for ulcerative colitis on peripheral arterial disease.

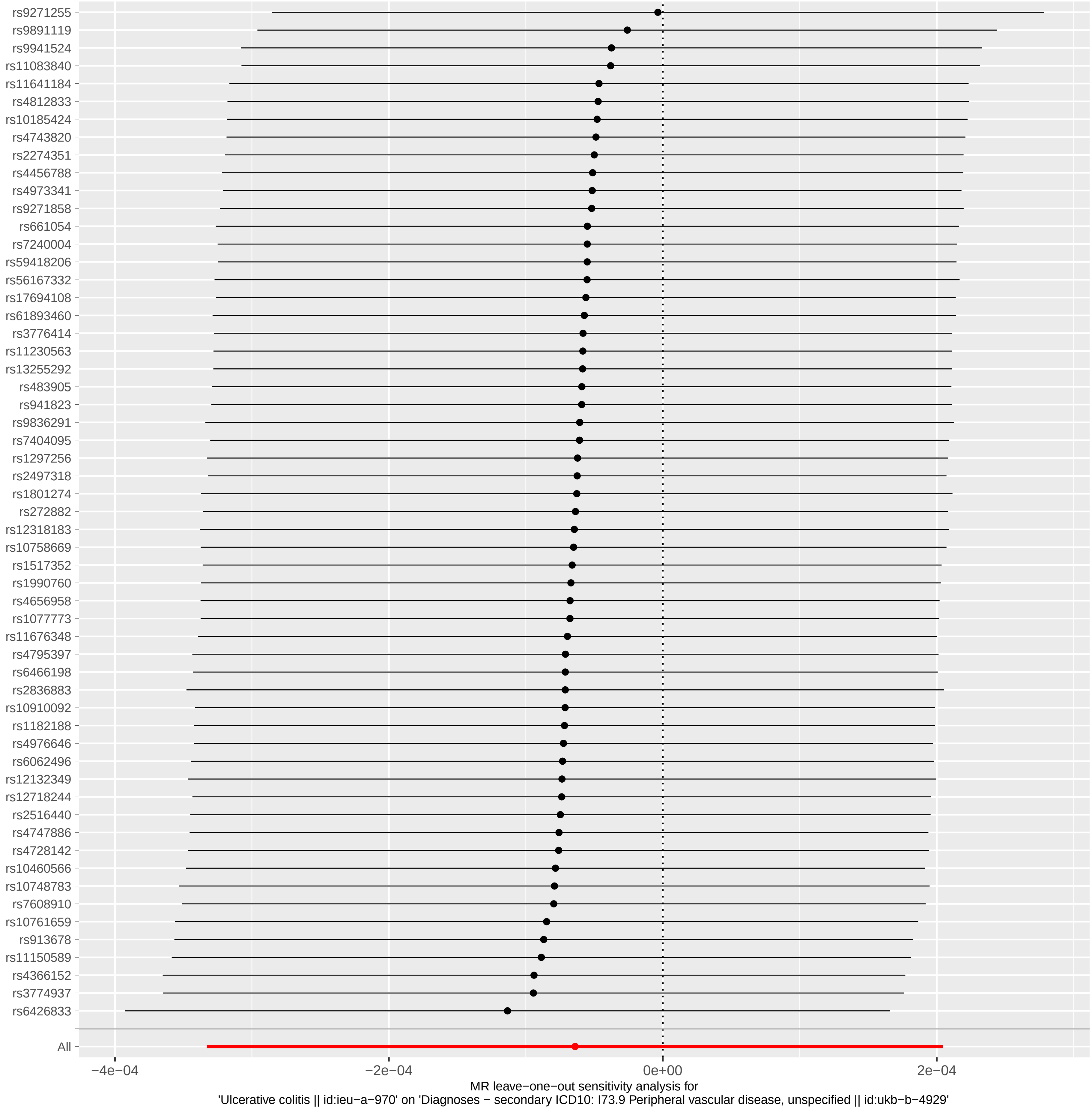

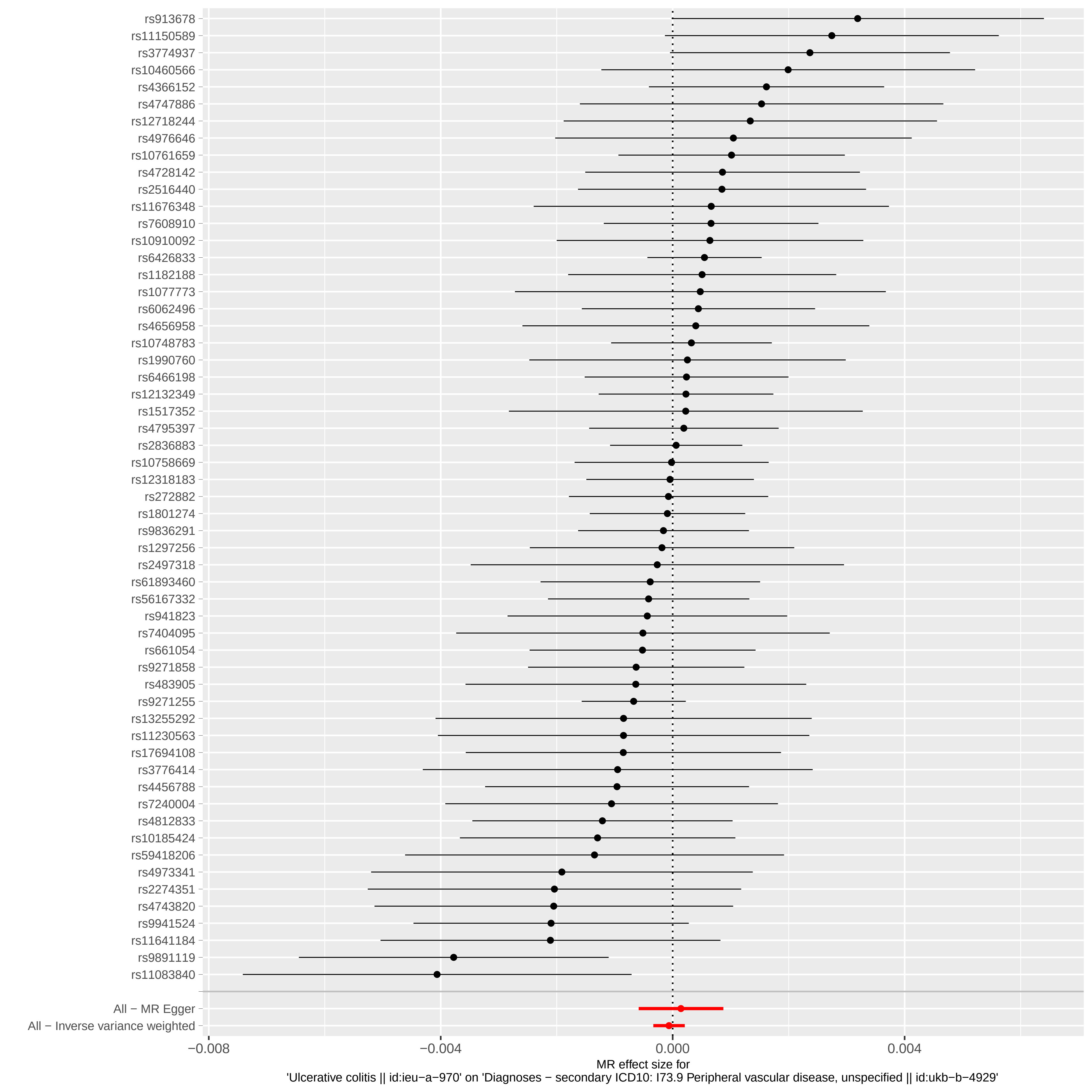
rs913678
rs11150589
rs3774937
rs10460566
rs4366152
rs4747886
rs12718244
rs4976646
rs10761659
rs4728142
rs2516440
rs11676348
rs7608910
rs10910092
rs6426833
rs1182188
rs1077773
rs6062496
rs4656958
rs10748783
rs1990760
rs6466198
rs12132349
rs1517352
rs4795397
rs2836883
rs10758669
rs12318183
rs272882
rs1801274
rs9836291
rs1297256
rs2497318
rs61893460
rs56167332
rs941823
rs7404095
rs661054
rs9271858
rs483905
rs9271255
rs13255292
rs11230563
rs17694108
rs3776414
rs4456788
rs7240004
rs4812833
rs10185424
rs59418206
rs4973341
rs2274351
rs4743820
rs9941524
rs11641184
rs9891119
rs11083840
All − MR Egger
All − Inverse variance weighted
−0.008
−0.004
0.000
0.004
MR effect size for
'Ulcerative colitis || id:ieu−a−970' on 'Diagnoses − secondary ICD10: I73.9 Peripheral vascular disease, unspecified || id:ukb−b−4929'

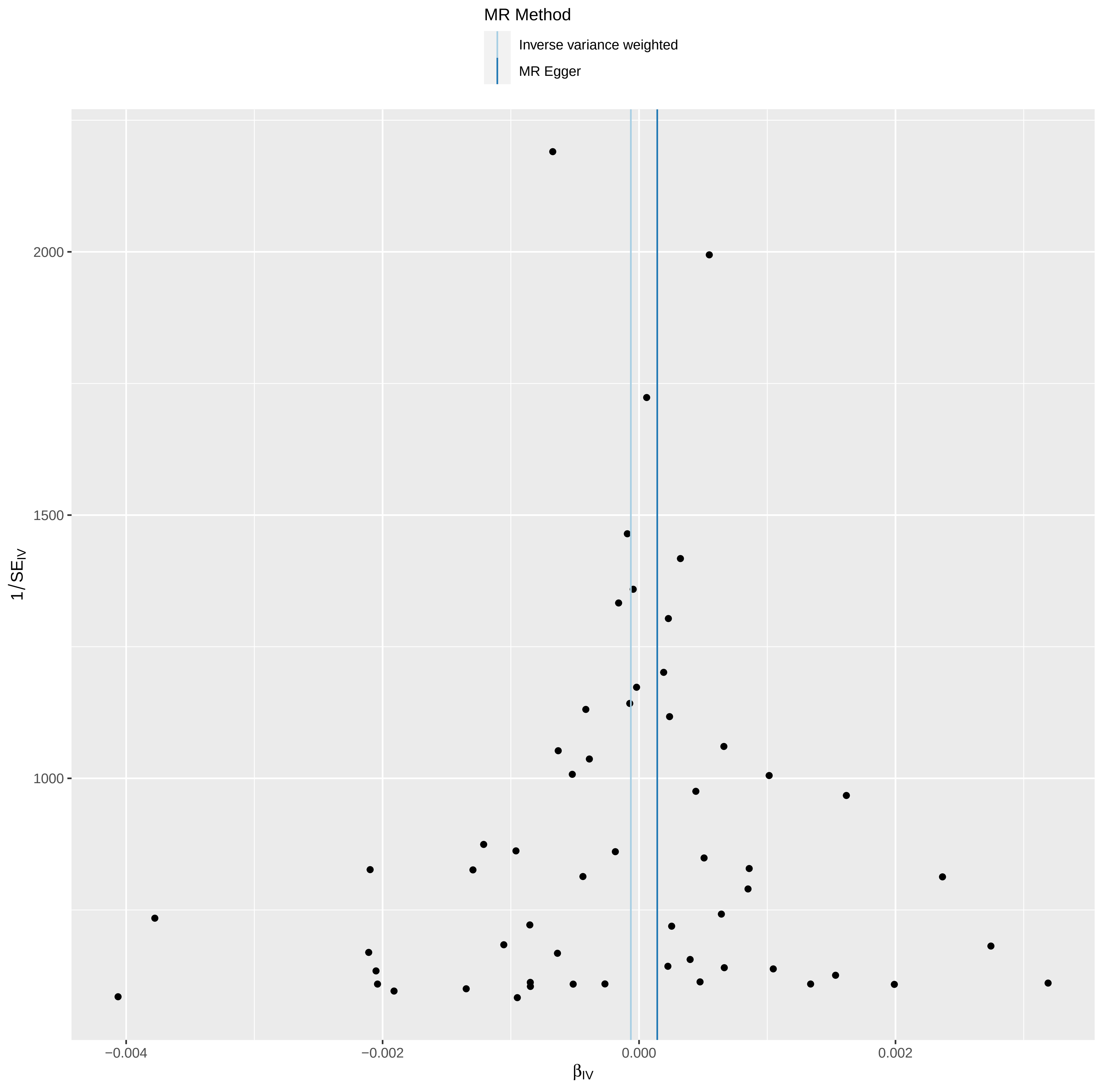
MR Method
Inverse variance weighted
MR Egger
2000
1500
1000
$1/SE_{IV}$
−0.004
−0.002
0.000
0.002
β_{IV}

Figure 10 Leave-one-out analysis, MR effect size and funnel plot for ulcerative colitis on ischemic stroke .

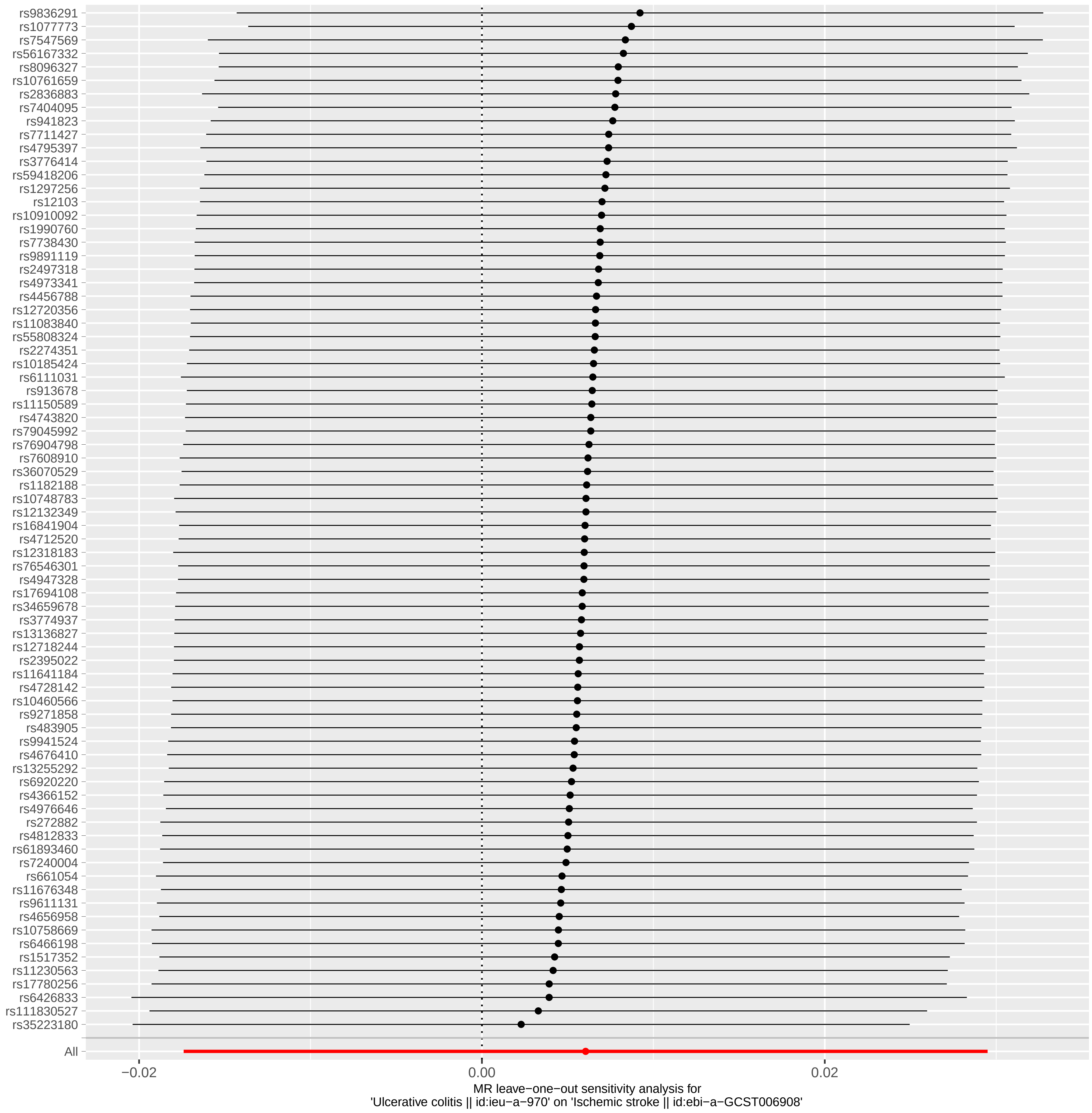

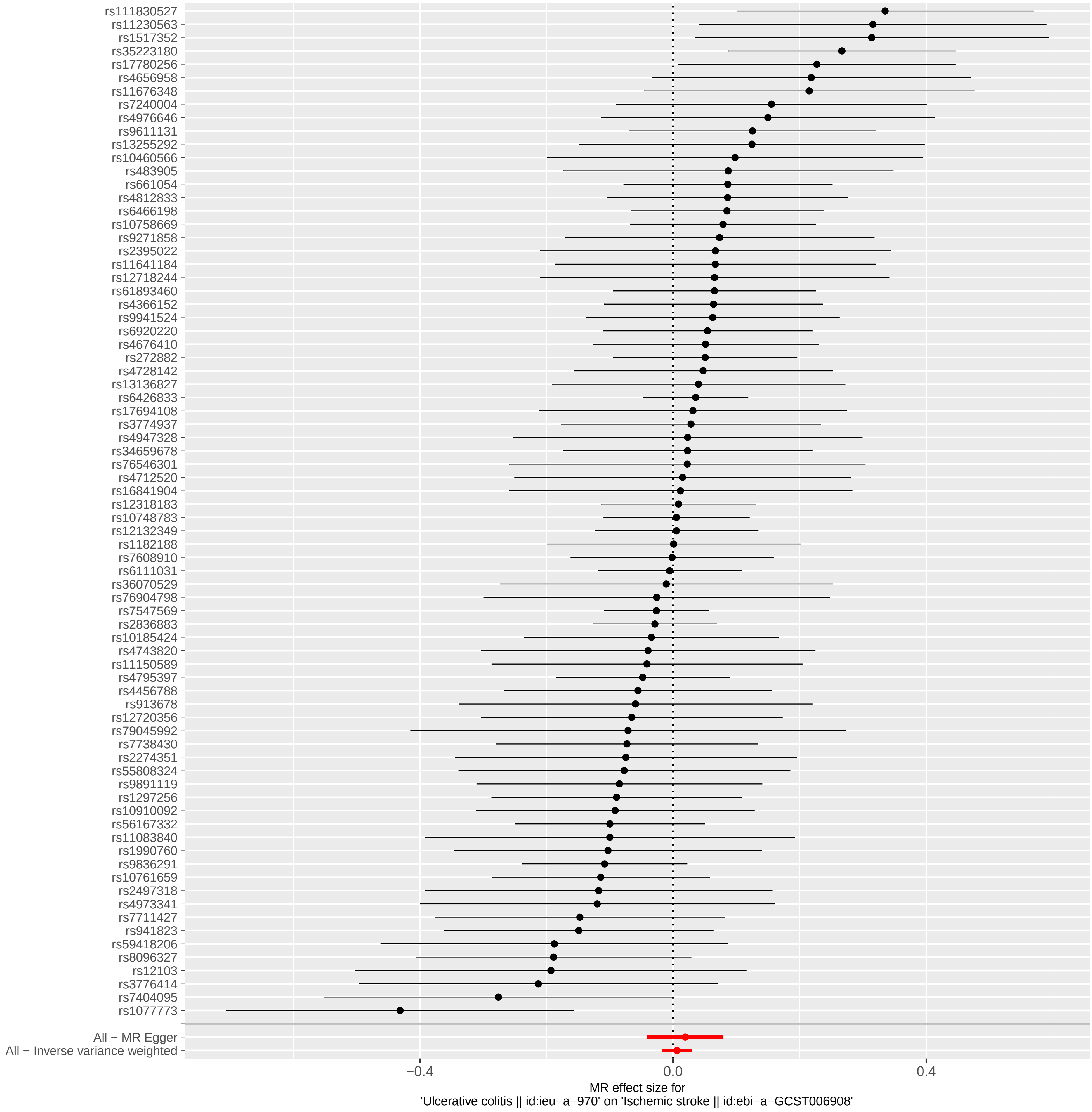

rs111830527
rs11230563
rs1517352
rs35223180
rs17780256
rs4656958
rs11676348
rs7240004
rs4976646
rs9611131
rs13255292
rs10460566
rs483905
rs661054
rs4812833
rs6466198
rs10758669
rs9271858
rs2395022
rs11641184
rs12718244
rs61893460
rs4366152
rs9941524
rs6920220
rs4676410
rs272882
rs4728142
rs13136827
rs6426833
rs17694108
rs3774937
rs4947328
rs34659678
rs76546301
rs4712520
rs16841904
rs12318183
rs10748783
rs12132349
rs1182188
rs7608910
rs6111031
rs36070529
rs76904798
rs7547569
rs2836883
rs10185424
rs4743820
rs11150589
rs4795397
rs4456788
rs913678
rs12720356
rs79045992
rs7738430
rs2274351
rs55808324
rs9891119
rs1297256
rs10910092
rs56167332
rs11083840
rs1990760
rs9836291
rs10761659
rs2497318
rs4973341
rs7711427
rs941823
rs59418206
rs8096327
rs12103
rs3776414
rs7404095
rs1077773
All − MR Egger
All − Inverse variance weighted
−0.4
0.0
0.4
MR effect size for
'Ulcerative colitis || id:ieu−a−970' on 'Ischemic stroke || id:ebi−a−GCST006908'

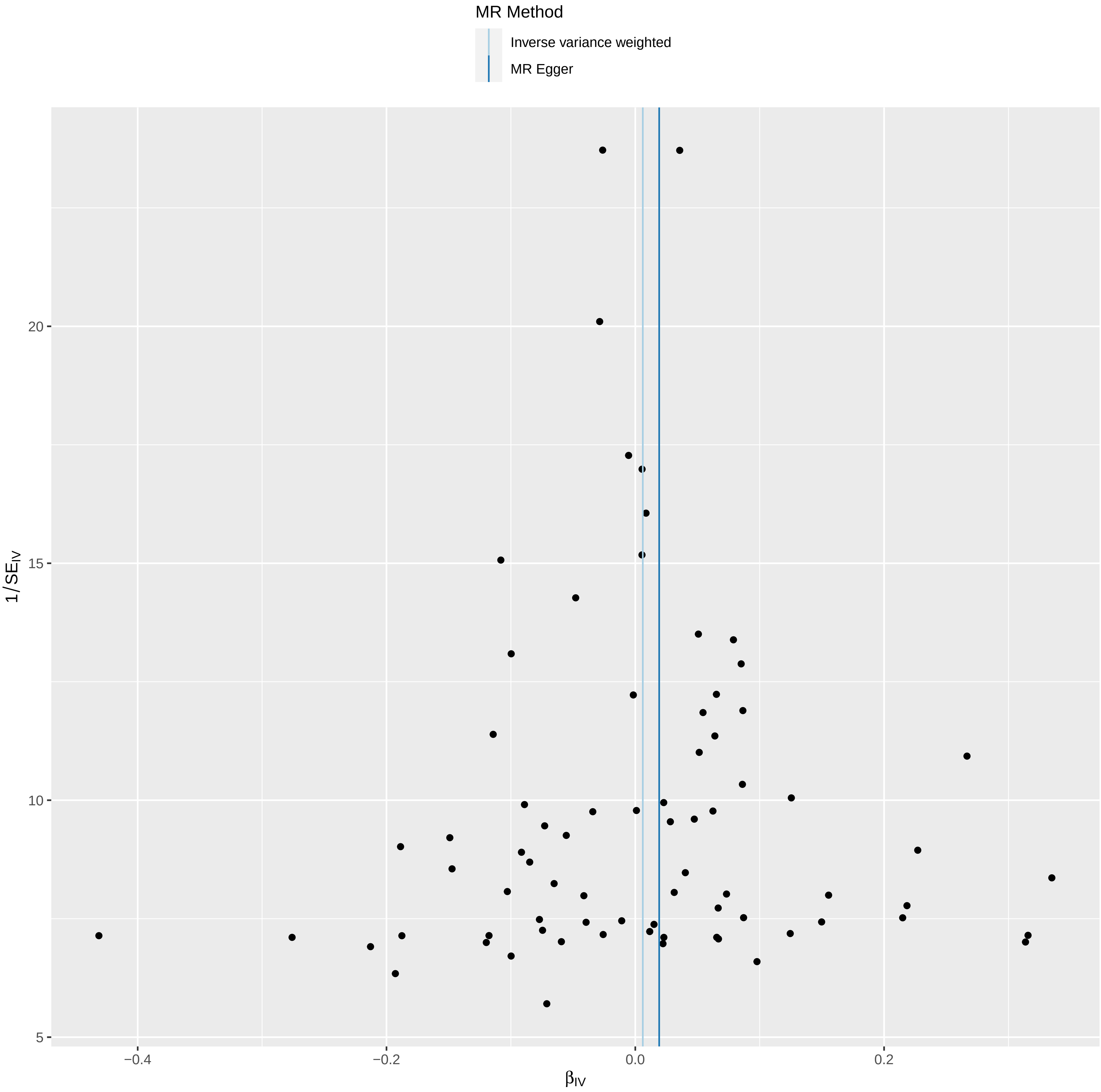
MR Method
Inverse variance weighted
MR Egger
20
15
10
5
1/SE_IV
−0.4
−0.2
0.0
0.2
β_IV

Figure 11 Leave-one-out analysis, MR effect size and funnel plot for ulcerative colitis on intracerebral hemorrhage.

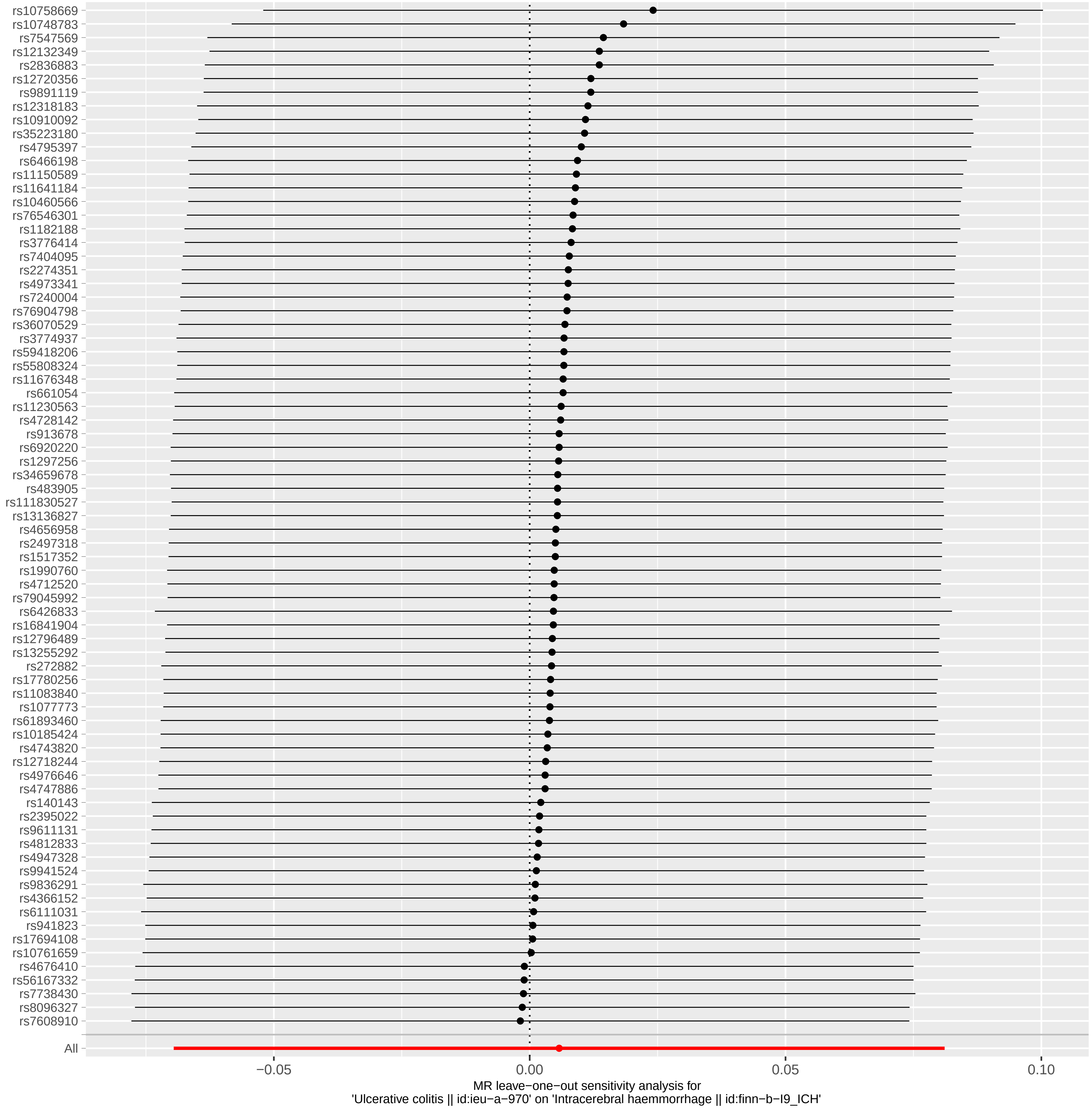

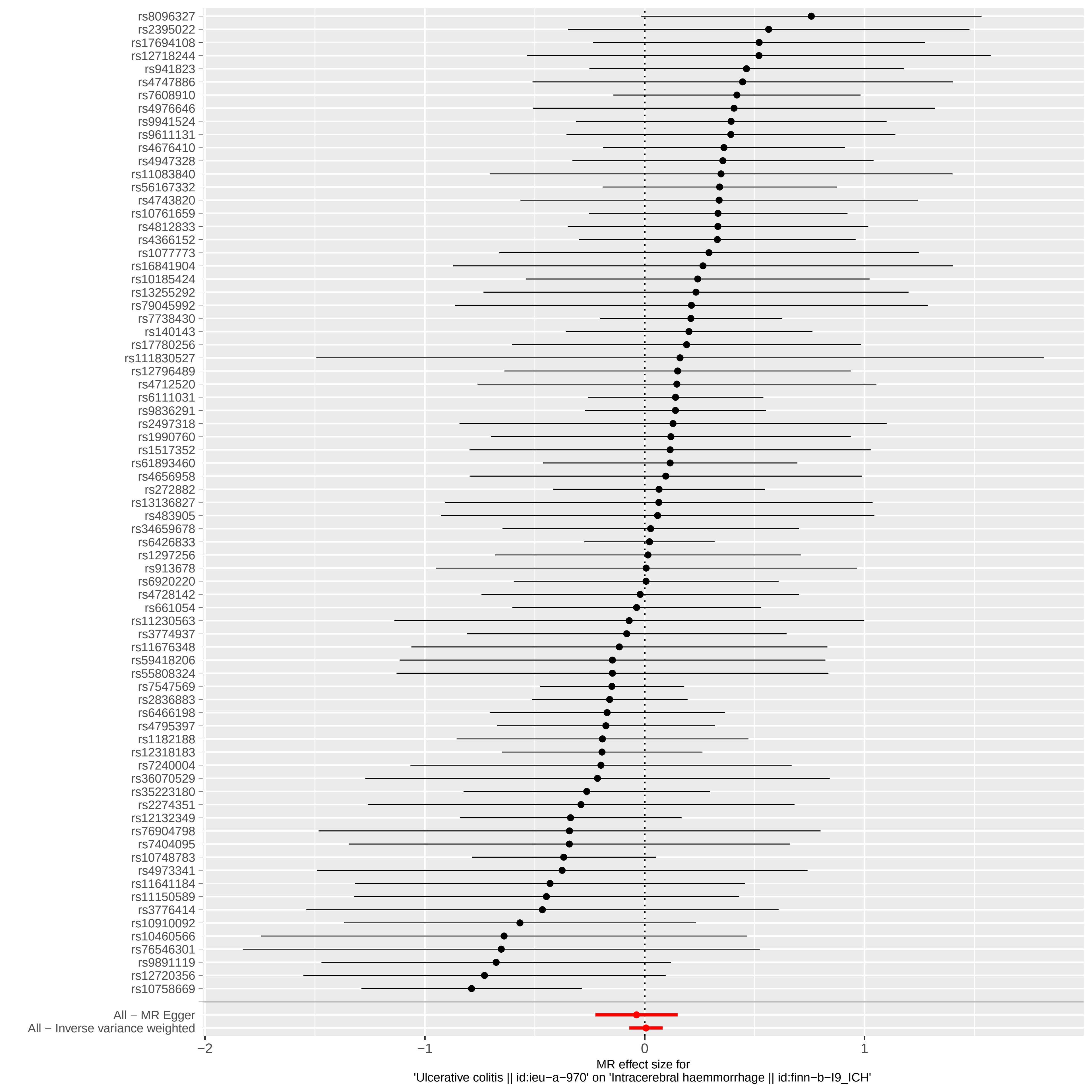

rs8096327
rs2395022
rs17694108
rs12718244
rs941823
rs4747886
rs7608910
rs4976646
rs9941524
rs9611131
rs4676410
rs4947328
rs11083840
rs56167332
rs4743820
rs10761659
rs4812833
rs4366152
rs1077773
rs16841904
rs10185424
rs13255292
rs79045992
rs7738430
rs140143
rs17780256
rs111830527
rs12796489
rs4712520
rs6111031
rs9836291
rs2497318
rs1990760
rs1517352
rs61893460
rs4656958
rs272882
rs13136827
rs483905
rs34659678
rs6426833
rs1297256
rs913678
rs6920220
rs4728142
rs661054
rs11230563
rs3774937
rs11676348
rs59418206
rs55808324
rs7547569
rs2836883
rs6466198
rs4795397
rs1182188
rs12318183
rs7240004
rs36070529
rs35223180
rs2274351
rs12132349
rs76904798
rs7404095
rs10748783
rs4973341
rs11641184
rs11150589
rs3776414
rs10910092
rs10460566
rs76546301
rs9891119
rs12720356
rs10758669
All − MR Egger
All − Inverse variance weighted
−2
−1
0
1
MR effect size for
'Ulcerative colitis || id:ieu−a−970' on 'Intracerebral haemmorrhage || id:finn−b−I9_ICH'

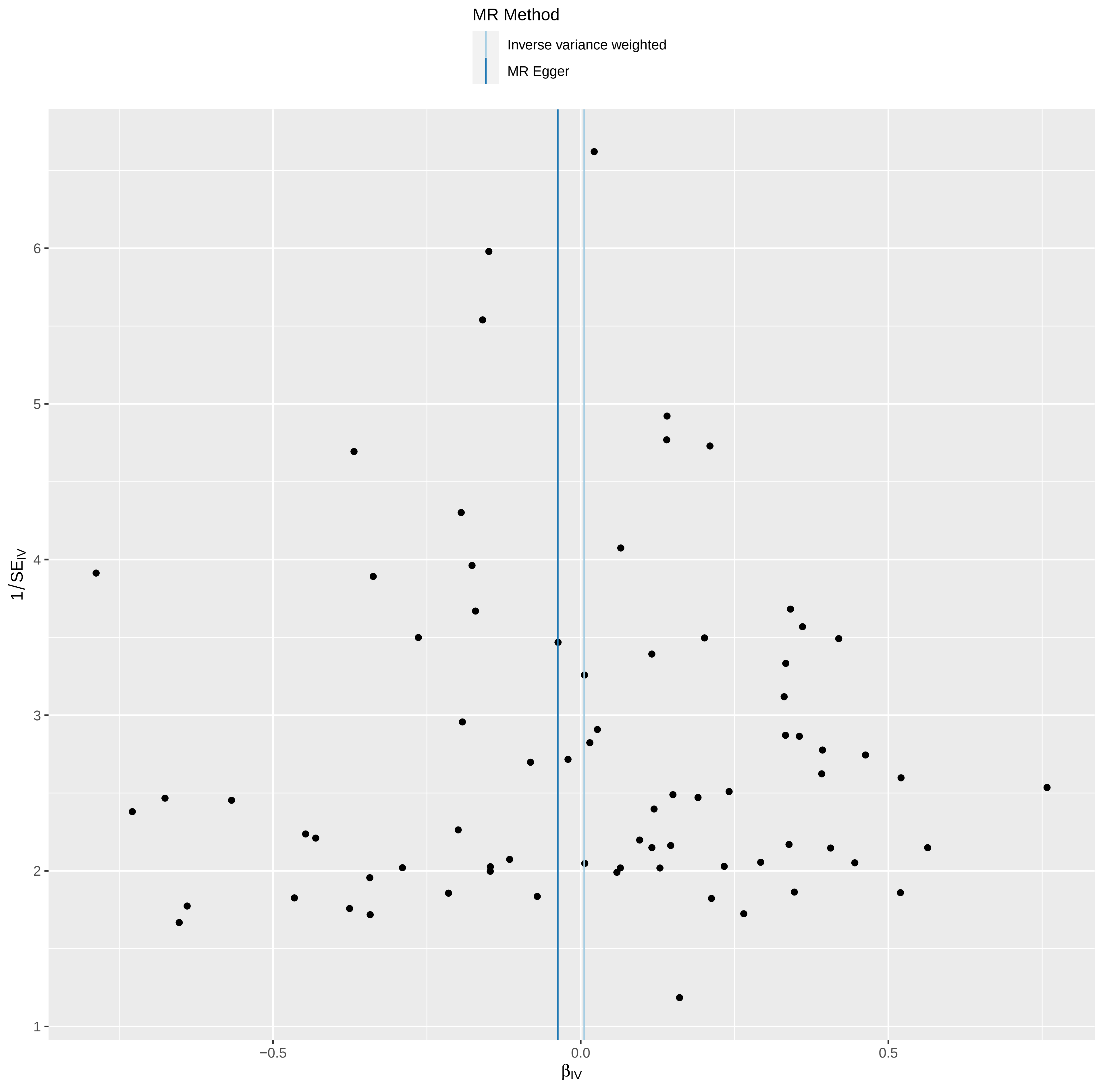

MR Method
Inverse variance weighted
MR Egger
6
5
4
3
2
1
1/SE_IV
−0.5
0.0
0.5
β_IV

Figure 12 Leave-one-out analysis, MR effect size and funnel plot for ulcerative colitis on subarachnoid hemorrhage.

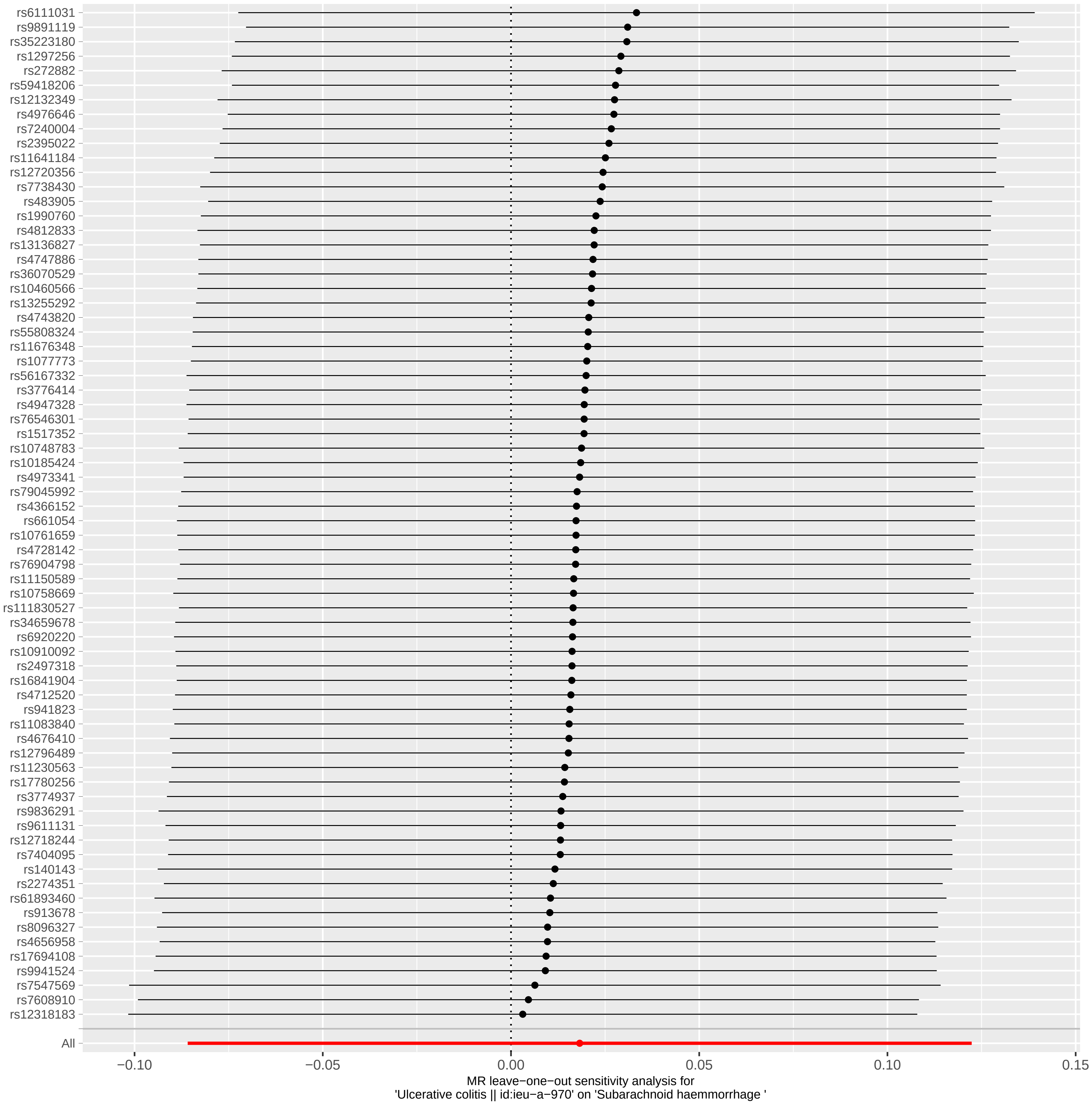

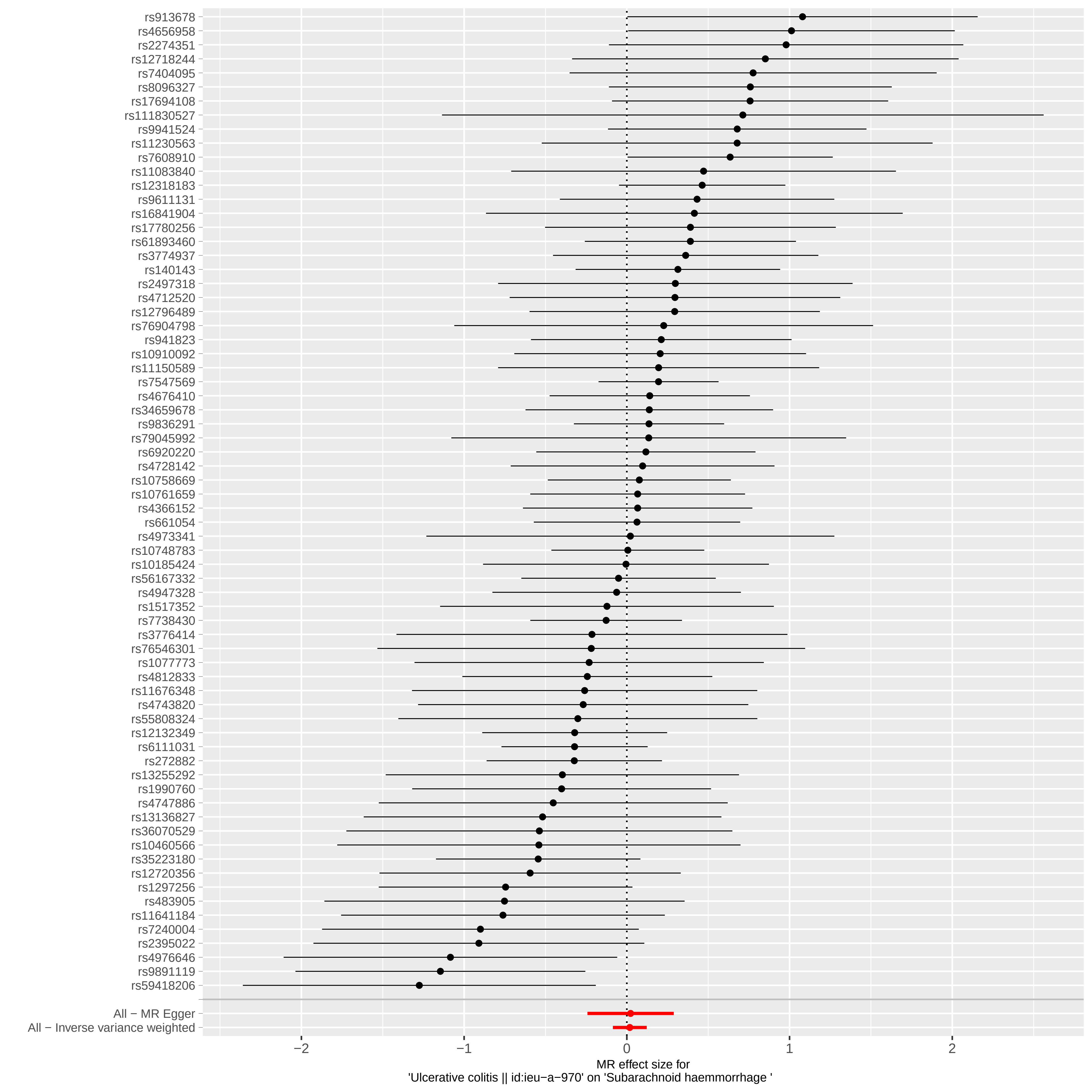

rs913678
rs4656958
rs2274351
rs12718244
rs7404095
rs8096327
rs17694108
rs111830527
rs9941524
rs11230563
rs7608910
rs11083840
rs12318183
rs9611131
rs16841904
rs17780256
rs61893460
rs3774937
rs140143
rs2497318
rs4712520
rs12796489
rs76904798
rs941823
rs10910092
rs11150589
rs7547569
rs4676410
rs34659678
rs9836291
rs79045992
rs6920220
rs4728142
rs10758669
rs10761659
rs4366152
rs661054
rs4973341
rs10748783
rs10185424
rs56167332
rs4947328
rs1517352
rs7738430
rs3776414
rs76546301
rs1077773
rs4812833
rs11676348
rs4743820
rs55808324
rs12132349
rs6111031
rs272882
rs13255292
rs1990760
rs4747886
rs13136827
rs36070529
rs10460566
rs35223180
rs12720356
rs1297256
rs483905
rs11641184
rs7240004
rs2395022
rs4976646
rs9891119
rs59418206
All − MR Egger
All − Inverse variance weighted
−2
−1
0
1
2
MR effect size for
'Ulcerative colitis || id:ieu−a−970' on 'Subarachnoid haemmorrhage '

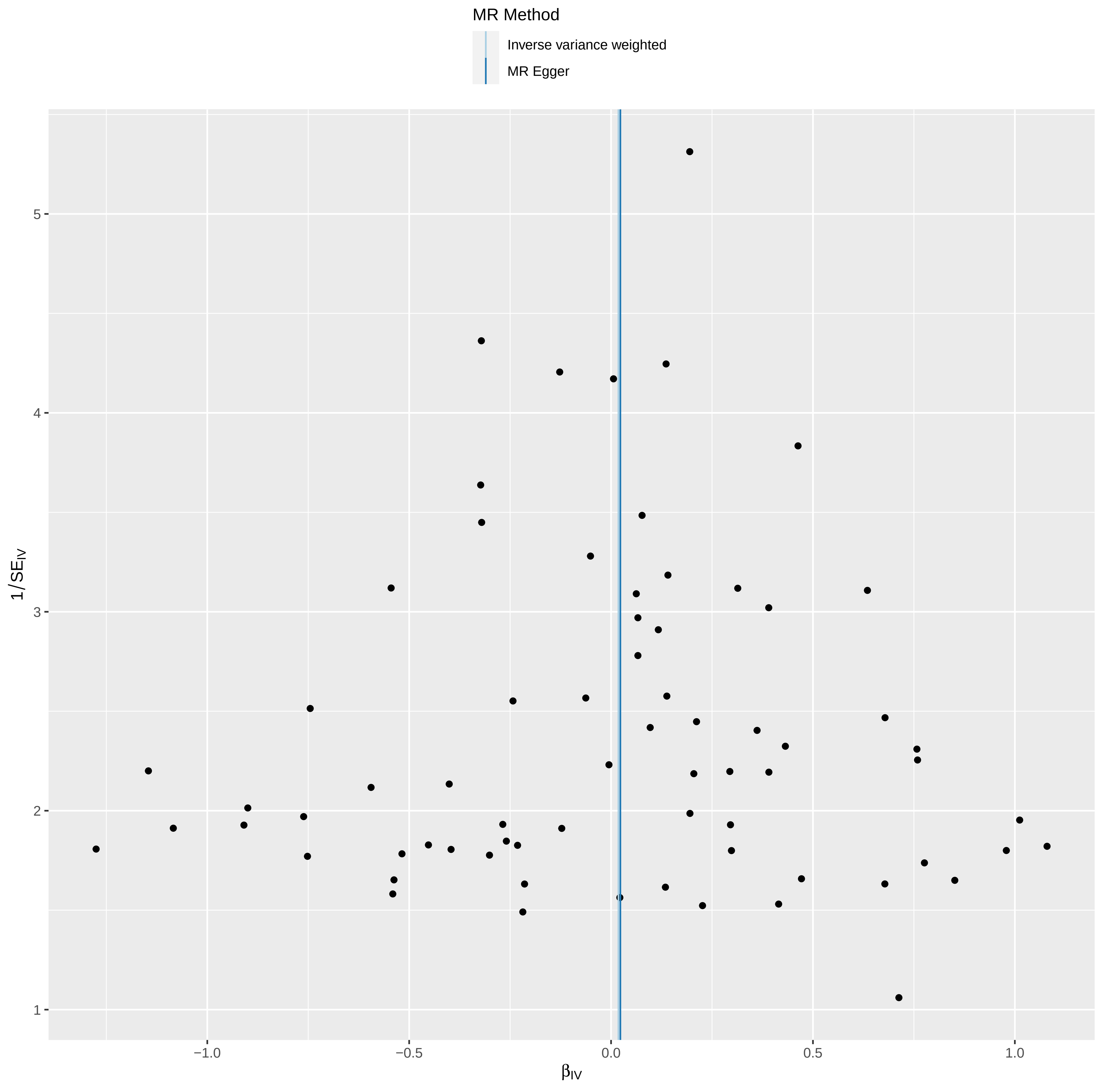

MR Method
Inverse variance weighted
MR Egger
1/SE_IV
5
4
3
2
1
−1.0
−0.5
0.0
0.5
1.0
β_IV

Figure 13 Leave-one-out analysis, MR effect size and funnel plot for ulcerative colitis on cerebral infarction.

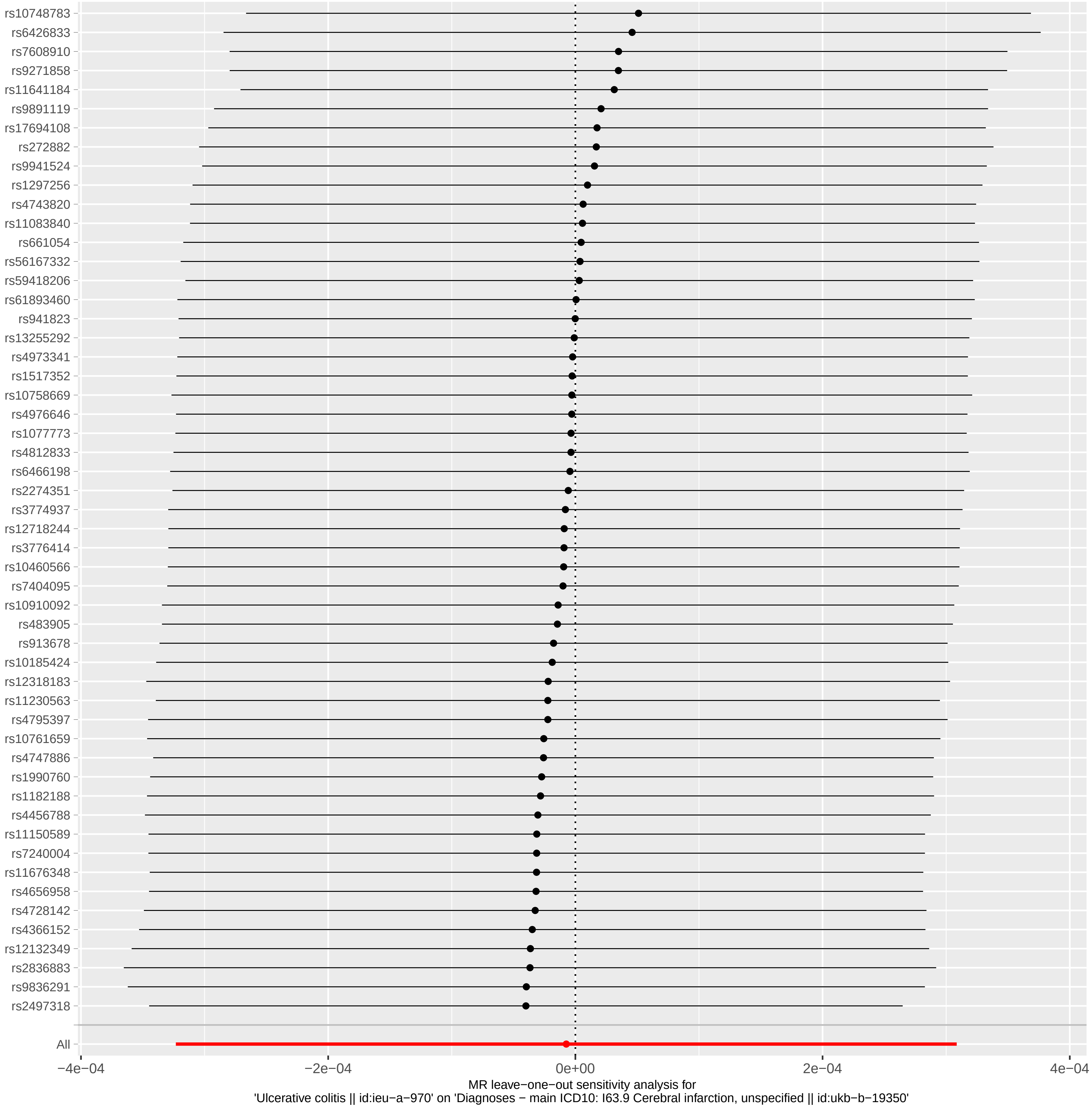

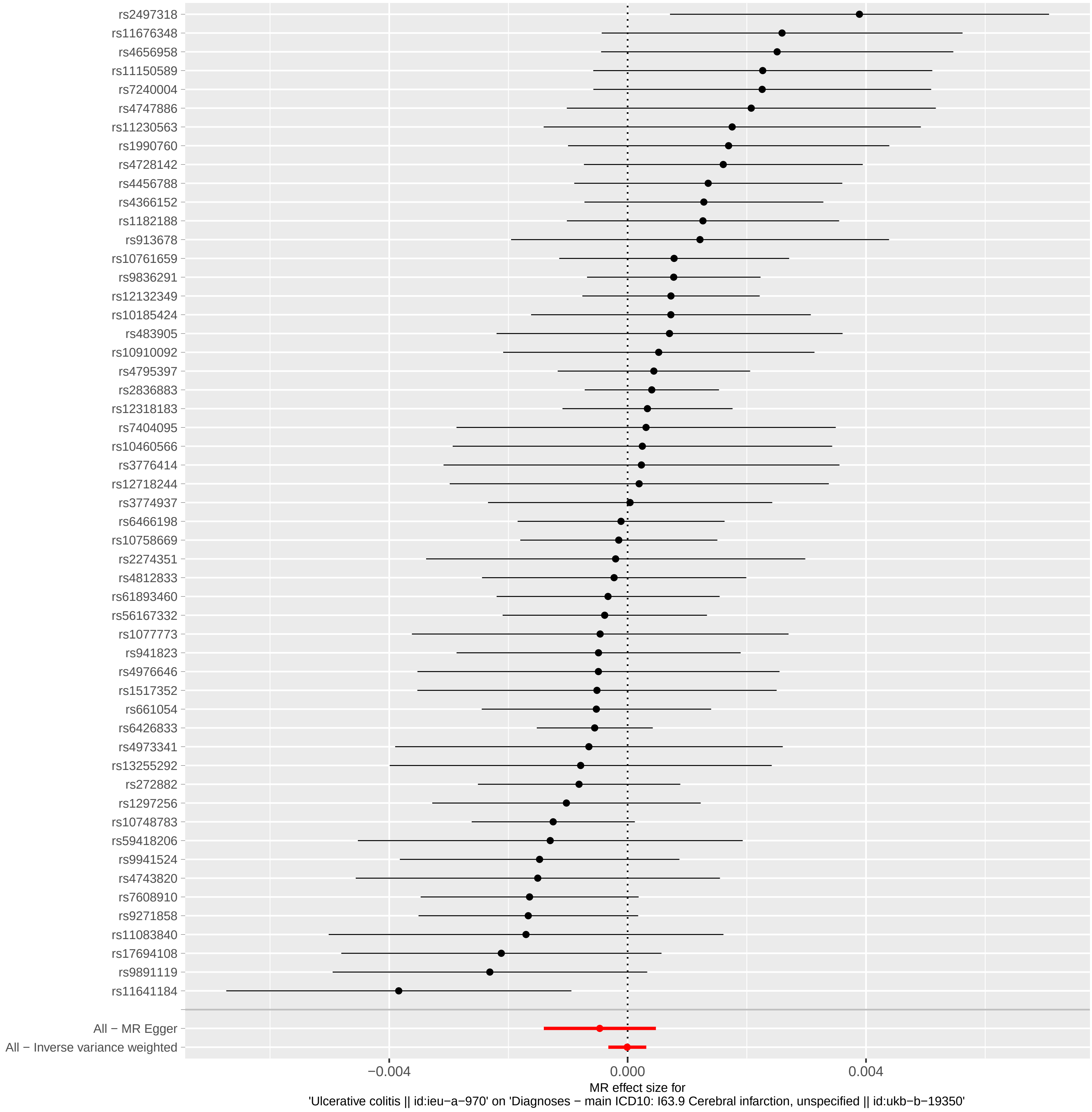

rs2497318
rs11676348
rs4656958
rs11150589
rs7240004
rs4747886
rs11230563
rs1990760
rs4728142
rs4456788
rs4366152
rs1182188
rs913678
rs10761659
rs9836291
rs12132349
rs10185424
rs483905
rs10910092
rs4795397
rs2836883
rs12318183
rs7404095
rs10460566
rs3776414
rs12718244
rs3774937
rs6466198
rs10758669
rs2274351
rs4812833
rs61893460
rs56167332
rs1077773
rs941823
rs4976646
rs1517352
rs661054
rs6426833
rs4973341
rs13255292
rs272882
rs1297256
rs10748783
rs59418206
rs9941524
rs4743820
rs7608910
rs9271858
rs11083840
rs17694108
rs9891119
rs11641184
All − MR Egger
All − Inverse variance weighted
−0.004
0.000
0.004
MR effect size for
'Ulcerative colitis || id:ieu−a−970' on 'Diagnoses − main ICD10: I63.9 Cerebral infarction, unspecified || id:ukb−b−19350'

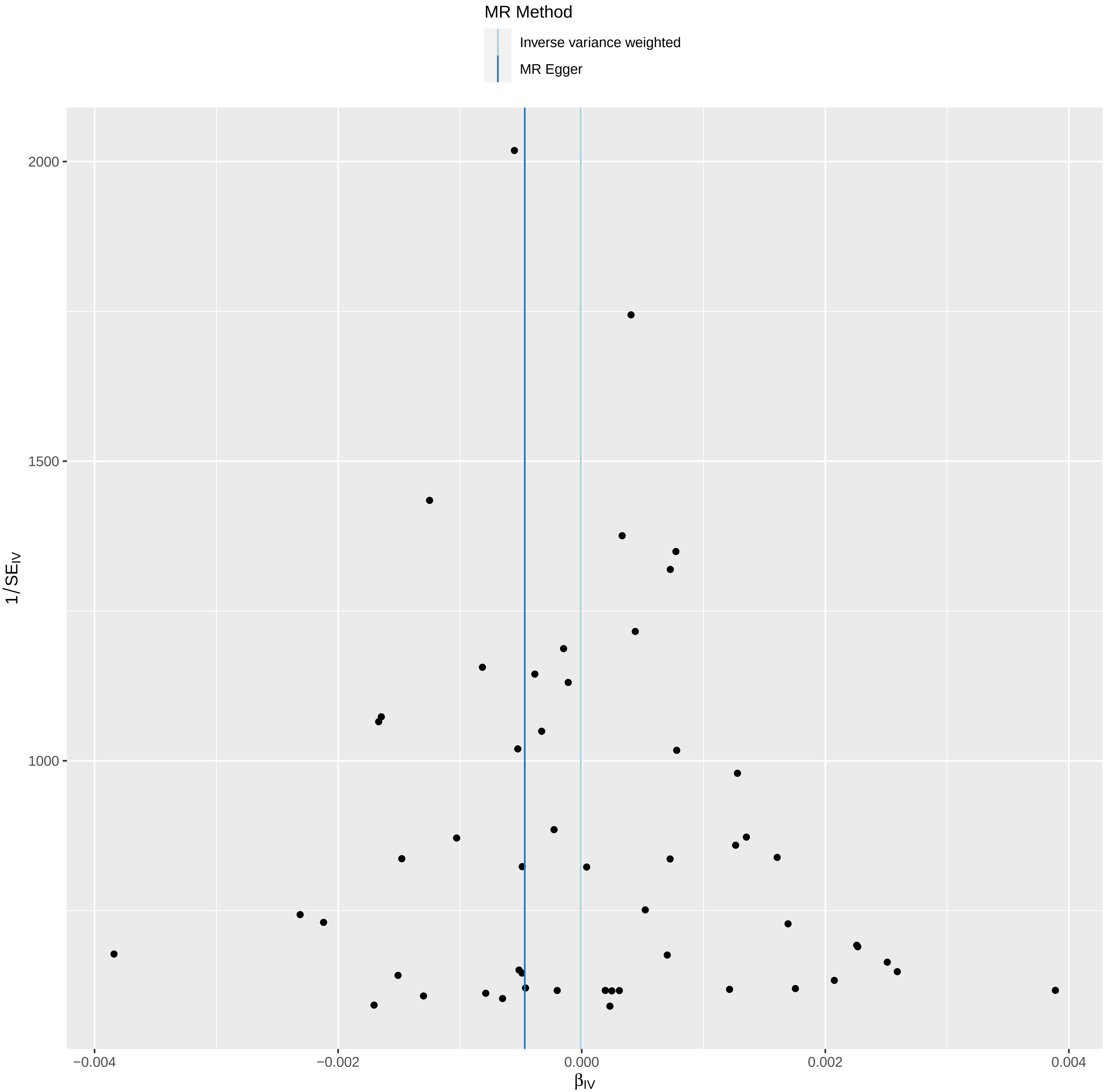

MR Method
Inverse variance weighted
MR Egger
2000
1500
1000
1/SE_IV
−0.004
−0.002
0.000
0.002
0.004
β_IV

Figure 14 Leave-one-out analysis, MR effect size and funnel plot for ulcerative colitis on atherosclerosis.

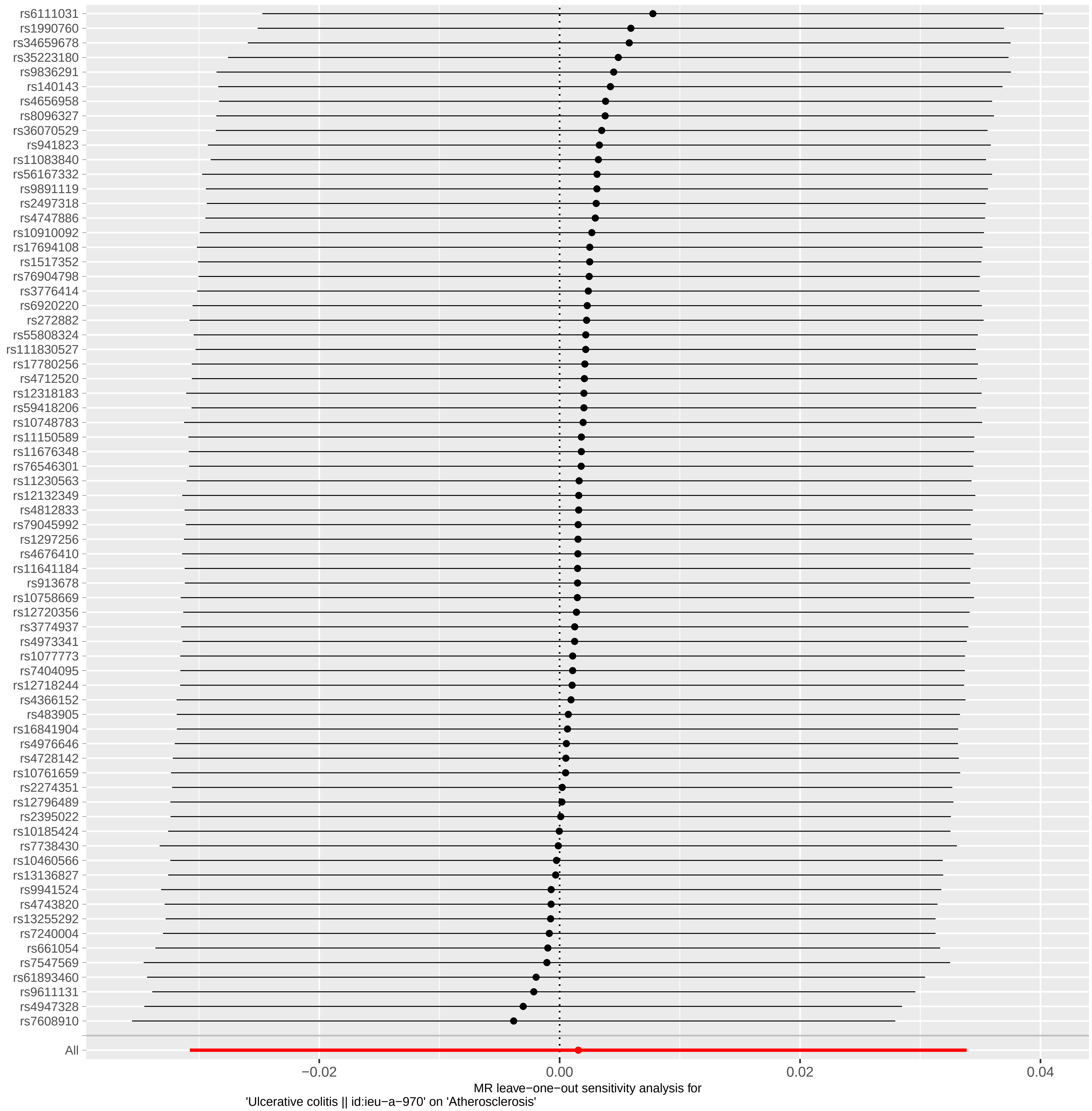

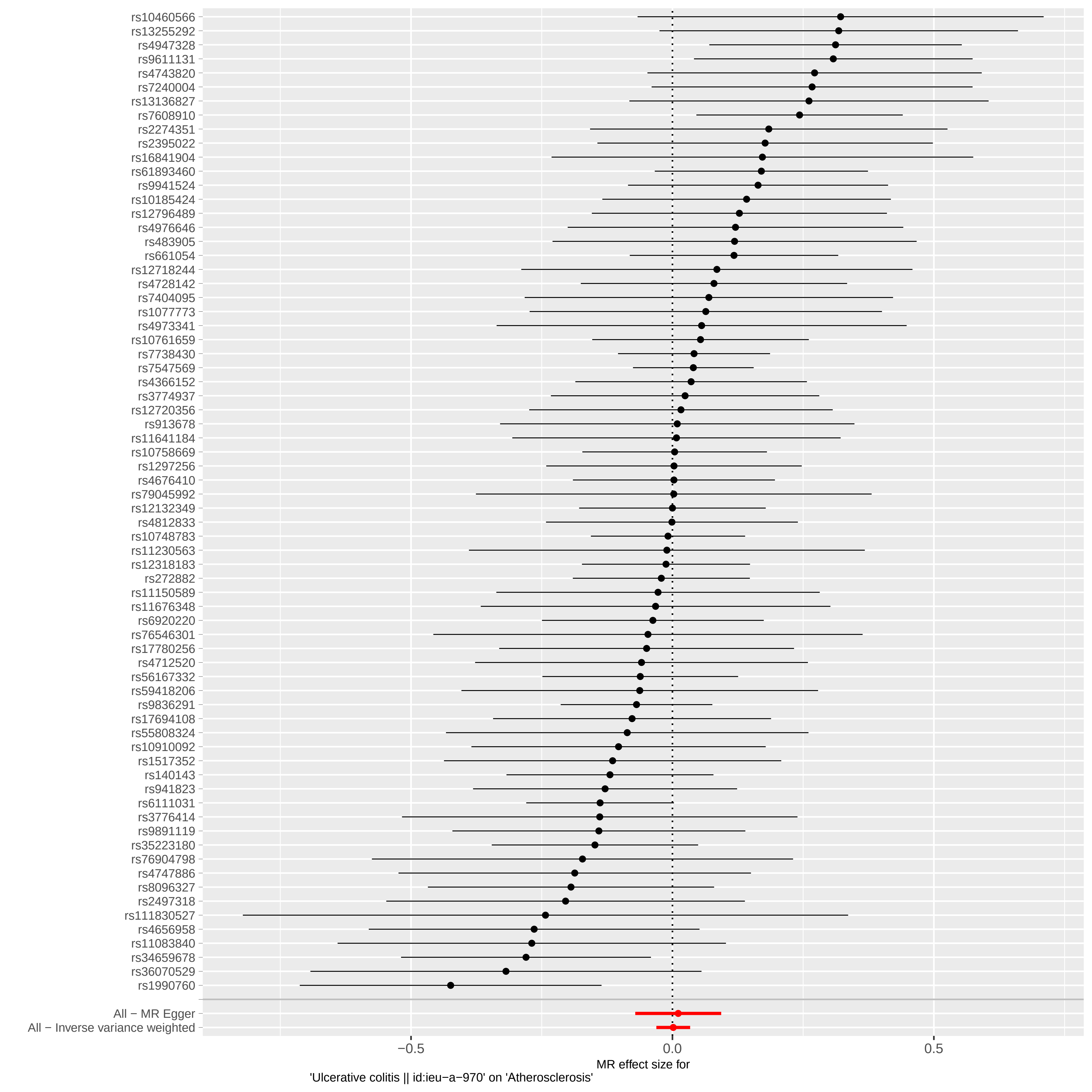

rs10460566
rs13255292
rs4947328
rs9611131
rs4743820
rs7240004
rs13136827
rs7608910
rs2274351
rs2395022
rs16841904
rs61893460
rs9941524
rs10185424
rs12796489
rs4976646
rs483905
rs661054
rs12718244
rs4728142
rs7404095
rs1077773
rs4973341
rs10761659
rs7738430
rs7547569
rs4366152
rs3774937
rs12720356
rs913678
rs11641184
rs10758669
rs1297256
rs4676410
rs79045992
rs12132349
rs4812833
rs10748783
rs11230563
rs12318183
rs272882
rs11150589
rs11676348
rs6920220
rs76546301
rs17780256
rs4712520
rs56167332
rs59418206
rs9836291
rs17694108
rs55808324
rs10910092
rs1517352
rs140143
rs941823
rs6111031
rs3776414
rs9891119
rs35223180
rs76904798
rs4747886
rs8096327
rs2497318
rs111830527
rs4656958
rs11083840
rs34659678
rs36070529
rs1990760
All − MR Egger
All − Inverse variance weighted
−0.5
0.0
0.5
MR effect size for
'Ulcerative colitis || id:ieu−a−970' on 'Atherosclerosis'

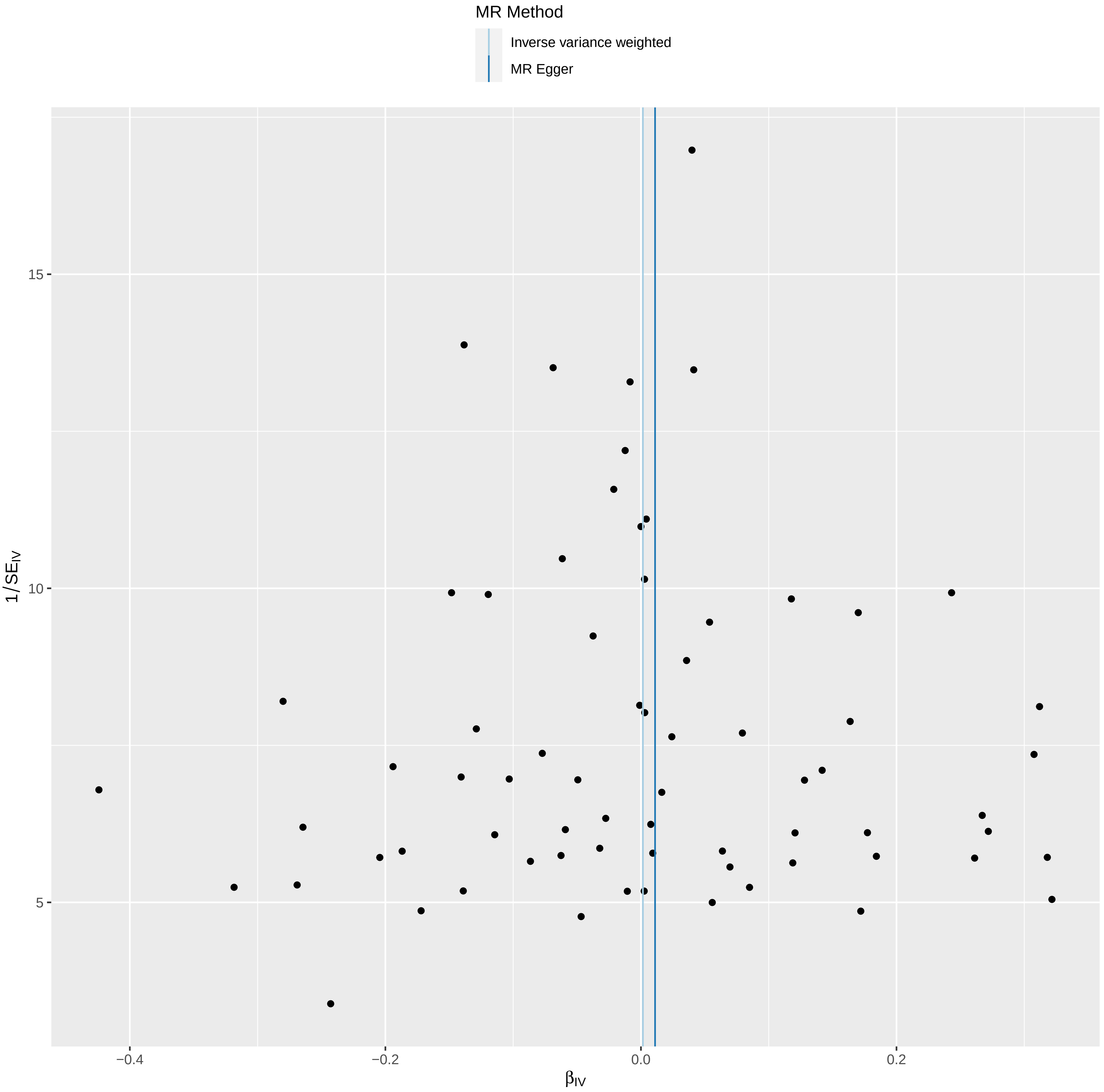
MR Method
Inverse variance weighted
MR Egger
15
10
5
1/SE_IV
−0.4
−0.2
0.0
0.2
β_IV
