# Supplementary material for: Inflammatory bowel disease and cardiovascular disease: A two-sample Mendelian randomization analysis
Source: Front Cardiovasc Med. 2022 Sep 2;9:927120. doi: 10.3389/fcvm.2022.927120 (PMC9478388; doi:10.3389/fcvm.2022.927120)
Supplement: Supplementary material 1 — Instrumental SNPs from ulcerative colitis and Crohn’s disease GWASs. [file Data_Sheet_1.ZIP › Supplementary material 4.pdf]

Figure 1 Leave-one-out analysis, MR effect size and funnel plot for Crohn's disease on coronary heart disease.

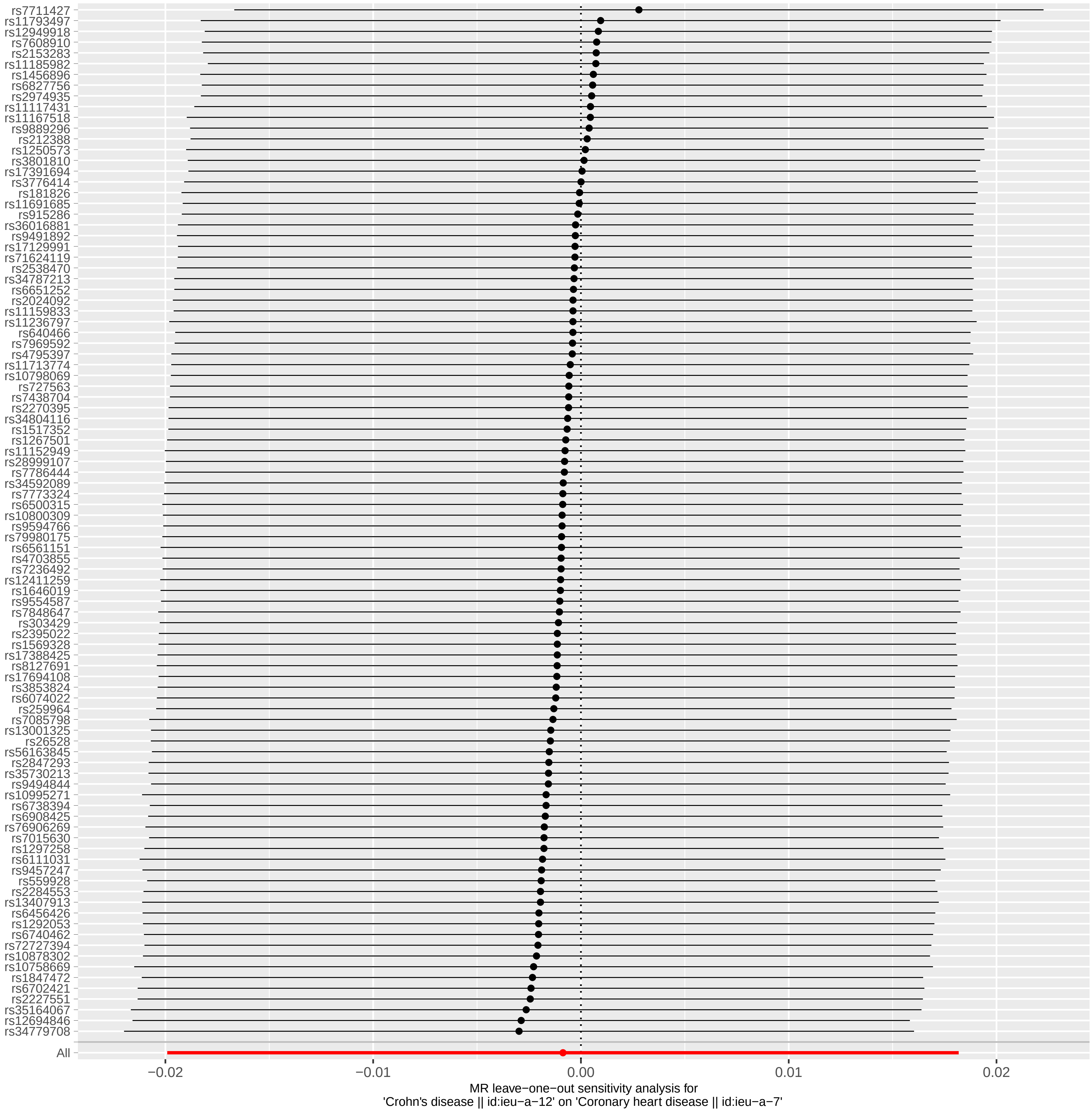

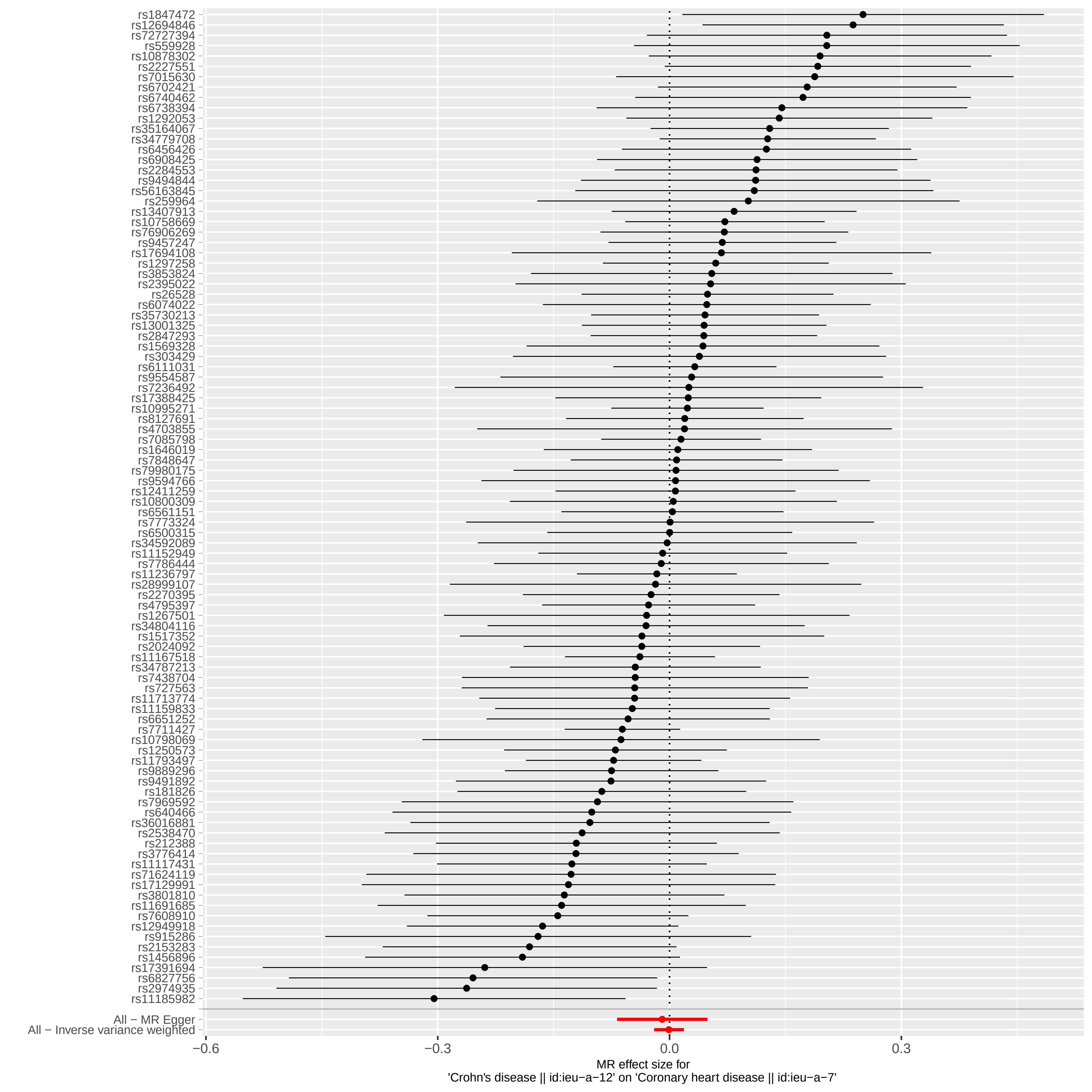
rs1847472
rs12694846
rs72727394
rs559928
rs10878302
rs2227551
rs7015630
rs6702421
rs6740462
rs6738394
rs1292053
rs35164067
rs34779708
rs6456426
rs6908425
rs2284553
rs9494844
rs56163845
rs259964
rs13407913
rs10758669
rs76906269
rs9457247
rs17694108
rs1297258
rs3853824
rs2395022
rs26528
rs6074022
rs35730213
rs13001325
rs2847293
rs1569328
rs303429
rs6111031
rs9554587
rs7236492
rs17388425
rs10995271
rs8127691
rs4703855
rs7085798
rs1646019
rs7848647
rs79980175
rs9594766
rs12411259
rs10800309
rs6561151
rs7773324
rs6500315
rs34592089
rs11152949
rs7786444
rs11236797
rs28999107
rs2270395
rs4795397
rs1267501
rs34804116
rs1517352
rs2024092
rs11167518
rs34787213
rs7438704
rs727563
rs11713774
rs11159833
rs6651252
rs7711427
rs10798069
rs1250573
rs11793497
rs9889296
rs9491892
rs181826
rs7969592
rs640466
rs36016881
rs2538470
rs212388
rs3776414
rs11117431
rs71624119
rs17129991
rs3801810
rs11691685
rs7608910
rs12949918
rs915286
rs2153283
rs1456896
rs17391694
rs6827756
rs2974935
rs11185982
All − MR Egger
All − Inverse variance weighted
−0.6
−0.3
0.0
0.3
MR effect size for
'Crohn's disease || id:ieu−a−12' on 'Coronary heart disease || id:ieu−a−7'

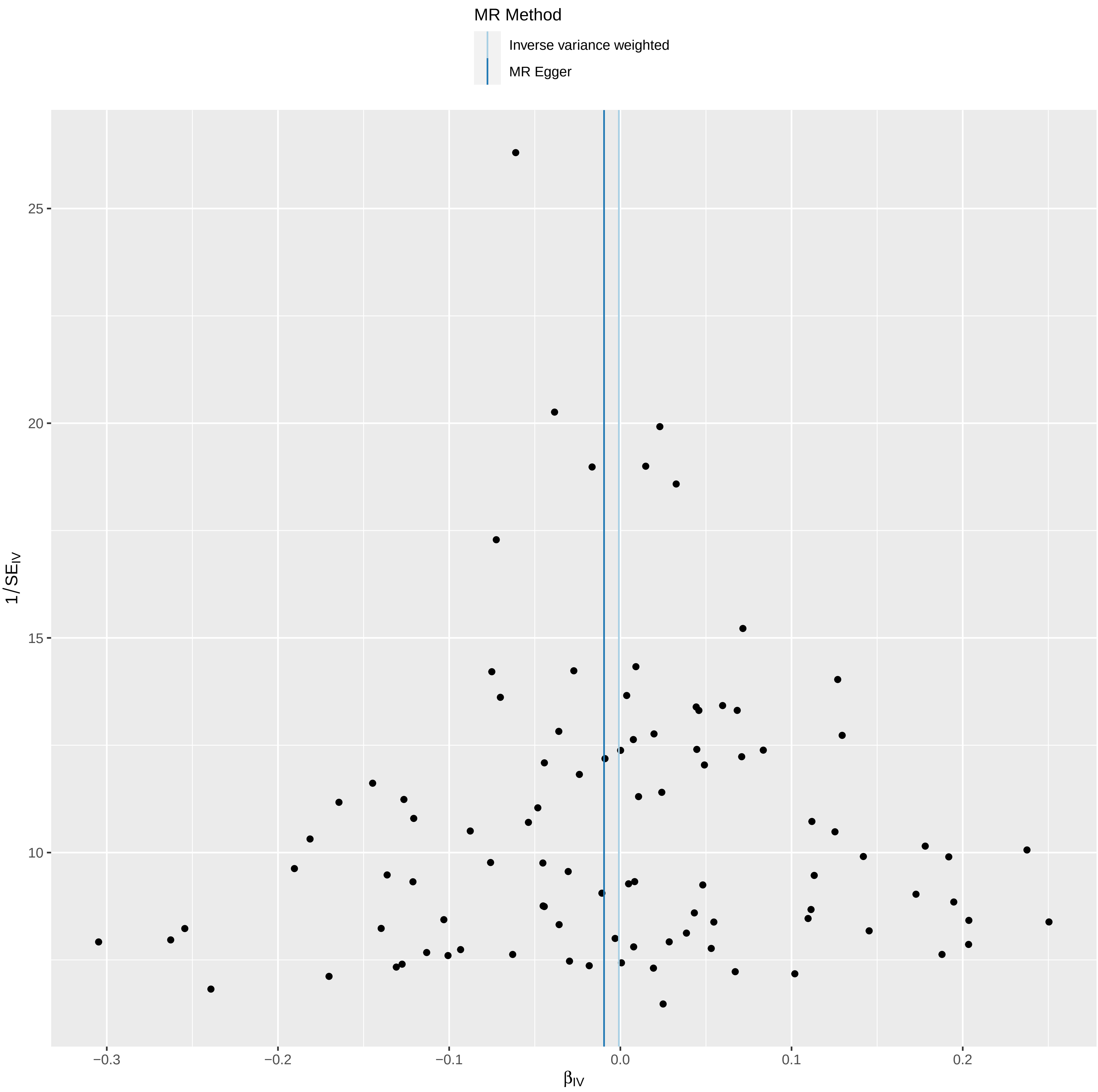
MR Method
Inverse variance weighted
MR Egger
1/SE_IV
β_IV
25
20
15
10
−0.3
−0.2
−0.1
0.0
0.1
0.2

Figure 2 Leave-one-out analysis, MR effect size and funnel plot for Crohn's disease on coronary atherosclerosis.

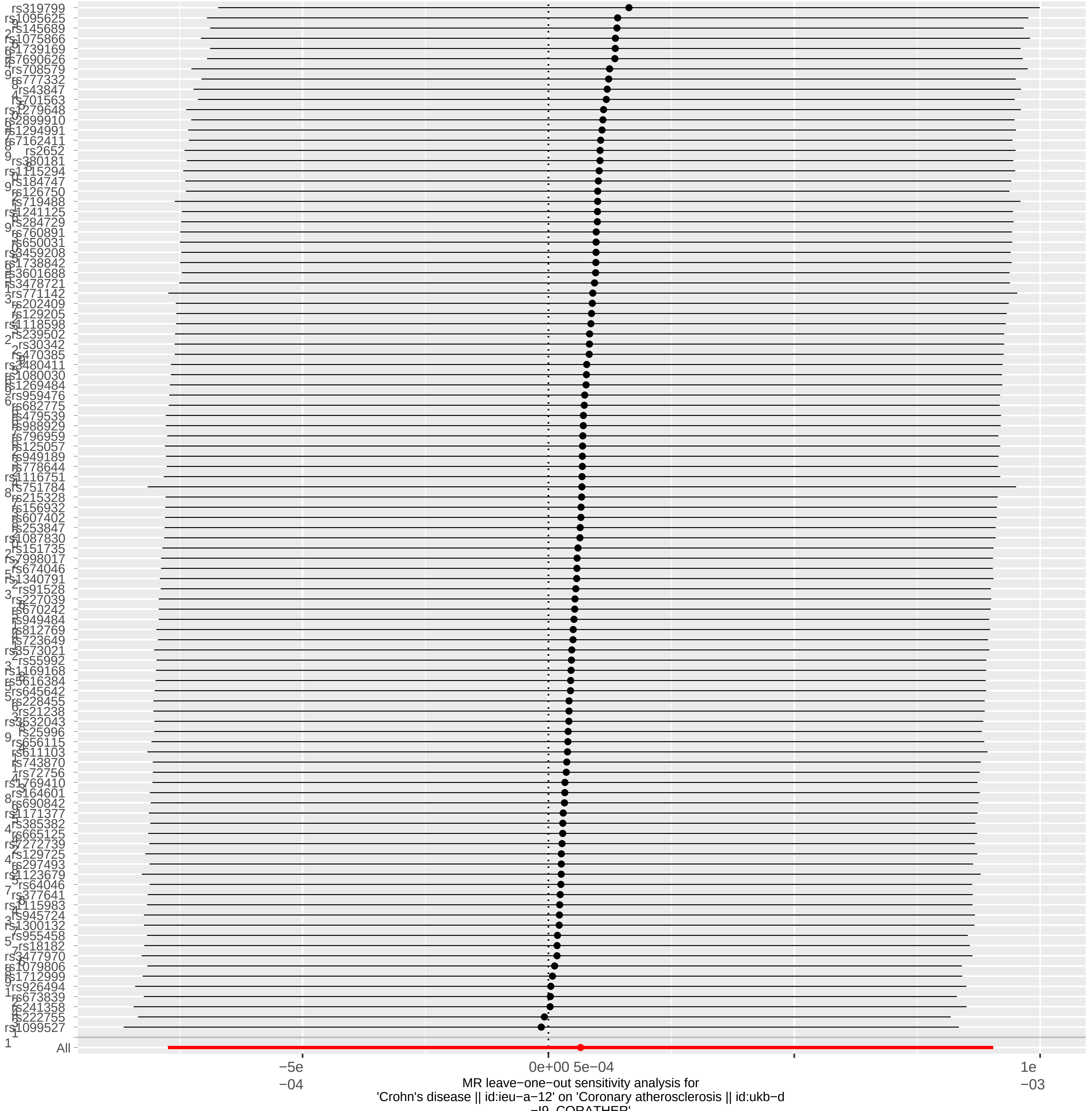

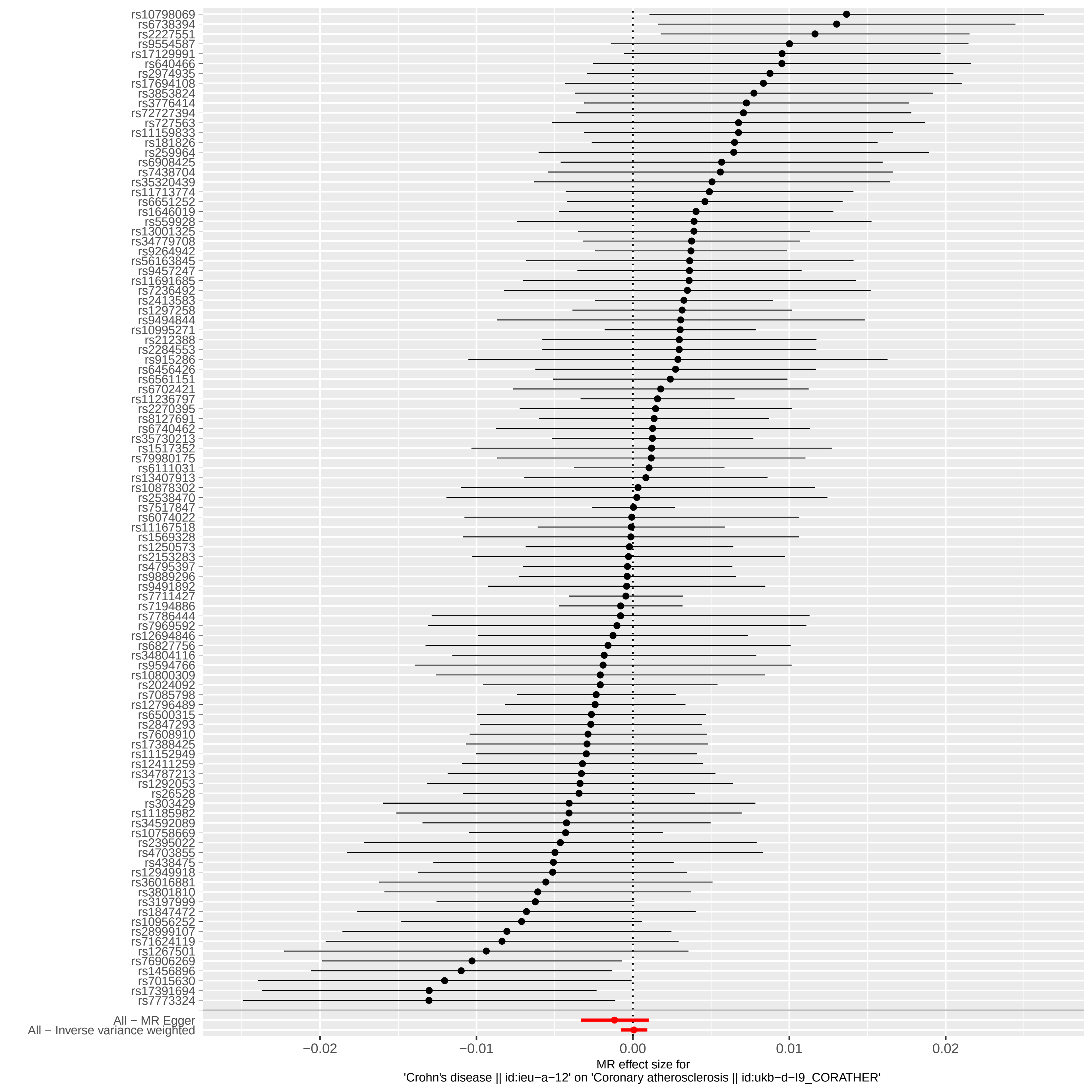
rs10798069
rs6738394
rs2227551
rs9554587
rs17129991
rs640466
rs2974935
rs17694108
rs3853824
rs3776414
rs72727394
rs727563
rs11159833
rs181826
rs259964
rs6908425
rs7438704
rs35320439
rs11713774
rs6651252
rs1646019
rs559928
rs13001325
rs34779708
rs9264942
rs56163845
rs9457247
rs11691685
rs7236492
rs2413583
rs1297258
rs9494844
rs10995271
rs212388
rs2284553
rs915286
rs6456426
rs6561151
rs6702421
rs11236797
rs2270395
rs8127691
rs6740462
rs35730213
rs1517352
rs79980175
rs6111031
rs13407913
rs10878302
rs2538470
rs7517847
rs6074022
rs11167518
rs1569328
rs1250573
rs2153283
rs4795397
rs9889296
rs9491892
rs7711427
rs7194886
rs7786444
rs7969592
rs12694846
rs6827756
rs34804116
rs9594766
rs10800309
rs2024092
rs7085798
rs12796489
rs6500315
rs2847293
rs7608910
rs17388425
rs11152949
rs12411259
rs34787213
rs1292053
rs26528
rs303429
rs11185982
rs34592089
rs10758669
rs2395022
rs4703855
rs438475
rs12949918
rs36016881
rs3801810
rs3197999
rs1847472
rs10956252
rs28999107
rs71624119
rs1267501
rs76906269
rs1456896
rs7015630
rs17391694
rs7773324
All − MR Egger
All − Inverse variance weighted
−0.02
−0.01
0.00
0.01
0.02
MR effect size for
'Crohn's disease || id:ieu−a−12' on 'Coronary atherosclerosis || id:ukb−d−I9_CORATHER'

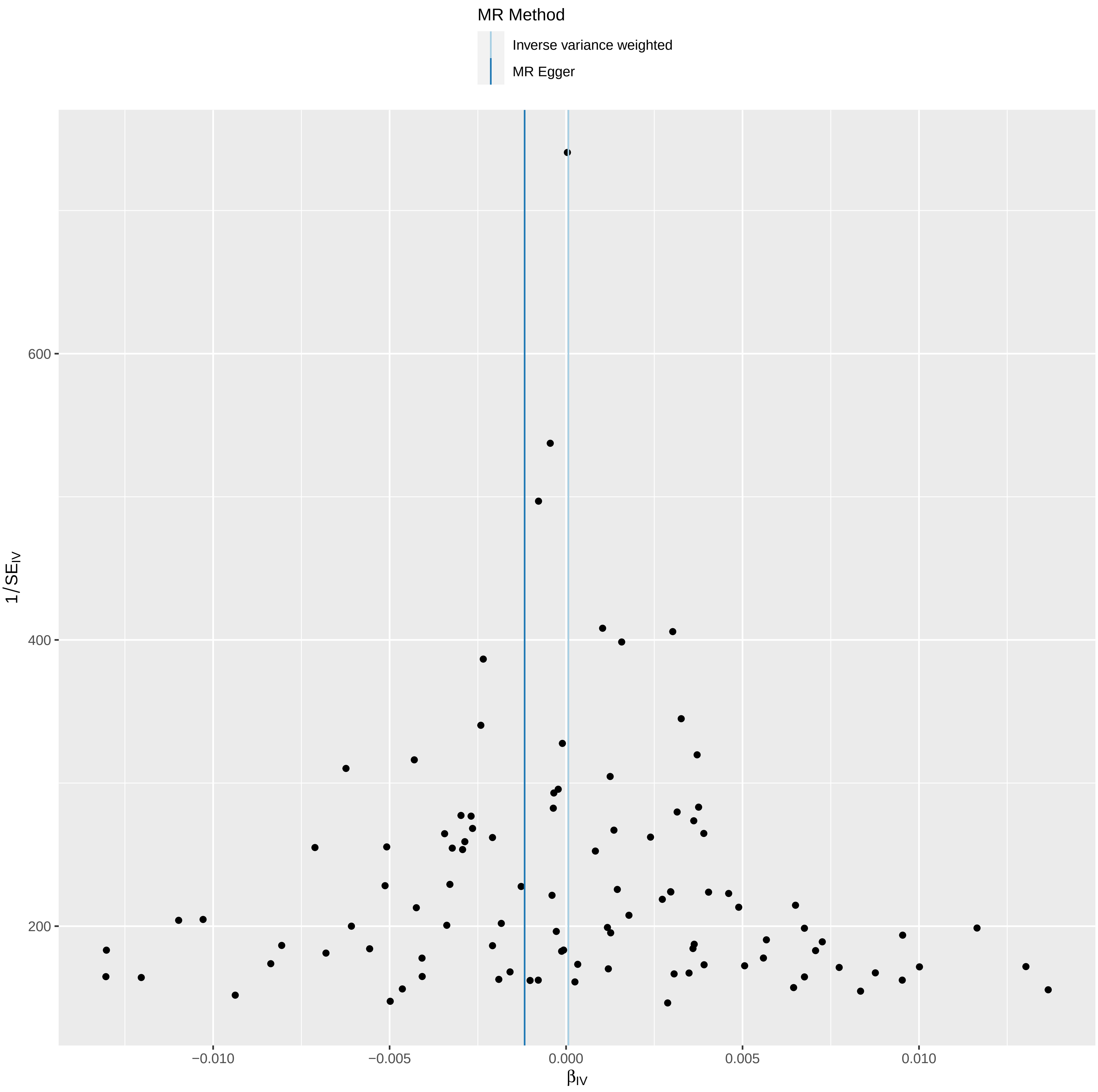
MR Method
Inverse variance weighted
MR Egger
1/SE_IV
600
400
200
−0.010
−0.005
0.000
0.005
0.010
β_IV

Figure 3 Leave-one-out analysis, MR effect size and funnel plot for Crohn's disease on heart failure.

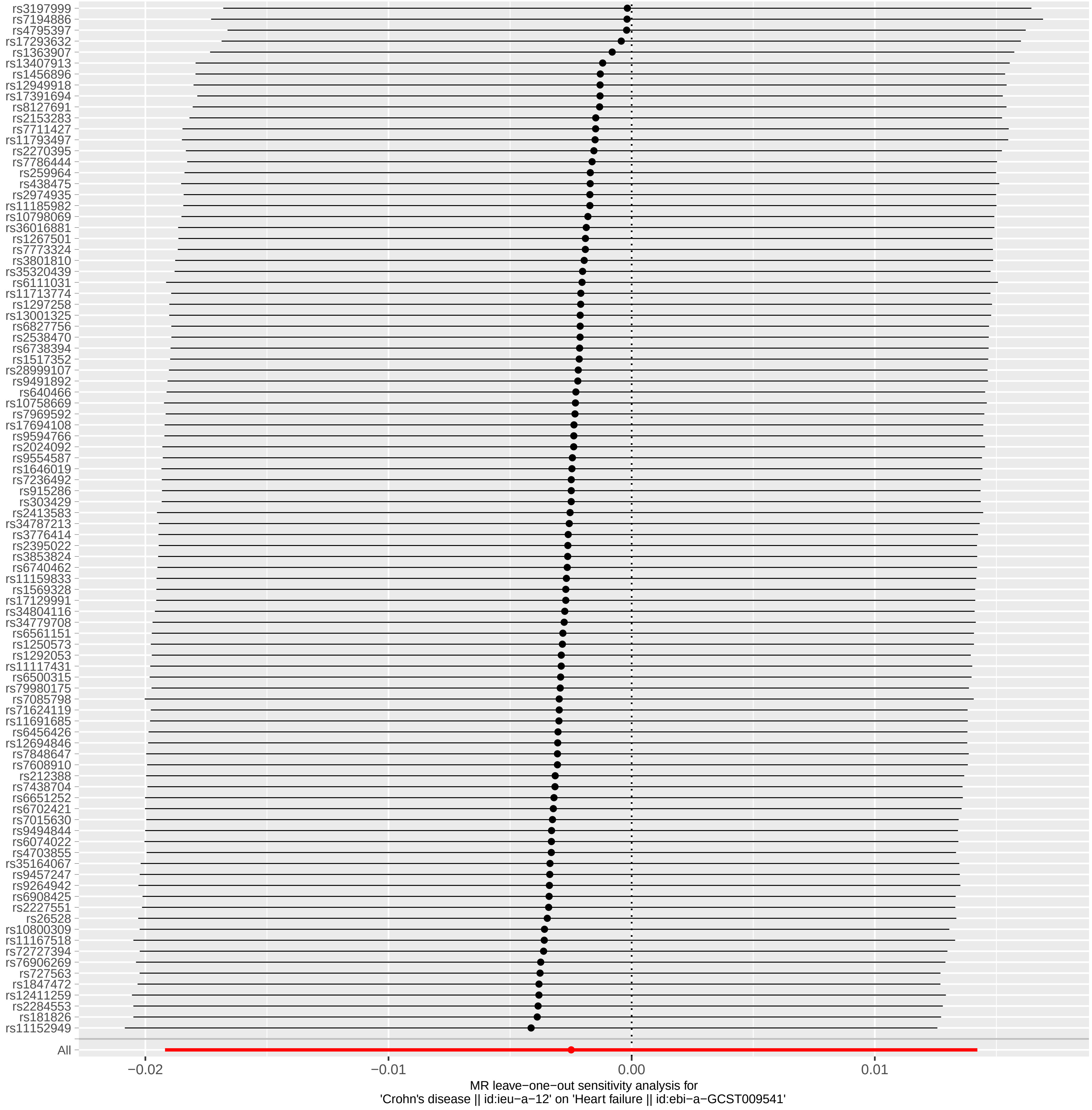

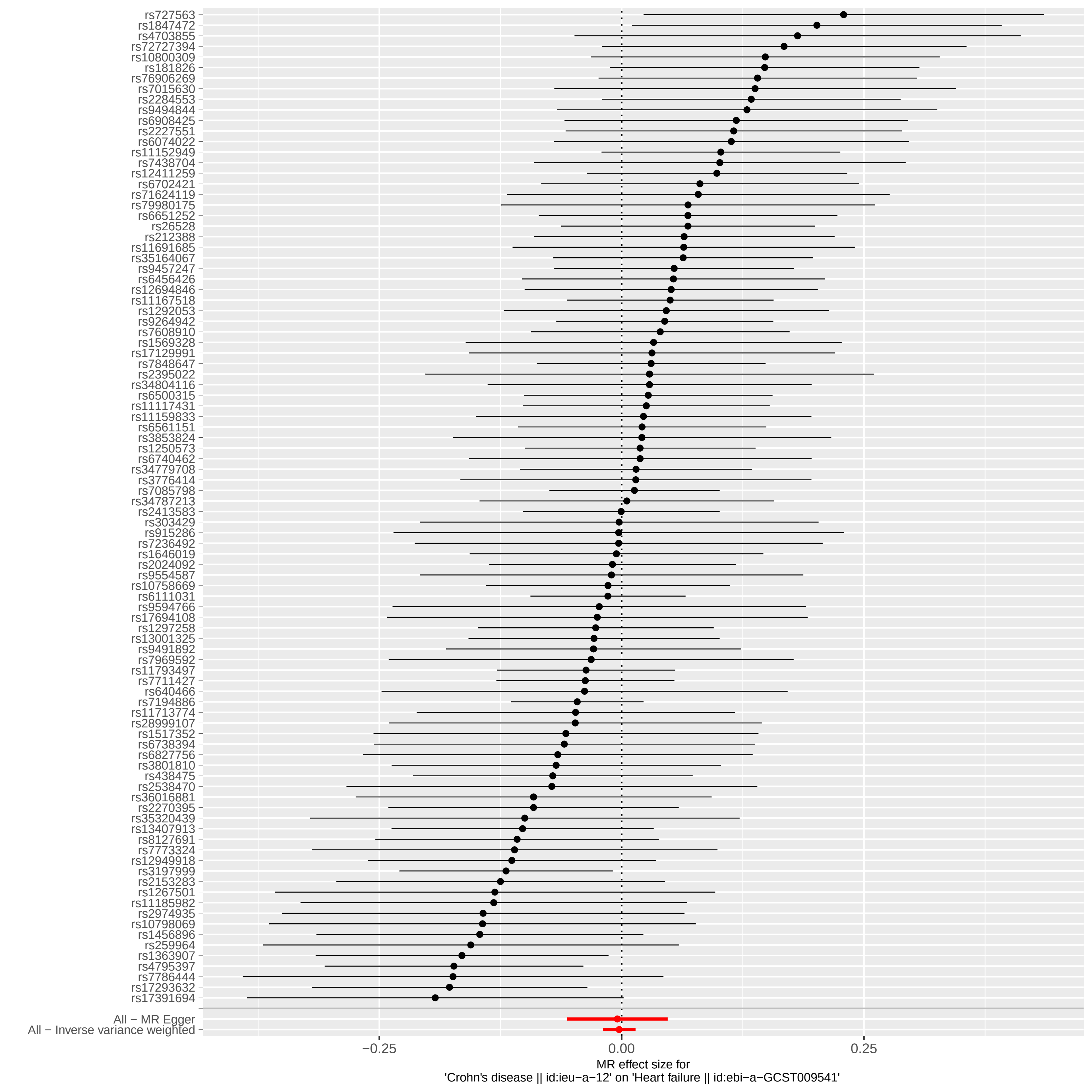
rs727563
rs1847472
rs4703855
rs72727394
rs10800309
rs181826
rs76906269
rs7015630
rs2284553
rs9494844
rs6908425
rs2227551
rs6074022
rs11152949
rs7438704
rs12411259
rs6702421
rs71624119
rs79980175
rs6651252
rs26528
rs212388
rs11691685
rs35164067
rs9457247
rs6456426
rs12694846
rs11167518
rs1292053
rs9264942
rs7608910
rs1569328
rs17129991
rs7848647
rs2395022
rs34804116
rs6500315
rs11117431
rs11159833
rs6561151
rs3853824
rs1250573
rs6740462
rs34779708
rs3776414
rs7085798
rs34787213
rs2413583
rs303429
rs915286
rs7236492
rs1646019
rs2024092
rs9554587
rs10758669
rs6111031
rs9594766
rs17694108
rs1297258
rs13001325
rs9491892
rs7969592
rs11793497
rs7711427
rs640466
rs7194886
rs11713774
rs28999107
rs1517352
rs6738394
rs6827756
rs3801810
rs438475
rs2538470
rs36016881
rs2270395
rs35320439
rs13407913
rs8127691
rs7773324
rs12949918
rs3197999
rs2153283
rs1267501
rs11185982
rs2974935
rs10798069
rs1456896
rs259964
rs1363907
rs4795397
rs7786444
rs17293632
rs17391694
All − MR Egger
All − Inverse variance weighted
−0.25
0.00
0.25
MR effect size for
'Crohn's disease || id:ieu−a−12' on 'Heart failure || id:ebi−a−GCST009541'

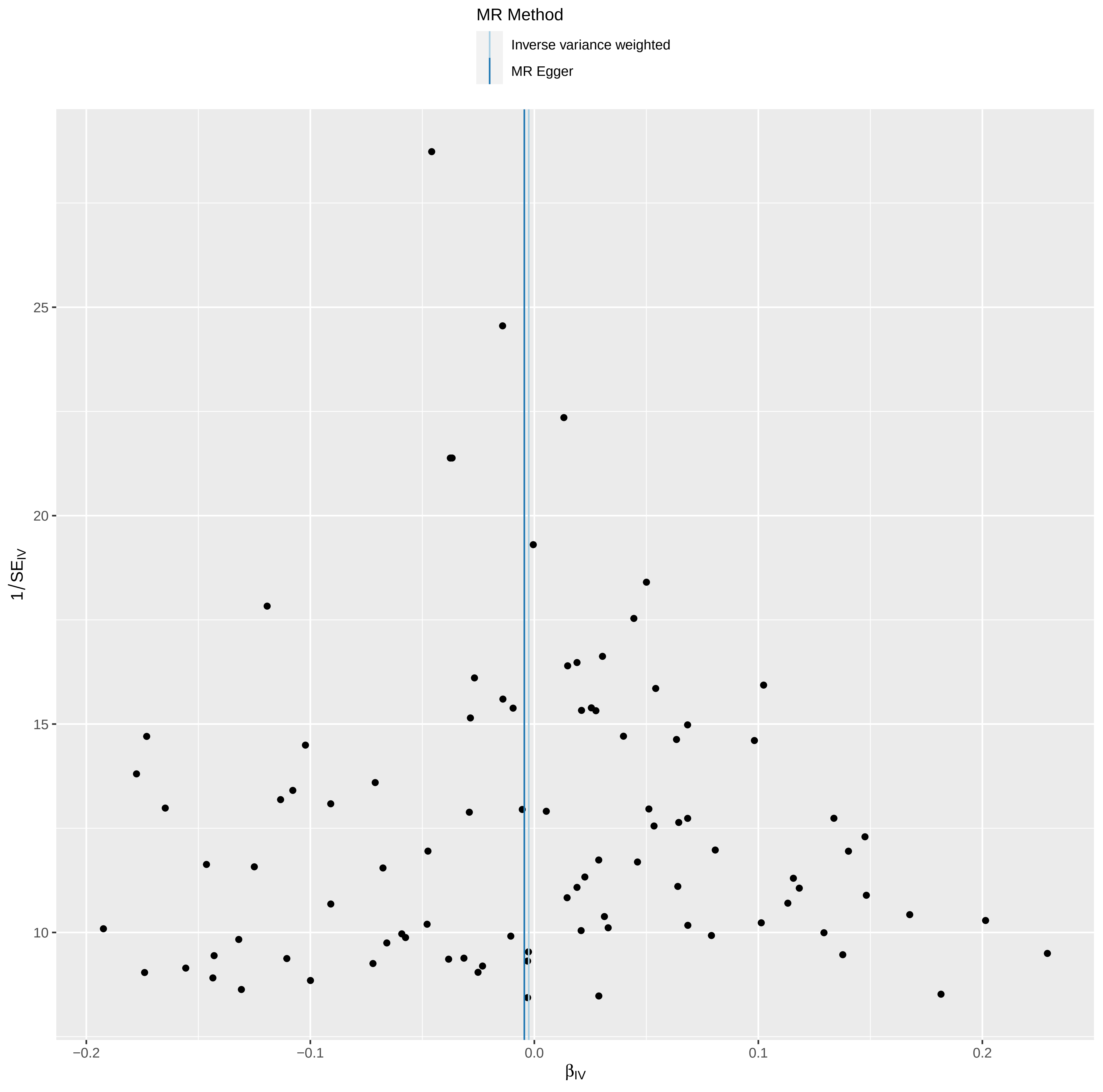
MR Method
Inverse variance weighted
MR Egger
1/SE$_{IV}$
25
20
15
10
−0.2
−0.1
0.0
0.1
0.2
β_{IV}

Figure 4 Leave-one-out analysis, MR effect size and funnel plot for Crohn's disease on cardiomyopathy.

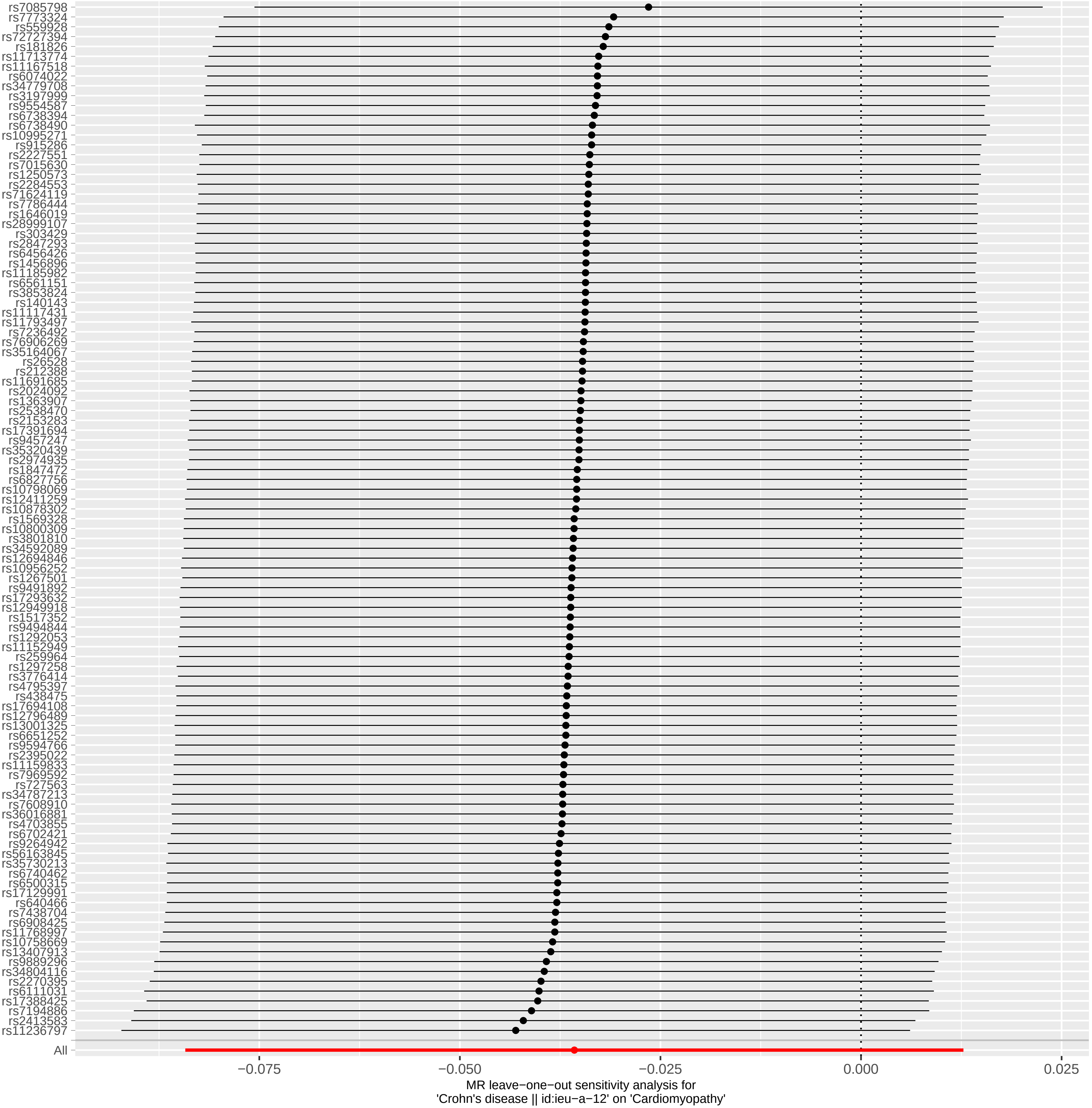

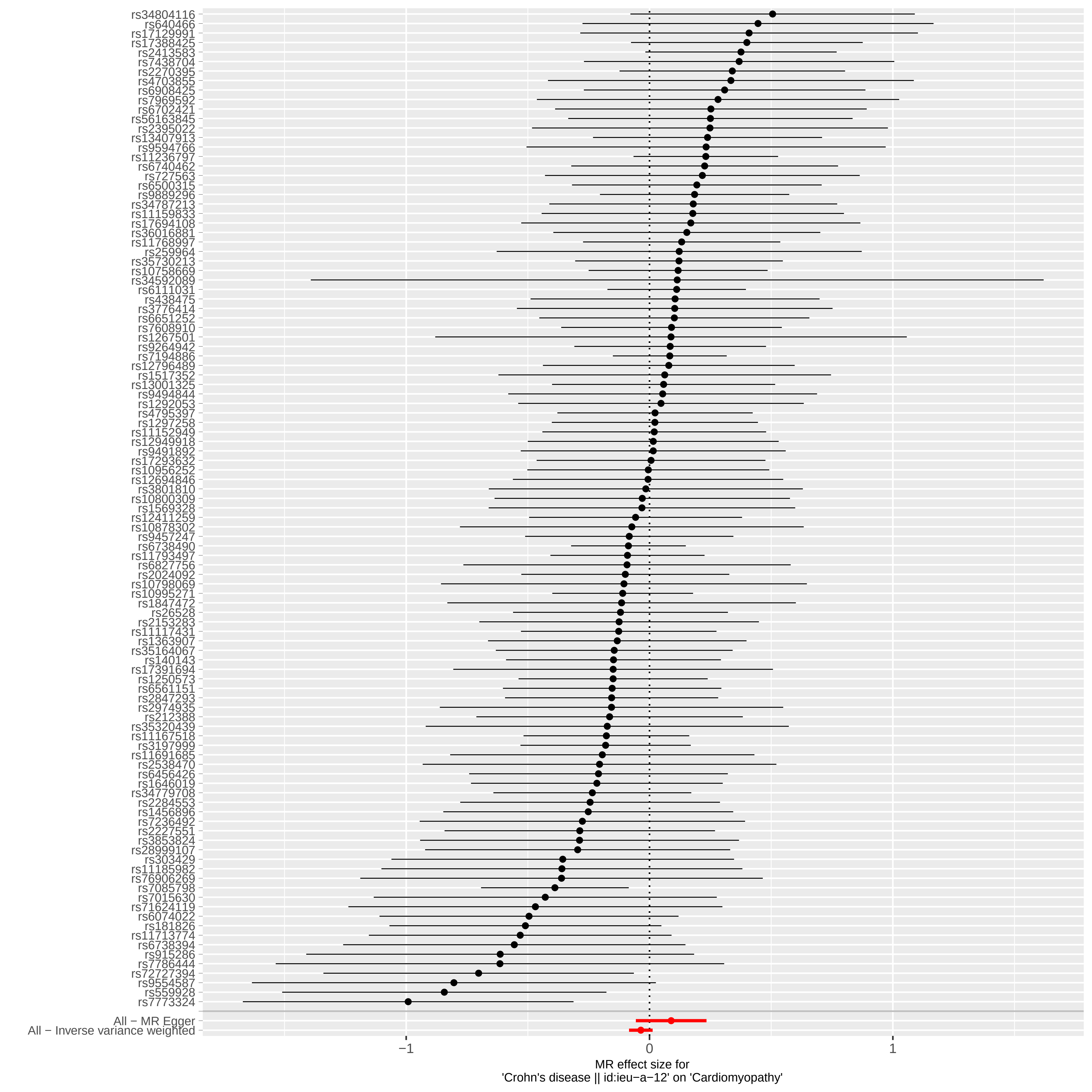
rs34804116
rs640466
rs17129991
rs17388425
rs2413583
rs7438704
rs2270395
rs4703855
rs6908425
rs7969592
rs6702421
rs56163845
rs2395022
rs13407913
rs9594766
rs11236797
rs6740462
rs727563
rs6500315
rs9889296
rs34787213
rs11159833
rs17694108
rs36016881
rs11768997
rs259964
rs35730213
rs10758669
rs34592089
rs6111031
rs438475
rs3776414
rs6651252
rs7608910
rs1267501
rs9264942
rs7194886
rs12796489
rs1517352
rs13001325
rs9494844
rs1292053
rs4795397
rs1297258
rs11152949
rs12949918
rs9491892
rs17293632
rs10956252
rs12694846
rs3801810
rs10800309
rs1569328
rs12411259
rs10878302
rs9457247
rs6738490
rs11793497
rs6827756
rs2024092
rs10798069
rs10995271
rs1847472
rs26528
rs2153283
rs11117431
rs1363907
rs35164067
rs140143
rs17391694
rs1250573
rs6561151
rs2847293
rs2974935
rs212388
rs35320439
rs11167518
rs3197999
rs11691685
rs2538470
rs6456426
rs1646019
rs34779708
rs2284553
rs1456896
rs7236492
rs2227551
rs3853824
rs28999107
rs303429
rs11185982
rs76906269
rs7085798
rs7015630
rs71624119
rs6074022
rs181826
rs11713774
rs6738394
rs915286
rs7786444
rs72727394
rs9554587
rs559928
rs7773324
All − MR Egger
All − Inverse variance weighted
−1
0
1
MR effect size for
'Crohn's disease || id:ieu−a−12' on 'Cardiomyopathy'

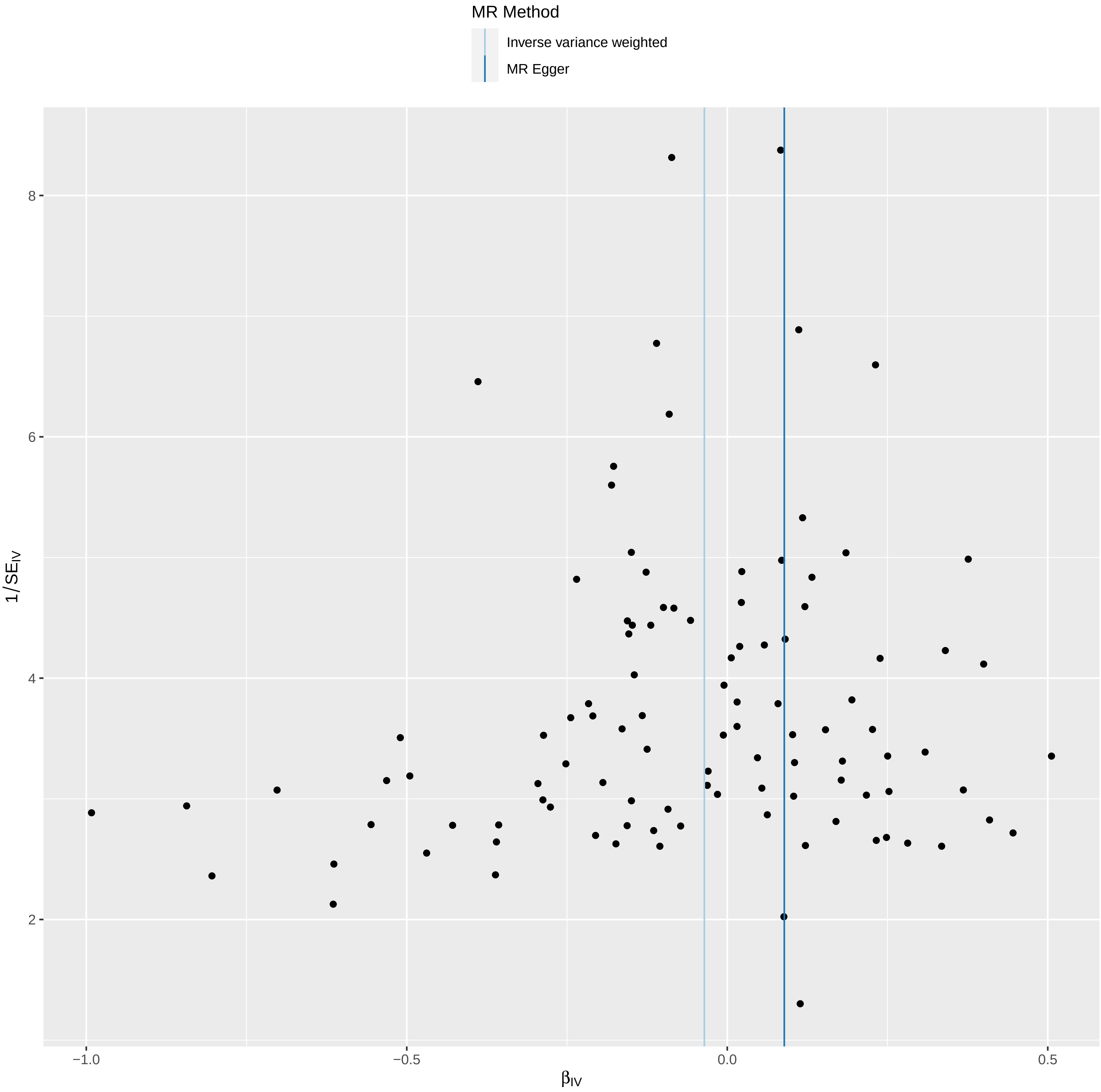

MR Method
Inverse variance weighted
MR Egger
1/SE$_{IV}$
8
6
4
2
−1.0
−0.5
0.0
0.5
β$_{IV}$

Figure 5 Leave-one-out analysis, MR effect size and funnel plot for Crohn's disease on venous thromboembolism.

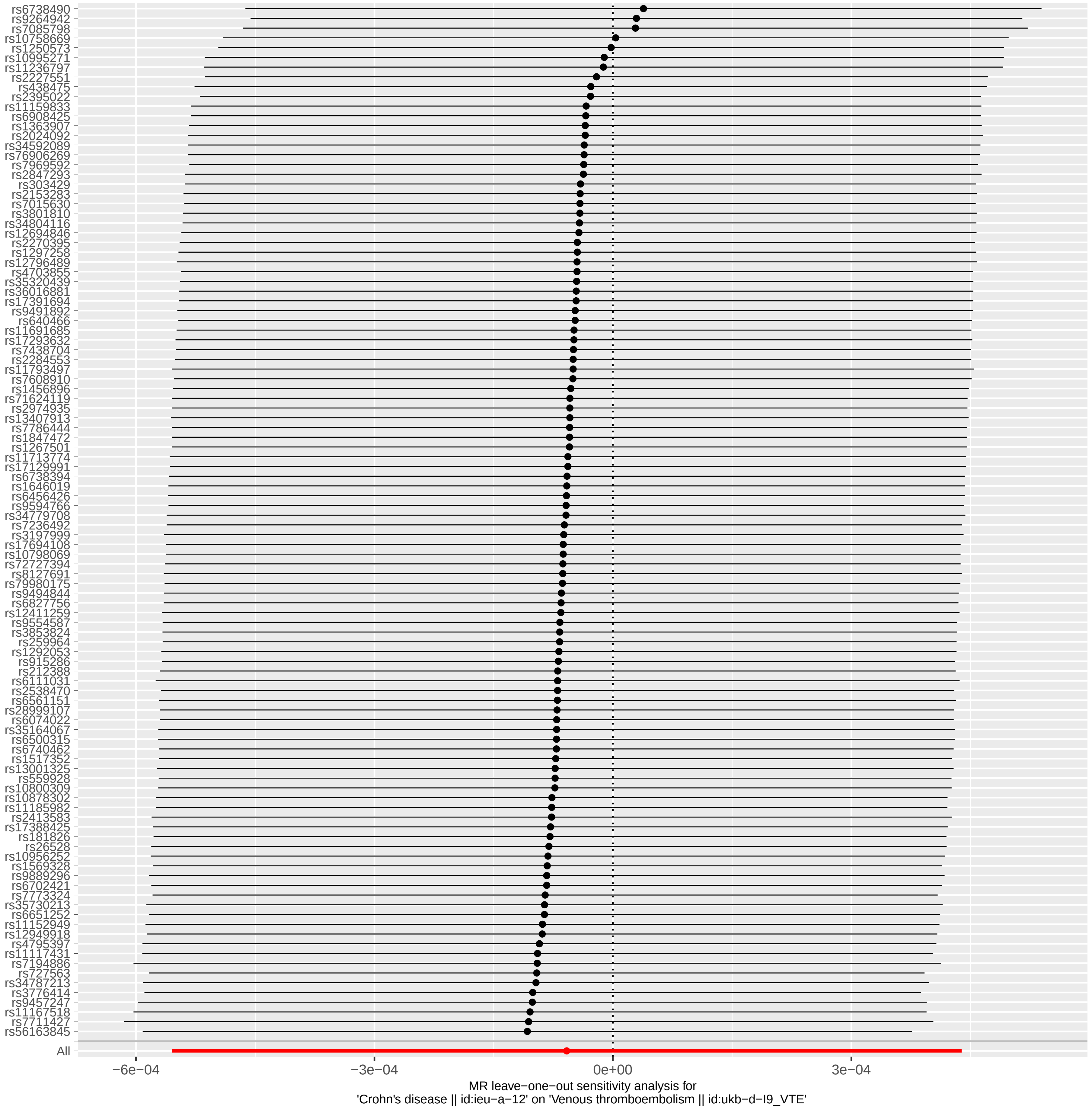

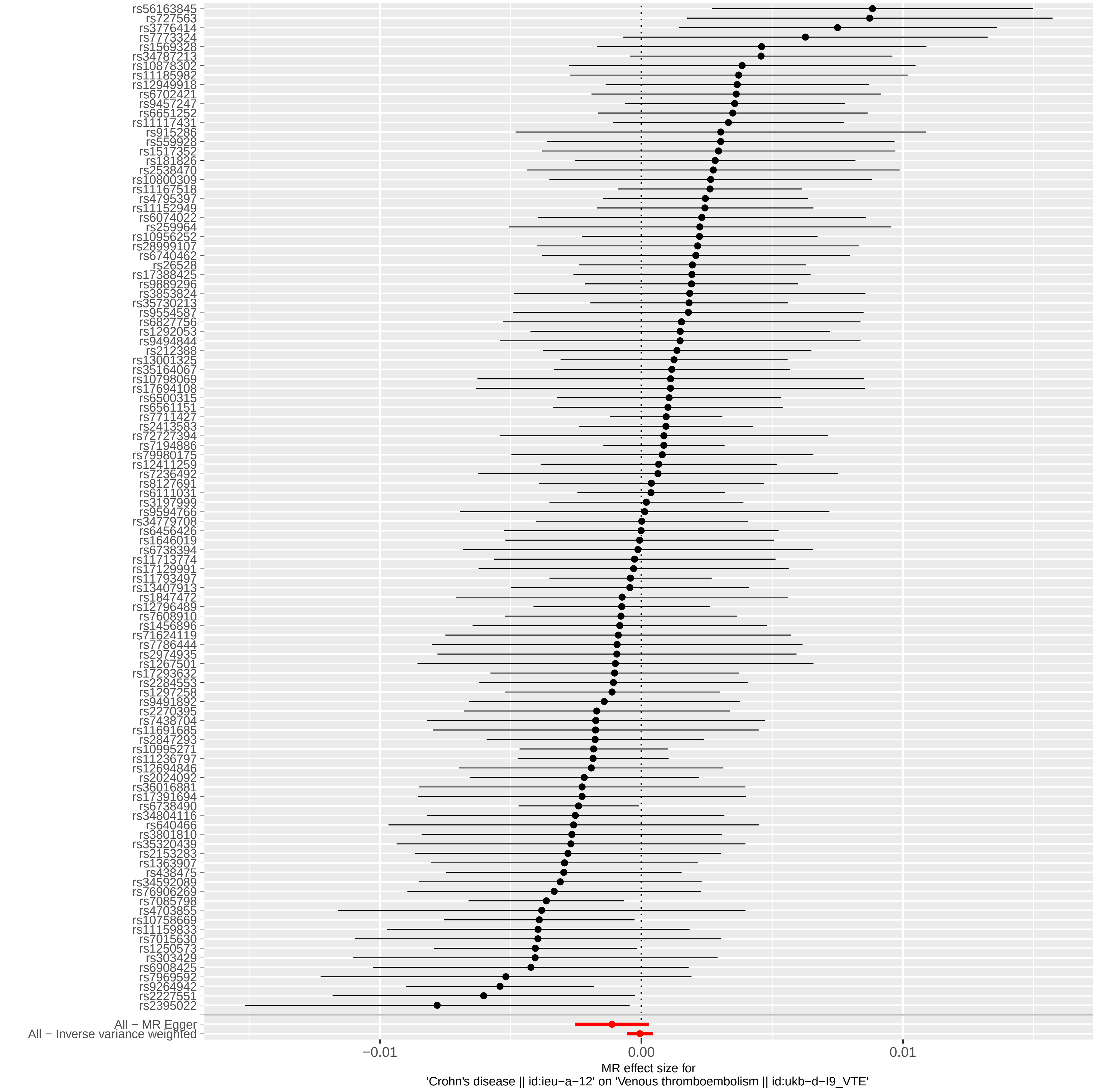

rs56163845
rs727563
rs3776414
rs7773324
rs1569328
rs34787213
rs10878302
rs11185982
rs12949918
rs6702421
rs9457247
rs6651252
rs11117431
rs915286
rs559928
rs1517352
rs181826
rs2538470
rs10800309
rs11167518
rs4795397
rs11152949
rs6074022
rs259964
rs10956252
rs28999107
rs6740462
rs26528
rs17388425
rs9889296
rs3853824
rs35730213
rs9554587
rs6827756
rs1292053
rs9494844
rs212388
rs13001325
rs35164067
rs10798069
rs17694108
rs6500315
rs6561151
rs7711427
rs2413583
rs72727394
rs7194886
rs79980175
rs12411259
rs7236492
rs8127691
rs6111031
rs3197999
rs9594766
rs34779708
rs6456426
rs1646019
rs6738394
rs11713774
rs17129991
rs11793497
rs13407913
rs1847472
rs12796489
rs7608910
rs1456896
rs71624119
rs7786444
rs2974935
rs1267501
rs17293632
rs2284553
rs1297258
rs9491892
rs2270395
rs7438704
rs11691685
rs2847293
rs10995271
rs11236797
rs12694846
rs2024092
rs36016881
rs17391694
rs6738490
rs34804116
rs640466
rs3801810
rs35320439
rs2153283
rs1363907
rs438475
rs34592089
rs76906269
rs7085798
rs4703855
rs10758669
rs11159833
rs7015630
rs1250573
rs303429
rs6908425
rs7969592
rs9264942
rs2227551
rs2395022
All − MR Egger
All − Inverse variance weighted
−0.01
0.00
0.01
MR effect size for
'Crohn's disease || id:ieu−a−12' on 'Venous thromboembolism || id:ukb−d−I9_VTE'

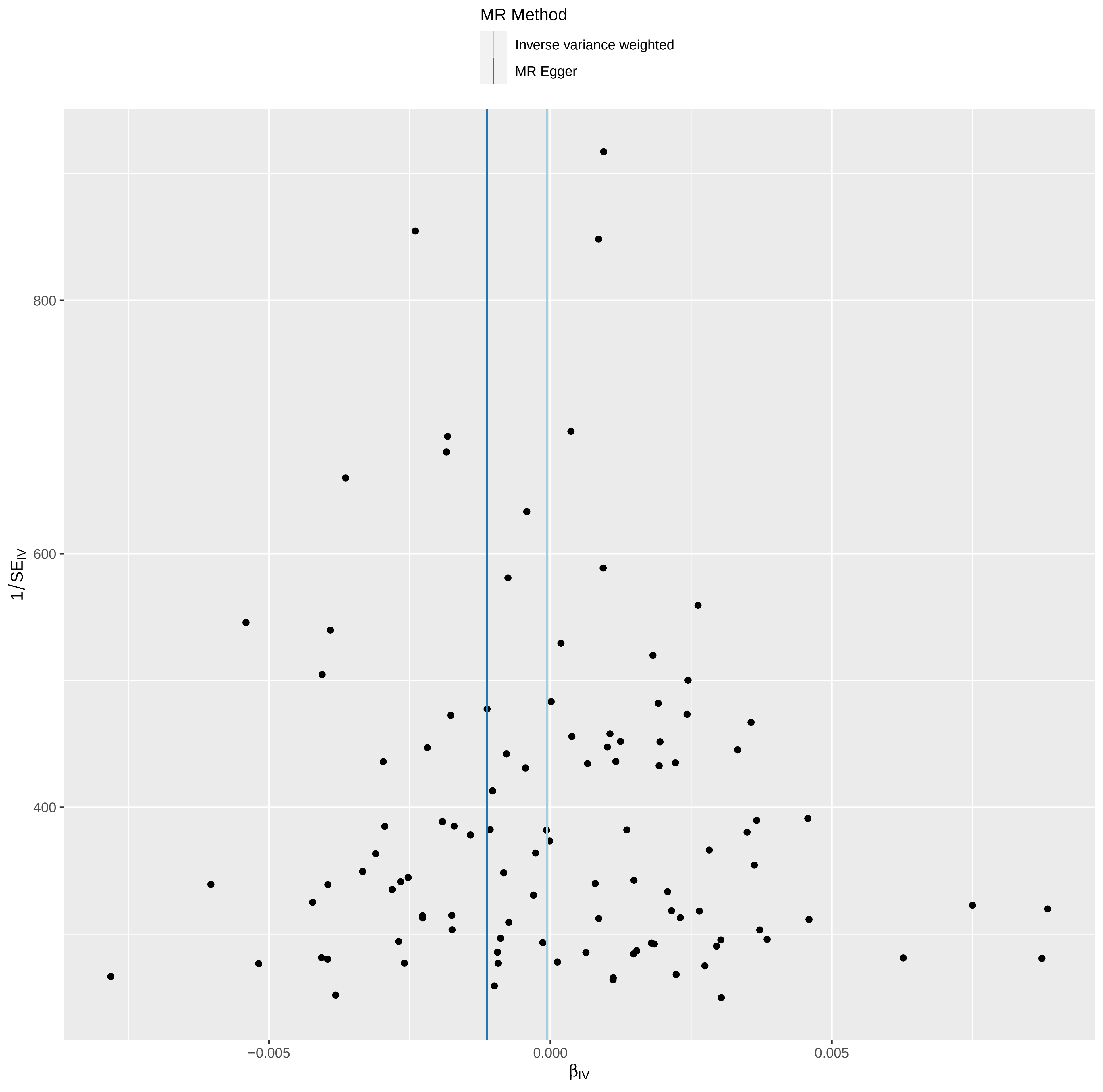

MR Method
Inverse variance weighted
MR Egger
1/SE_IV
800
600
400
−0.005
0.000
0.005
β_IV

Figure 6 Leave-one-out analysis, MR effect size and funnel plot for Crohn's disease on deep venous thrombosis.

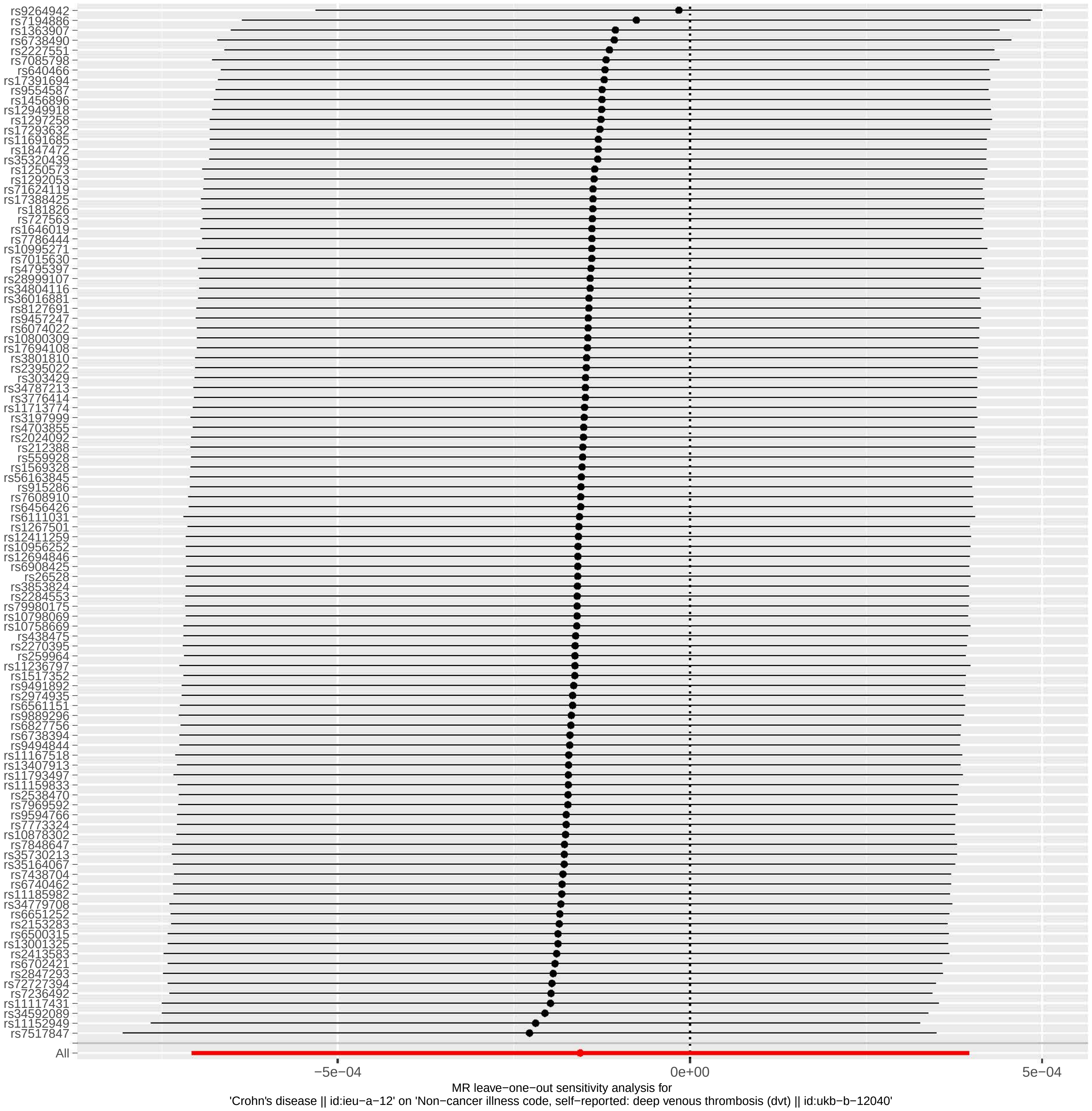

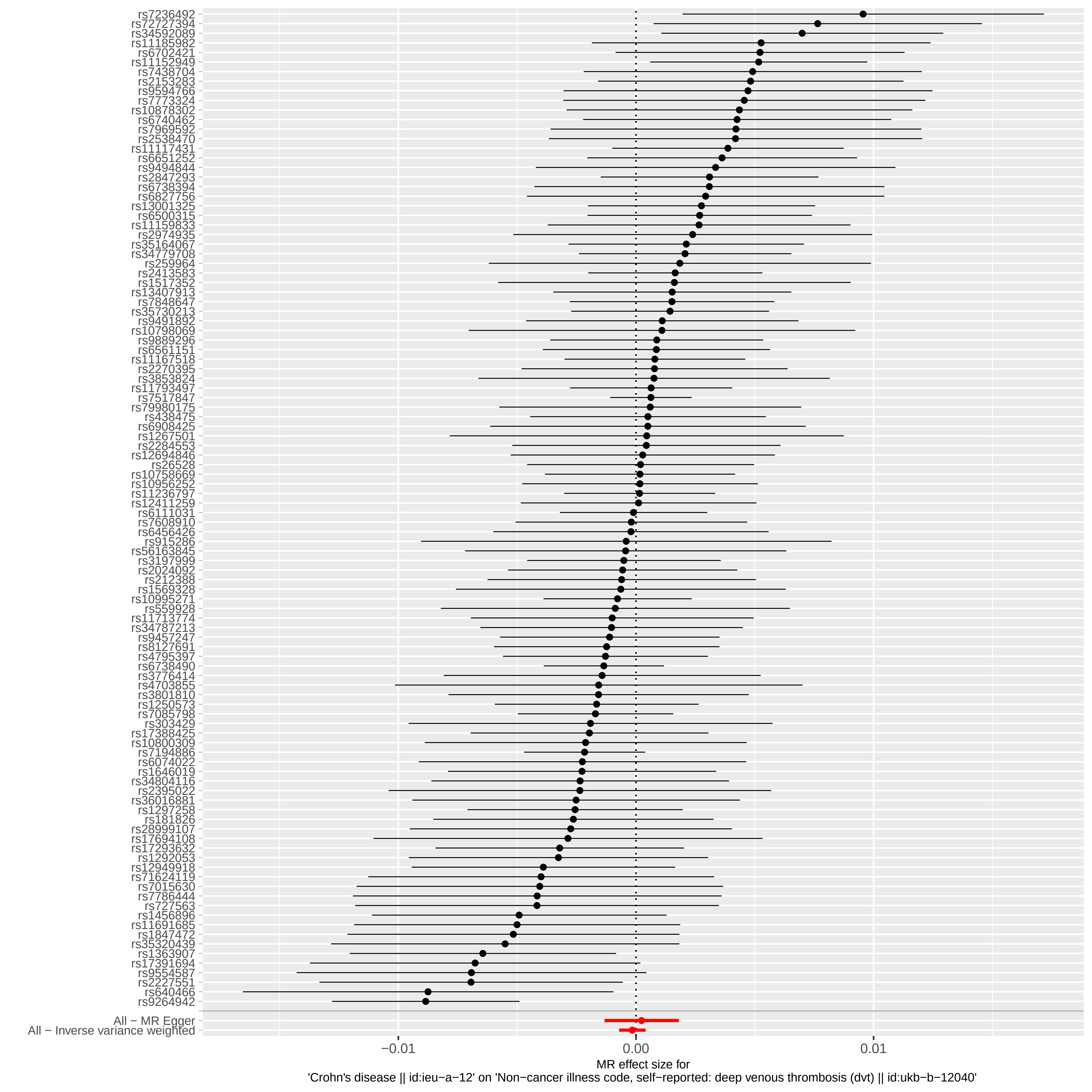

rs7236492
rs72727394
rs34592089
rs11185982
rs6702421
rs11152949
rs7438704
rs2153283
rs9594766
rs7773324
rs10878302
rs6740462
rs7969592
rs2538470
rs11117431
rs6651252
rs9494844
rs2847293
rs6738394
rs6827756
rs13001325
rs6500315
rs11159833
rs2974935
rs35164067
rs34779708
rs259964
rs2413583
rs1517352
rs13407913
rs7848647
rs35730213
rs9491892
rs10798069
rs9889296
rs6561151
rs11167518
rs2270395
rs3853824
rs11793497
rs7517847
rs79980175
rs438475
rs6908425
rs1267501
rs2284553
rs12694846
rs26528
rs10758669
rs10956252
rs11236797
rs12411259
rs6111031
rs7608910
rs6456426
rs915286
rs56163845
rs3197999
rs2024092
rs212388
rs1569328
rs10995271
rs559928
rs11713774
rs34787213
rs9457247
rs8127691
rs4795397
rs6738490
rs3776414
rs4703855
rs3801810
rs1250573
rs7085798
rs303429
rs17388425
rs10800309
rs7194886
rs6074022
rs1646019
rs34804116
rs2395022
rs36016881
rs1297258
rs181826
rs28999107
rs17694108
rs17293632
rs1292053
rs12949918
rs71624119
rs7015630
rs7786444
rs727563
rs1456896
rs11691685
rs1847472
rs35320439
rs1363907
rs17391694
rs9554587
rs2227551
rs640466
rs9264942
All − MR Egger
All − Inverse variance weighted
−0.01
0.00
0.01
MR effect size for
'Crohn's disease || id:ieu−a−12' on 'Non−cancer illness code, self−reported: deep venous thrombosis (dvt) || id:ukb−b−12040'

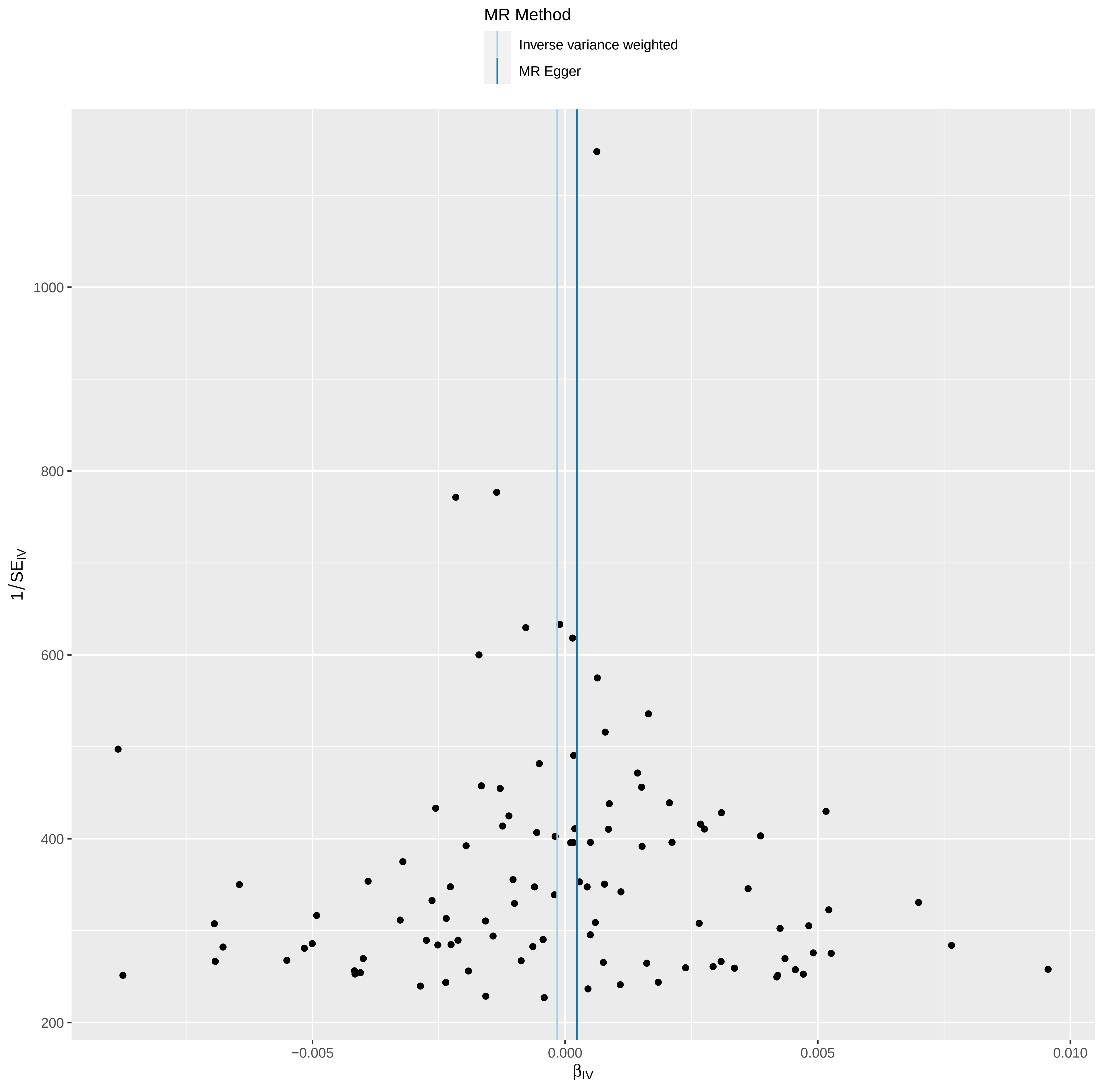

MR Method
Inverse variance weighted
MR Egger
1/SE$_{IV}$
β_{IV}
200
400
600
800
1000
−0.005
0.000
0.005
0.010

Figure 7 Leave-one-out analysis, MR effect size and funnel plot for Crohn's disease on atrial fibrillation.

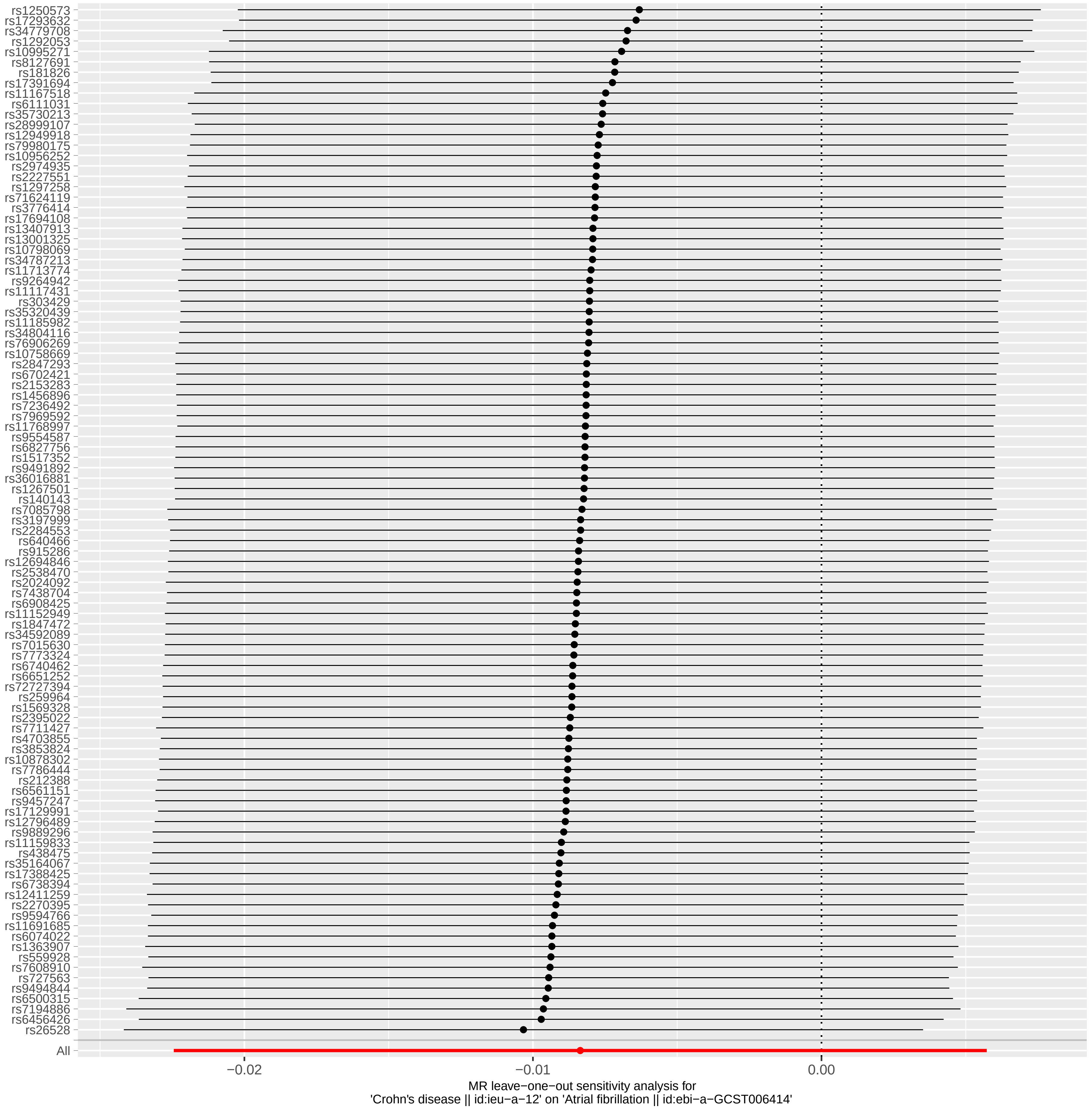

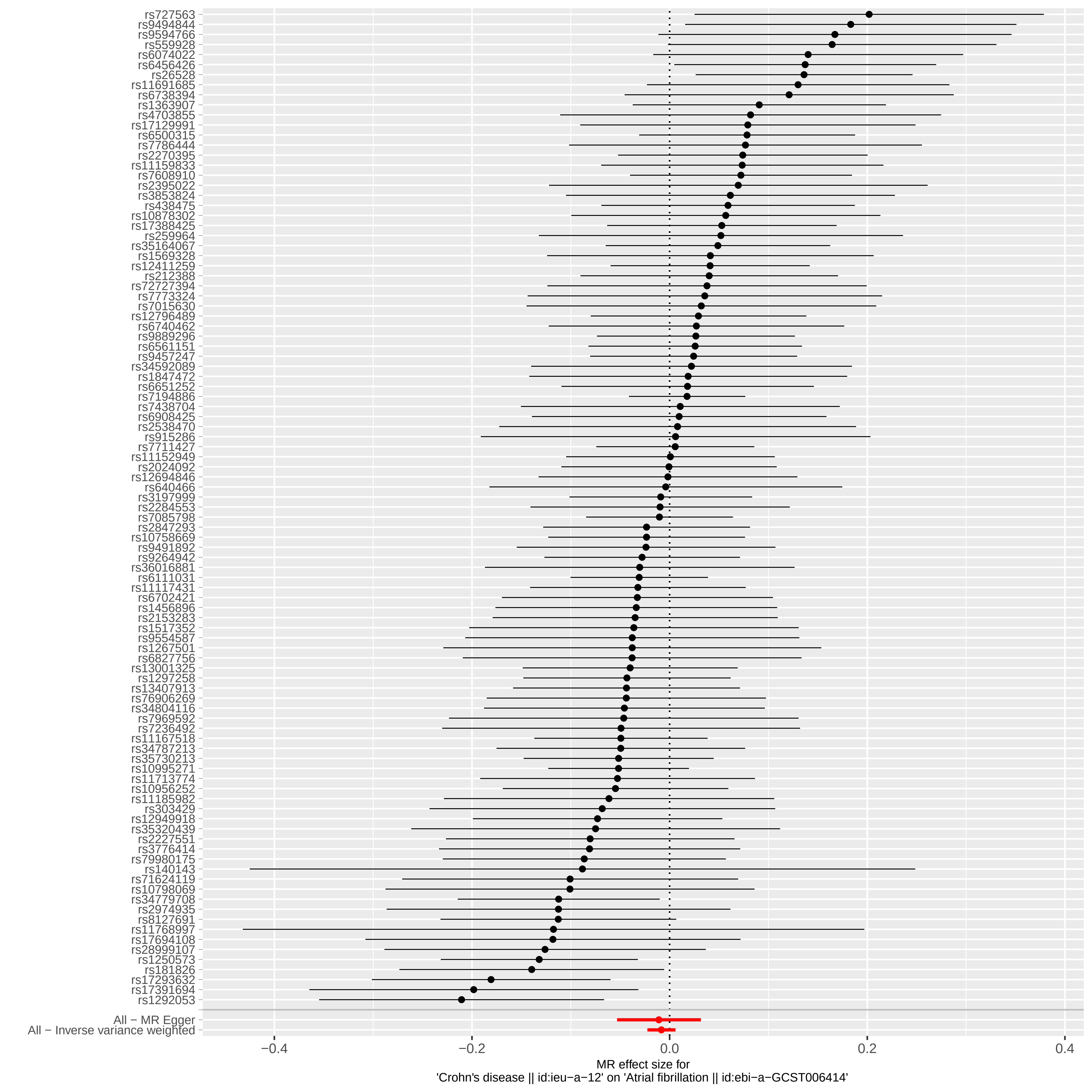

rs727563
rs9494844
rs9594766
rs559928
rs6074022
rs6456426
rs26528
rs11691685
rs6738394
rs1363907
rs4703855
rs17129991
rs6500315
rs7786444
rs2270395
rs11159833
rs7608910
rs2395022
rs3853824
rs438475
rs10878302
rs17388425
rs259964
rs35164067
rs1569328
rs12411259
rs212388
rs72727394
rs7773324
rs7015630
rs12796489
rs6740462
rs9889296
rs6561151
rs9457247
rs34592089
rs1847472
rs6651252
rs7194886
rs7438704
rs6908425
rs2538470
rs915286
rs7711427
rs11152949
rs2024092
rs12694846
rs640466
rs3197999
rs2284553
rs7085798
rs2847293
rs10758669
rs9491892
rs9264942
rs36016881
rs6111031
rs11117431
rs6702421
rs1456896
rs2153283
rs1517352
rs9554587
rs1267501
rs6827756
rs13001325
rs1297258
rs13407913
rs76906269
rs34804116
rs7969592
rs7236492
rs11167518
rs34787213
rs35730213
rs10995271
rs11713774
rs10956252
rs11185982
rs303429
rs12949918
rs35320439
rs2227551
rs3776414
rs79980175
rs140143
rs71624119
rs10798069
rs34779708
rs2974935
rs8127691
rs11768997
rs17694108
rs28999107
rs1250573
rs181826
rs17293632
rs17391694
rs1292053
All − MR Egger
All − Inverse variance weighted
−0.4
−0.2
0.0
0.2
0.4
MR effect size for
'Crohn's disease || id:ieu−a−12' on 'Atrial fibrillation || id:ebi−a−GCST006414'

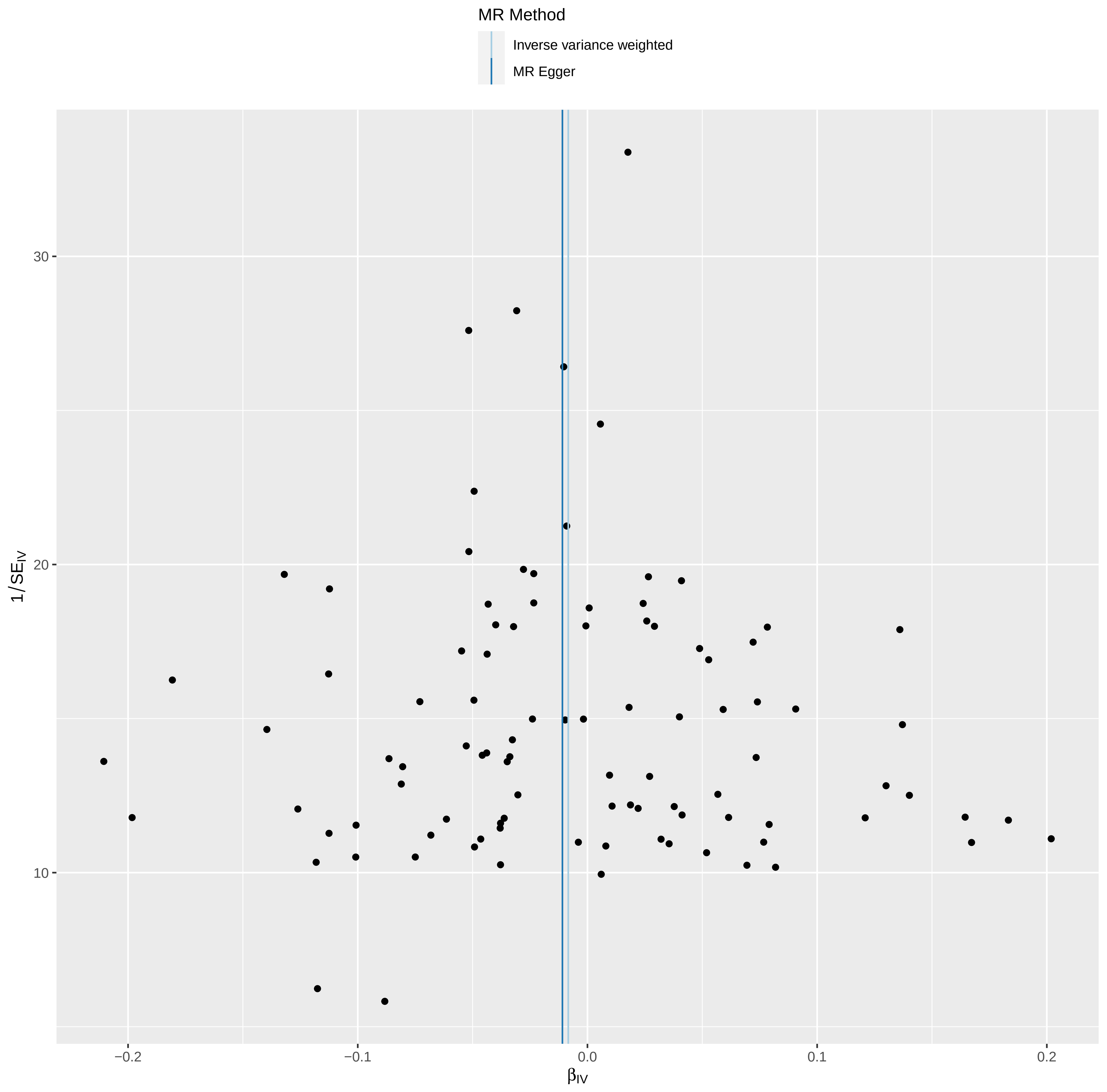

MR Method
Inverse variance weighted
MR Egger
30
20
10
1/SE_IV
−0.2
−0.1
0.0
0.1
0.2
β_IV

Figure 8 Leave-one-out analysis, MR effect size and funnel plot for Crohn's disease on pulmonary embolism.

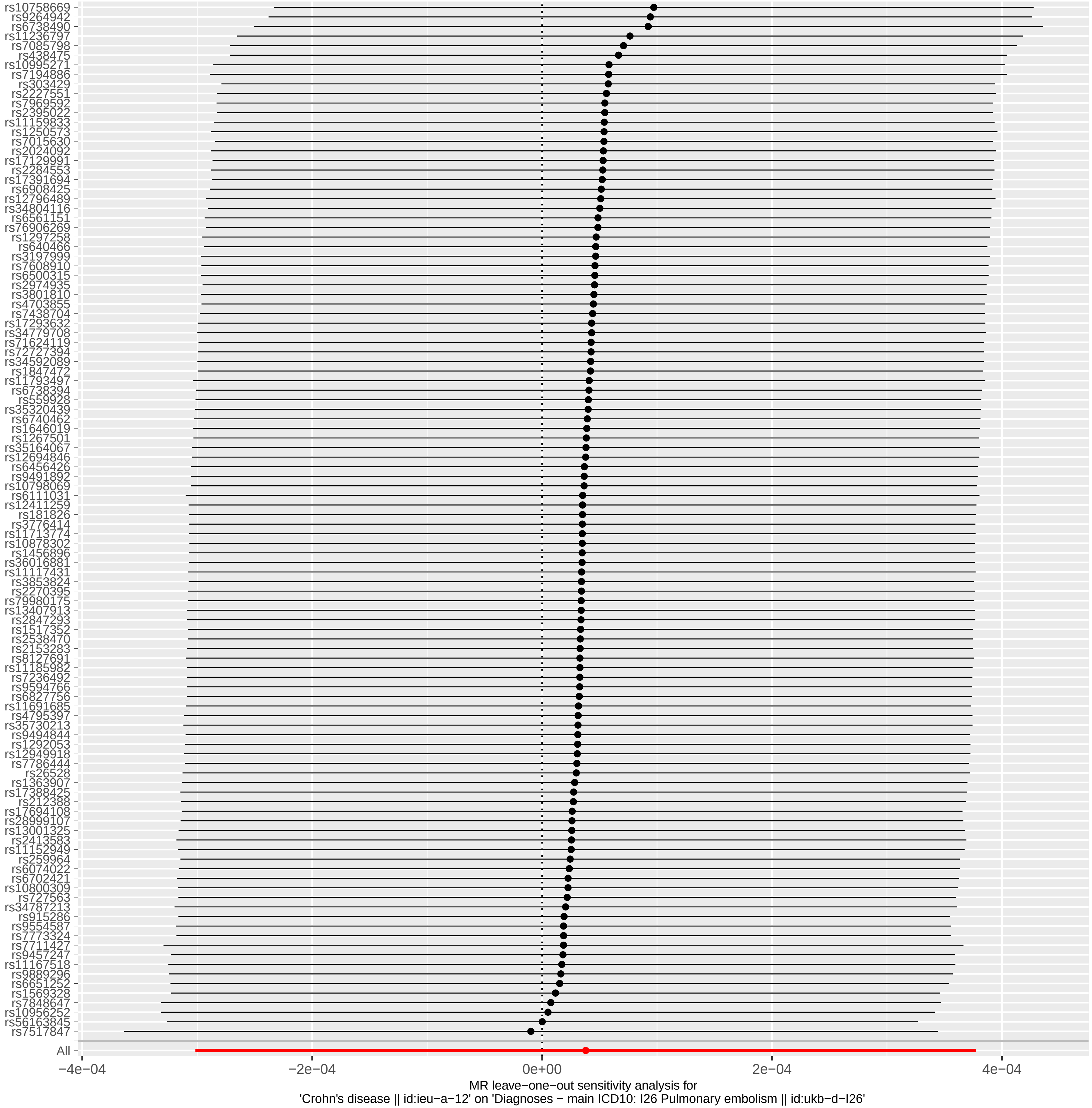

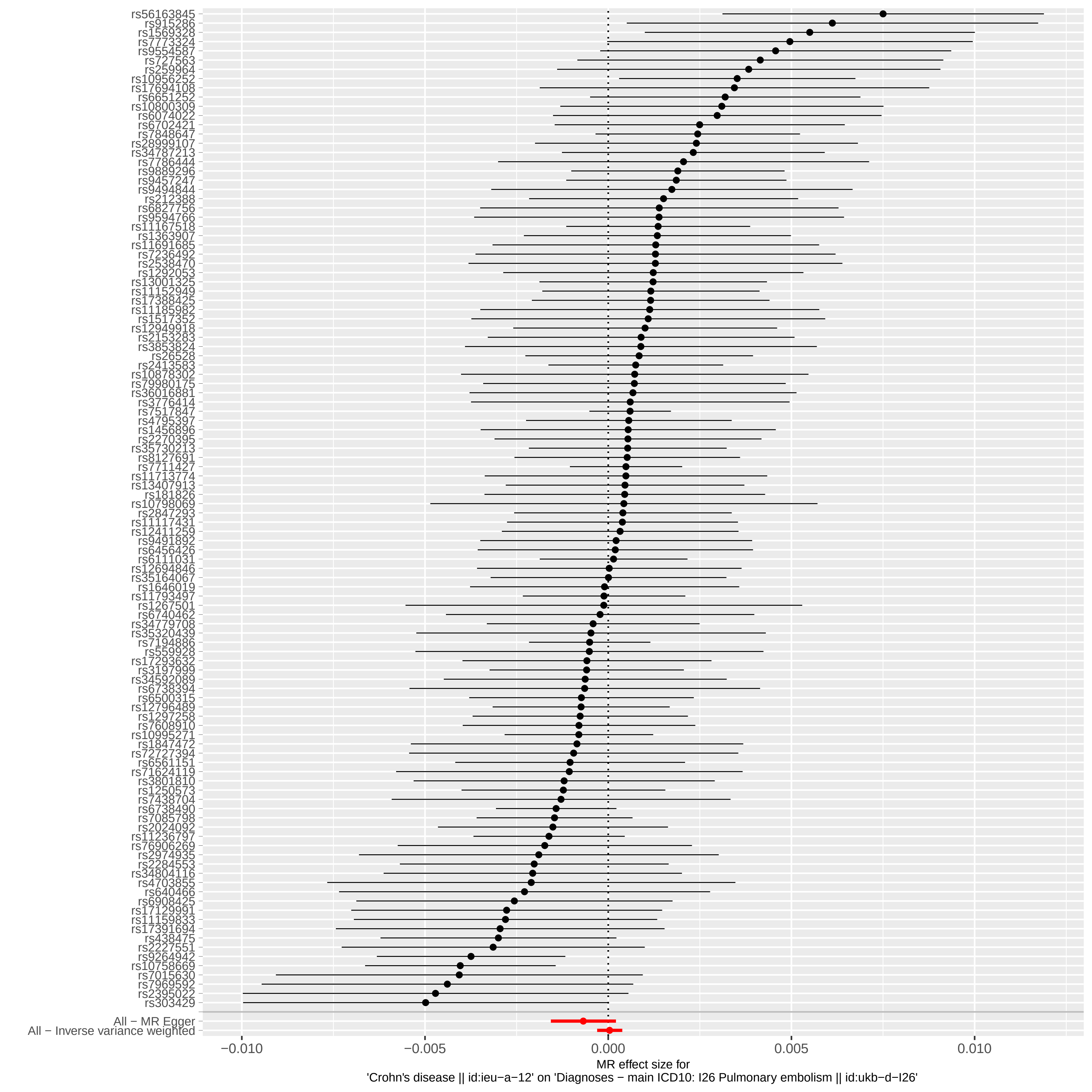
rs56163845
rs915286
rs1569328
rs7773324
rs9554587
rs727563
rs259964
rs10956252
rs17694108
rs6651252
rs10800309
rs6074022
rs6702421
rs7848647
rs28999107
rs34787213
rs7786444
rs9889296
rs9457247
rs9494844
rs212388
rs6827756
rs9594766
rs11167518
rs1363907
rs11691685
rs7236492
rs2538470
rs1292053
rs13001325
rs11152949
rs17388425
rs11185982
rs1517352
rs12949918
rs2153283
rs3853824
rs26528
rs2413583
rs10878302
rs79980175
rs36016881
rs3776414
rs7517847
rs4795397
rs1456896
rs2270395
rs35730213
rs8127691
rs7711427
rs11713774
rs13407913
rs181826
rs10798069
rs2847293
rs11117431
rs12411259
rs9491892
rs6456426
rs6111031
rs12694846
rs35164067
rs1646019
rs11793497
rs1267501
rs6740462
rs34779708
rs35320439
rs7194886
rs559928
rs17293632
rs3197999
rs34592089
rs6738394
rs6500315
rs12796489
rs1297258
rs7608910
rs10995271
rs1847472
rs72727394
rs6561151
rs71624119
rs3801810
rs1250573
rs7438704
rs6738490
rs7085798
rs2024092
rs11236797
rs76906269
rs2974935
rs2284553
rs34804116
rs4703855
rs640466
rs6908425
rs17129991
rs11159833
rs17391694
rs438475
rs2227551
rs9264942
rs10758669
rs7015630
rs7969592
rs2395022
rs303429
All − MR Egger
All − Inverse variance weighted
−0.010
−0.005
0.000
0.005
0.010
MR effect size for
'Crohn's disease || id:ieu−a−12' on 'Diagnoses − main ICD10: I26 Pulmonary embolism || id:ukb−d−I26'

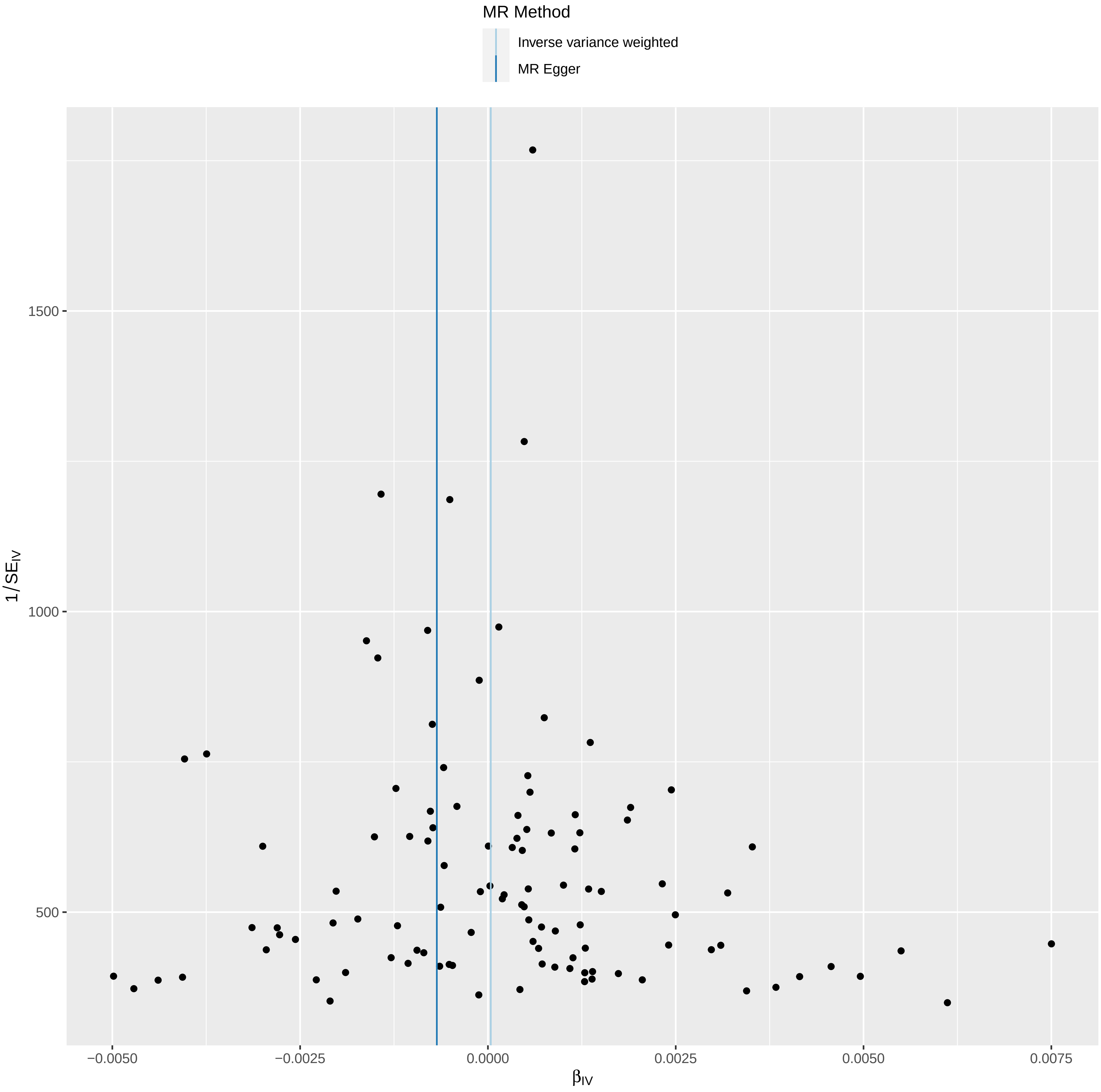
MR Method
Inverse variance weighted
MR Egger
1500
1000
500
1/SE_IV
−0.0050
−0.0025
0.0000
0.0025
0.0050
0.0075
β_IV

Figure 9 Leave-one-out analysis, MR effect size and funnel plot for Crohn's disease on peripheral arterial disease.

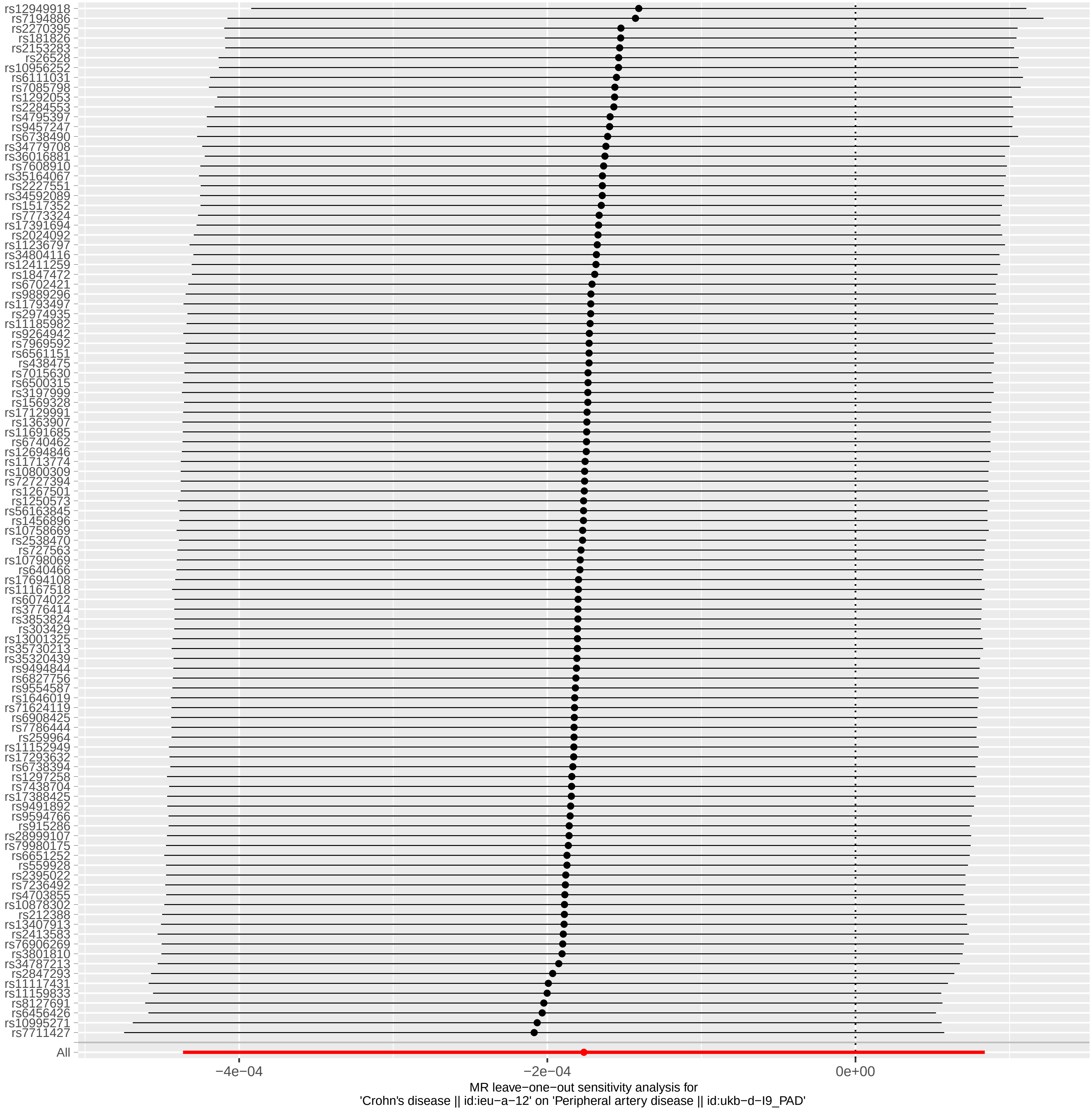

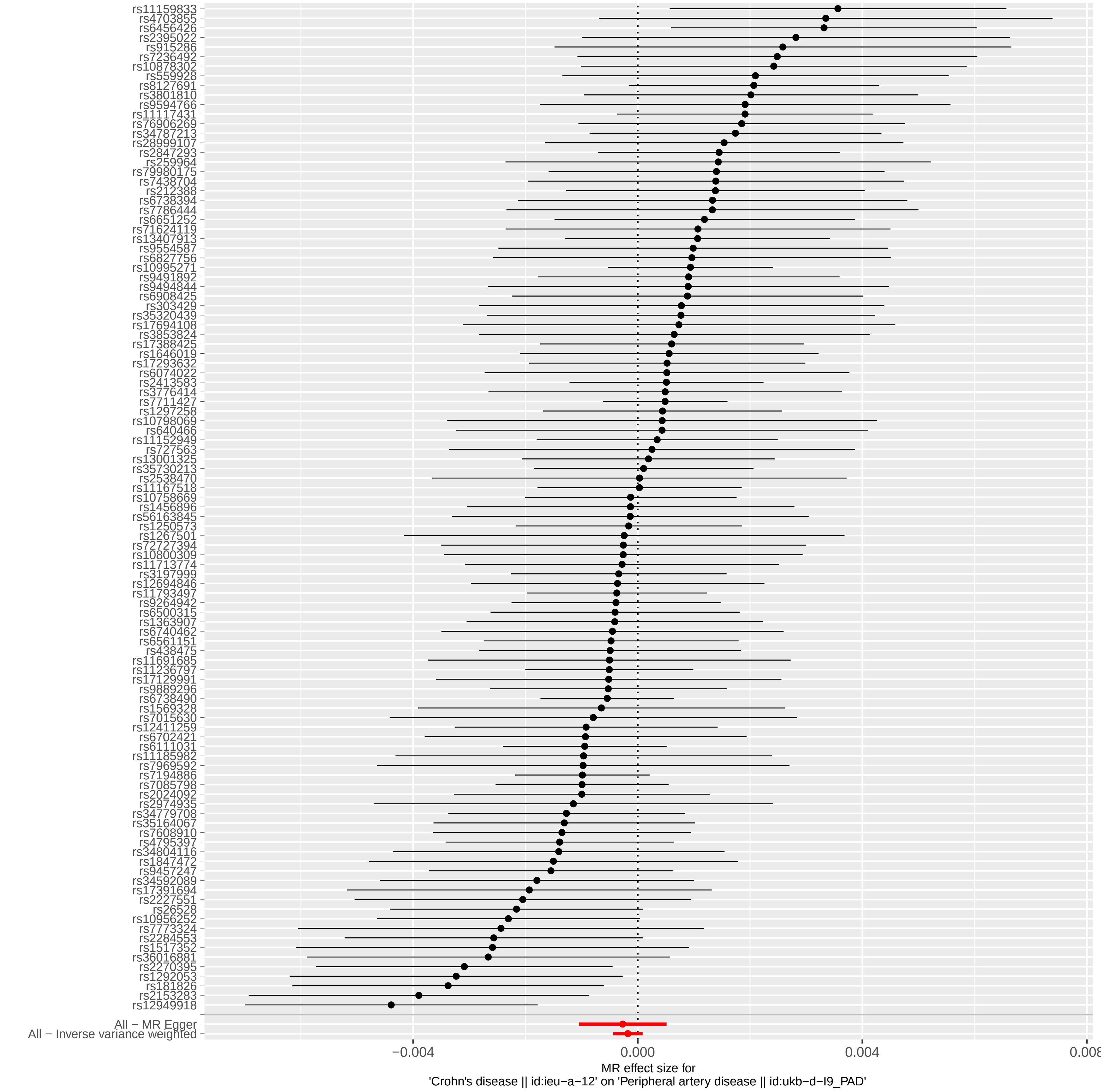
rs11159833
rs4703855
rs6456426
rs2395022
rs915286
rs7236492
rs10878302
rs559928
rs8127691
rs3801810
rs9594766
rs11117431
rs76906269
rs34787213
rs28999107
rs2847293
rs259964
rs79980175
rs7438704
rs212388
rs6738394
rs7786444
rs6651252
rs71624119
rs13407913
rs9554587
rs6827756
rs10995271
rs9491892
rs9494844
rs6908425
rs303429
rs35320439
rs17694108
rs3853824
rs17388425
rs1646019
rs17293632
rs6074022
rs2413583
rs3776414
rs7711427
rs1297258
rs10798069
rs640466
rs11152949
rs727563
rs13001325
rs35730213
rs2538470
rs11167518
rs10758669
rs1456896
rs56163845
rs1250573
rs1267501
rs72727394
rs10800309
rs11713774
rs3197999
rs12694846
rs11793497
rs9264942
rs6500315
rs1363907
rs6740462
rs6561151
rs438475
rs11691685
rs11236797
rs17129991
rs9889296
rs6738490
rs1569328
rs7015630
rs12411259
rs6702421
rs6111031
rs11185982
rs7969592
rs7194886
rs7085798
rs2024092
rs2974935
rs34779708
rs35164067
rs7608910
rs4795397
rs34804116
rs1847472
rs9457247
rs34592089
rs17391694
rs2227551
rs26528
rs10956252
rs7773324
rs2284553
rs1517352
rs36016881
rs2270395
rs1292053
rs181826
rs2153283
rs12949918
All − MR Egger
All − Inverse variance weighted
−0.004
0.000
0.004
0.008
MR effect size for
'Crohn's disease || id:ieu−a−12' on 'Peripheral artery disease || id:ukb−d−I9_PAD'

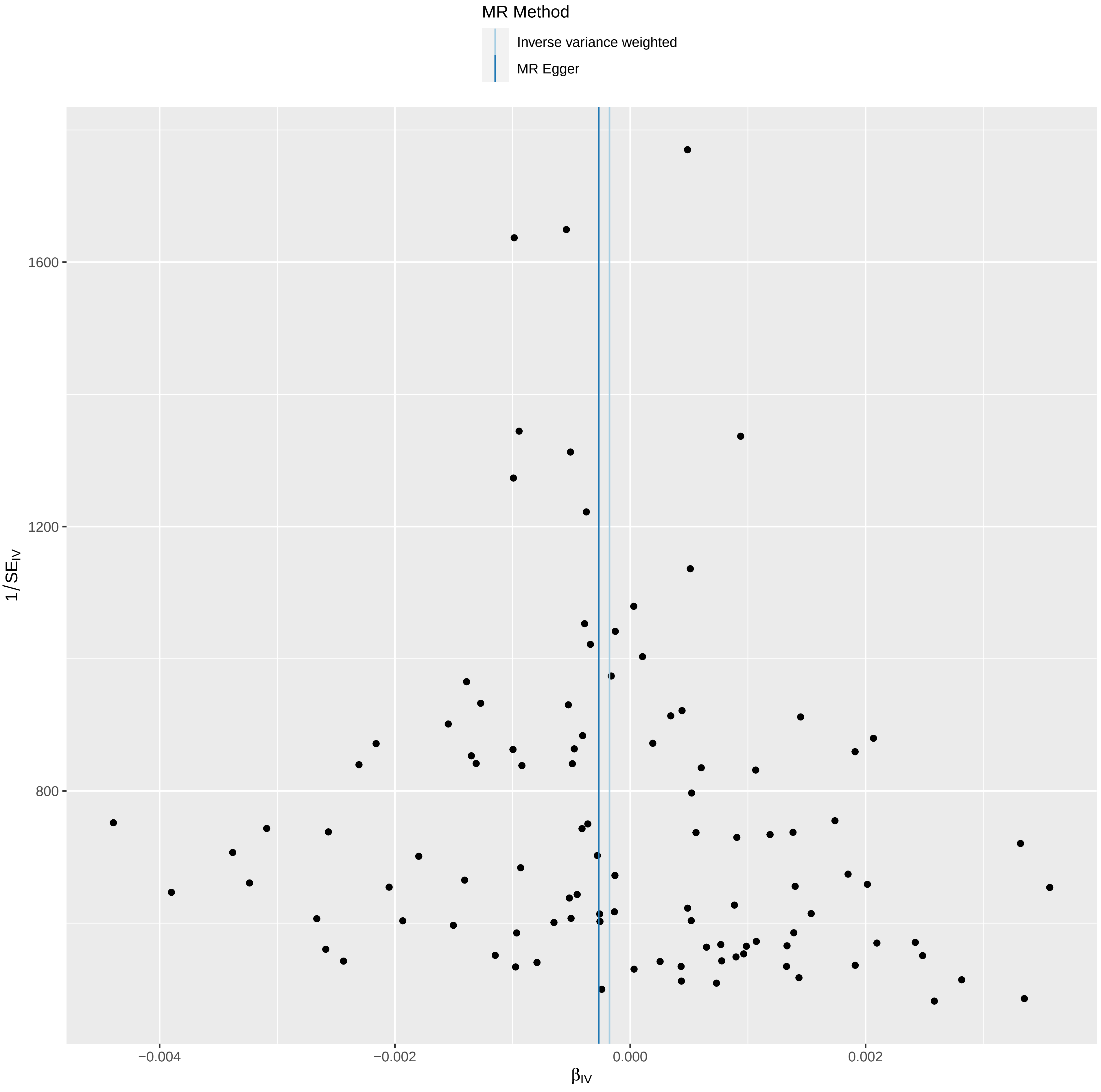

MR Method
Inverse variance weighted
MR Egger
1600
1200
800
1/SE$_{IV}$
−0.004
−0.002
0.000
0.002
β$_{IV}$

Figure 10 Leave-one-out analysis, MR effect size and funnel plot for Crohn's disease on ischemic stroke.

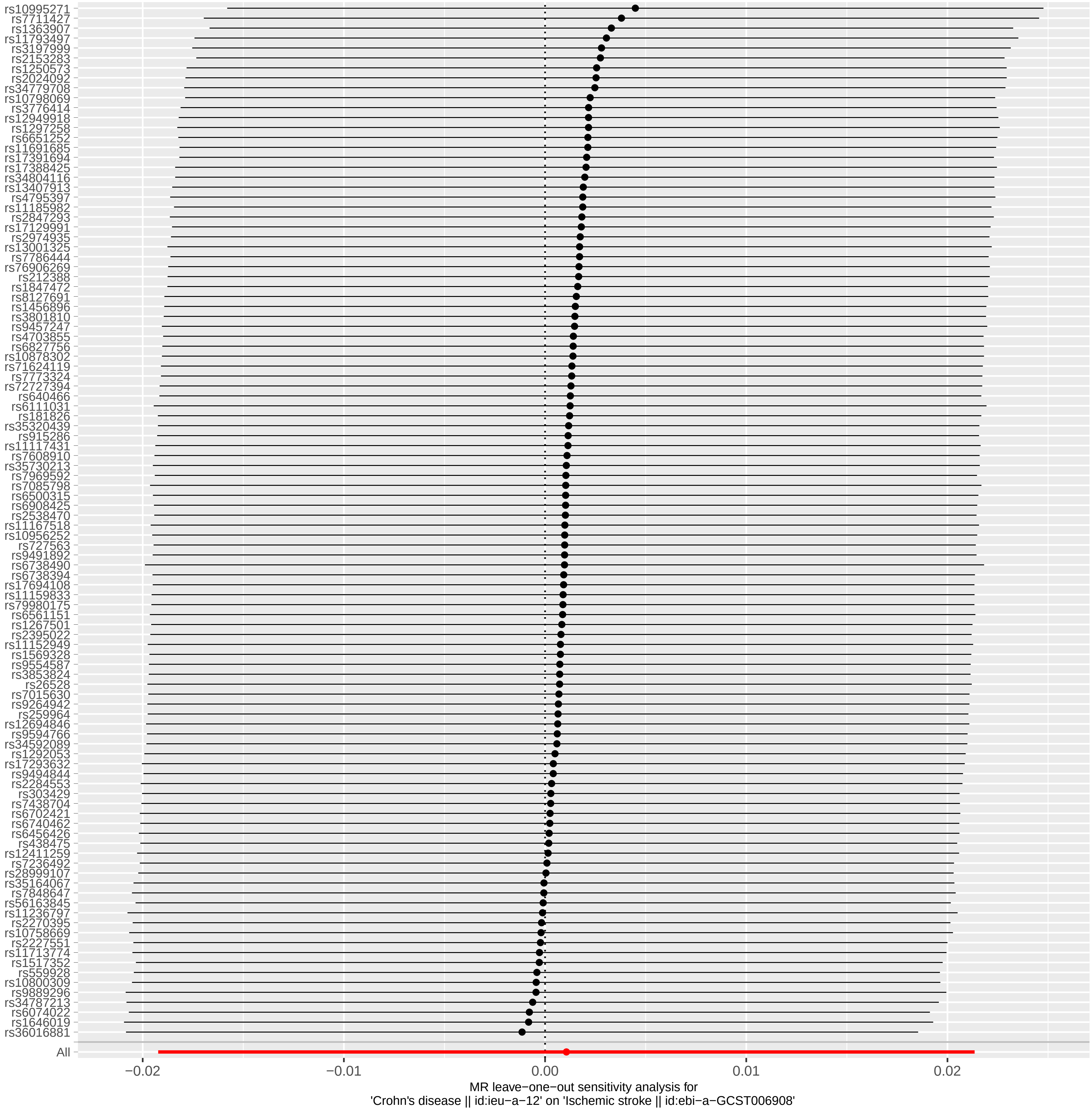

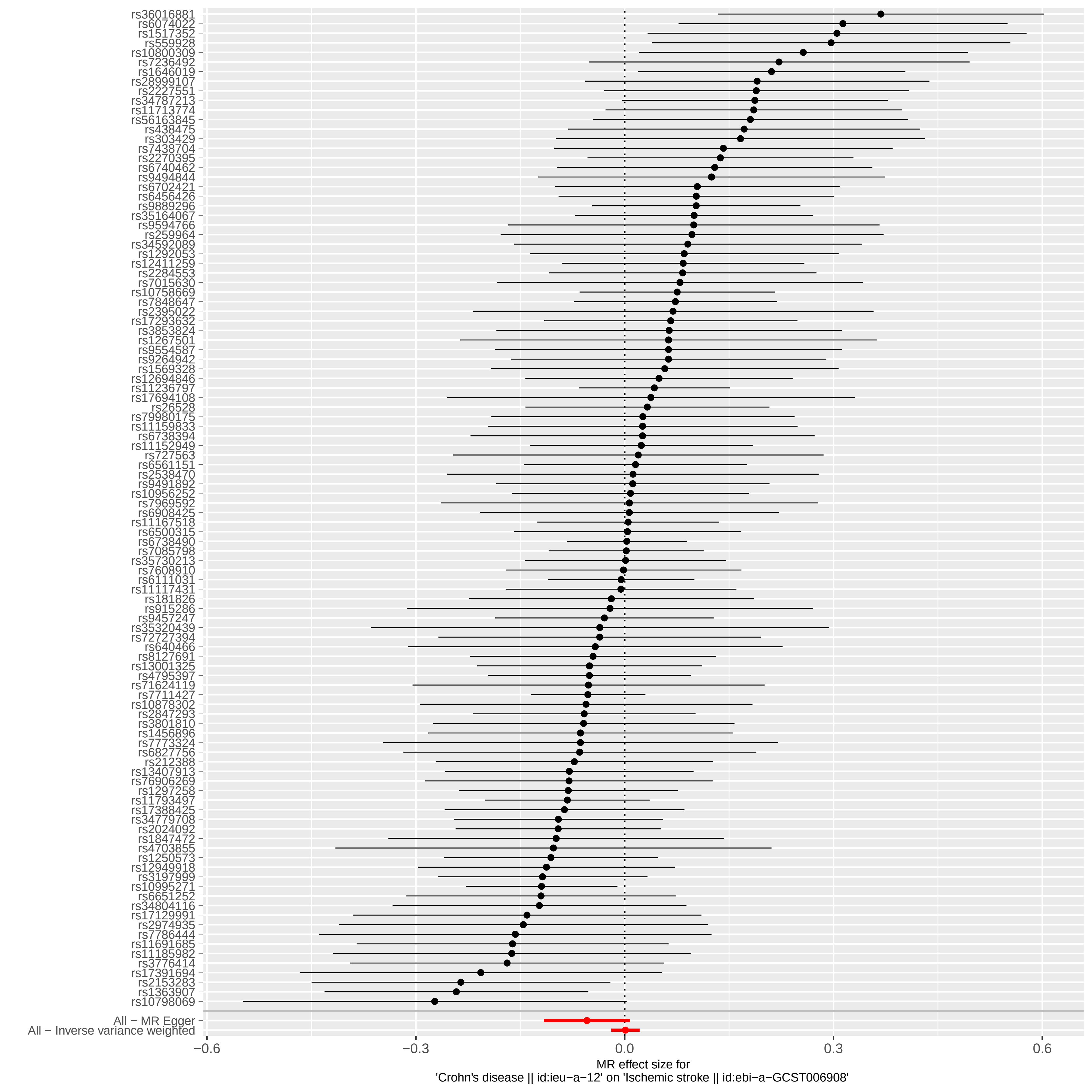
rs36016881
rs6074022
rs1517352
rs559928
rs10800309
rs7236492
rs1646019
rs28999107
rs2227551
rs34787213
rs11713774
rs56163845
rs438475
rs303429
rs7438704
rs2270395
rs6740462
rs9494844
rs6702421
rs6456426
rs9889296
rs35164067
rs9594766
rs259964
rs34592089
rs1292053
rs12411259
rs2284553
rs7015630
rs10758669
rs7848647
rs2395022
rs17293632
rs3853824
rs1267501
rs9554587
rs9264942
rs1569328
rs12694846
rs11236797
rs17694108
rs26528
rs79980175
rs11159833
rs6738394
rs11152949
rs727563
rs6561151
rs2538470
rs9491892
rs10956252
rs7969592
rs6908425
rs11167518
rs6500315
rs6738490
rs7085798
rs35730213
rs7608910
rs6111031
rs11117431
rs181826
rs915286
rs9457247
rs35320439
rs72727394
rs640466
rs8127691
rs13001325
rs4795397
rs71624119
rs7711427
rs10878302
rs2847293
rs3801810
rs1456896
rs7773324
rs6827756
rs212388
rs13407913
rs76906269
rs1297258
rs11793497
rs17388425
rs34779708
rs2024092
rs1847472
rs4703855
rs1250573
rs12949918
rs3197999
rs10995271
rs6651252
rs34804116
rs17129991
rs2974935
rs7786444
rs11691685
rs11185982
rs3776414
rs17391694
rs2153283
rs1363907
rs10798069
All − MR Egger
All − Inverse variance weighted
−0.6
−0.3
0.0
0.3
0.6
MR effect size for
'Crohn's disease || id:ieu−a−12' on 'Ischemic stroke || id:ebi−a−GCST006908'

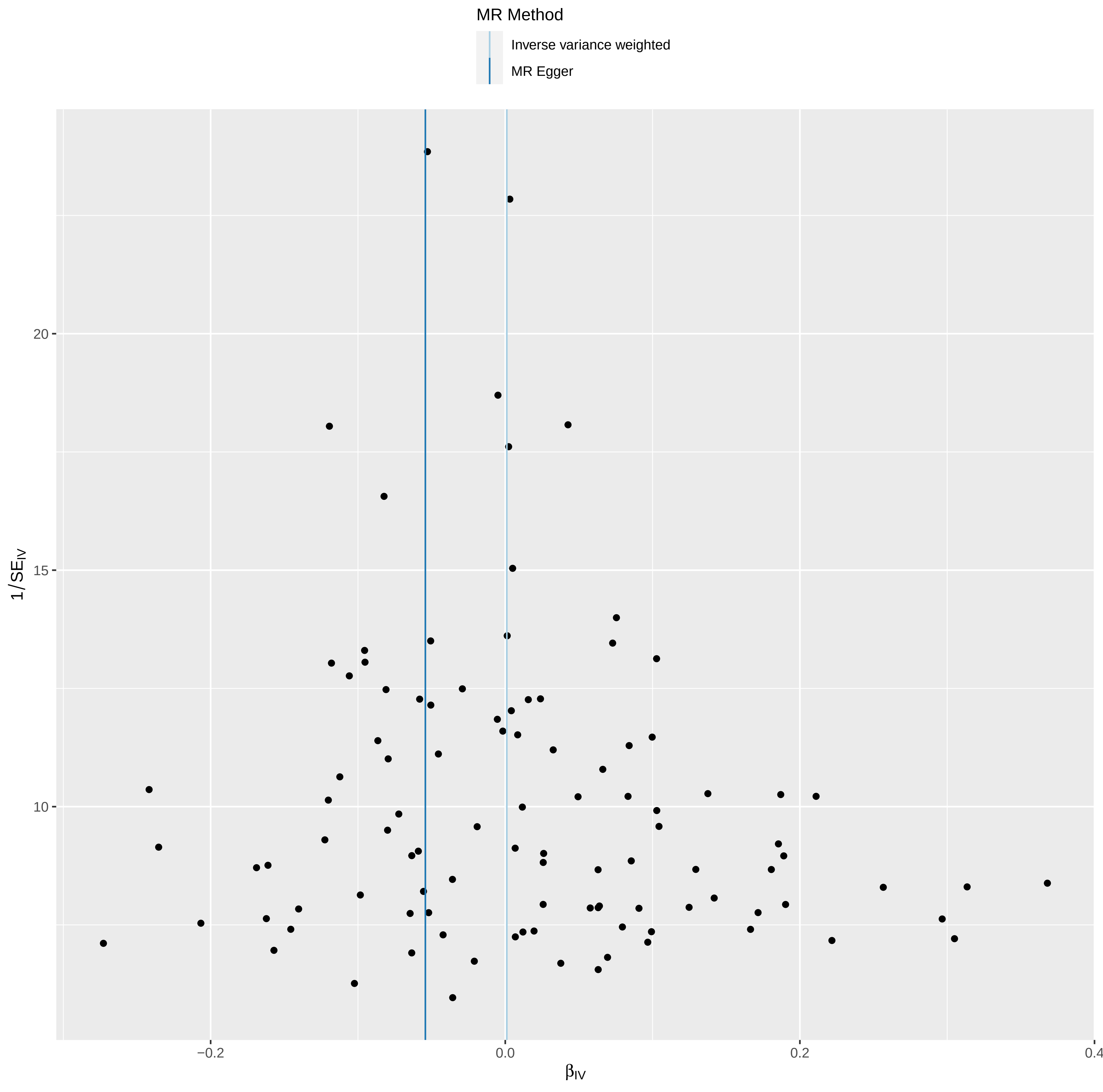

MR Method
Inverse variance weighted
MR Egger
20
15
10
1/SE_IV
−0.2
0.0
0.2
0.4
β_IV

Figure 11 Leave-one-out analysis, MR effect size and funnel plot for Crohn's disease on intracerebral hemorrhage.

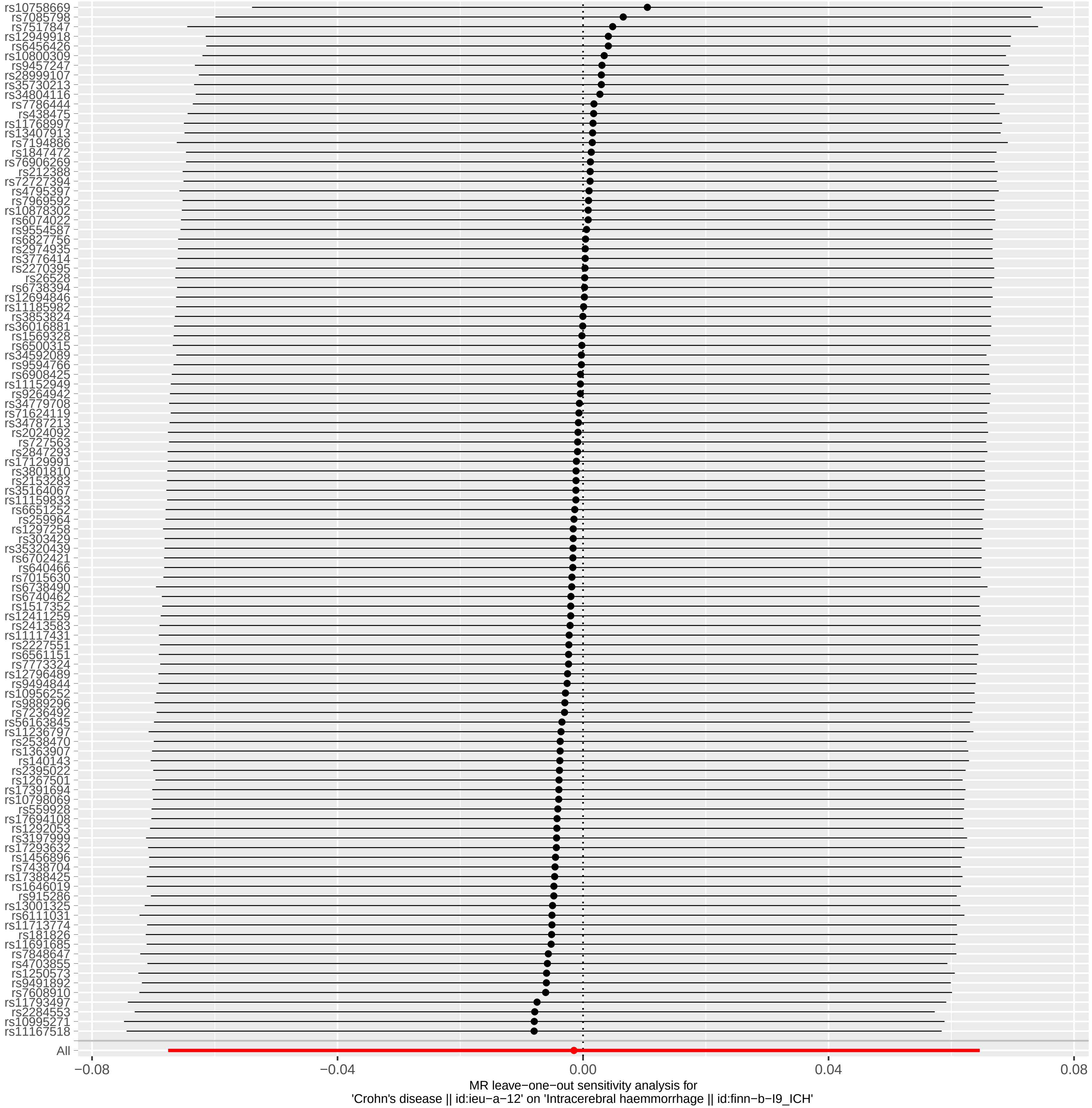

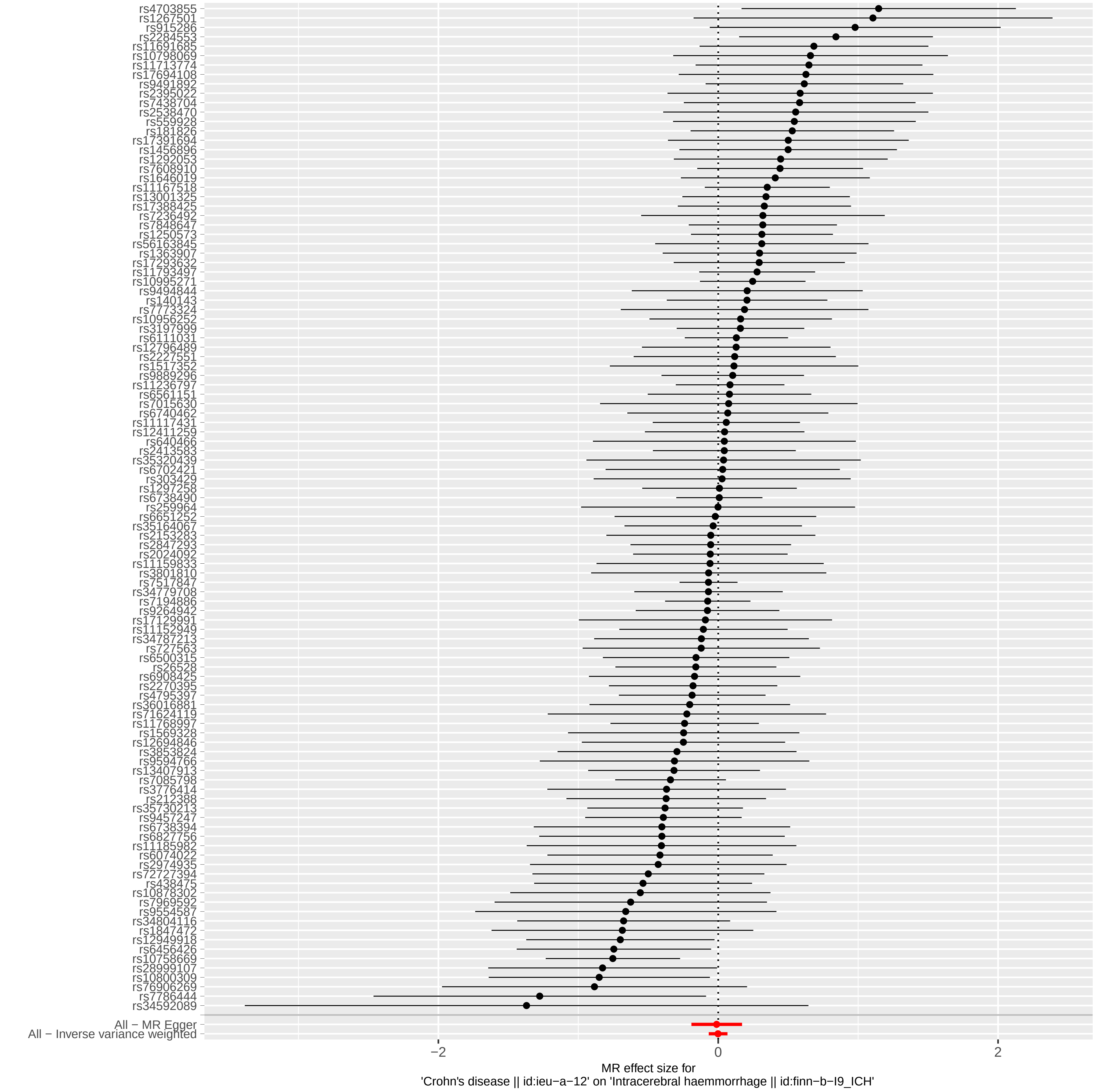
rs4703855
rs1267501
rs915286
rs2284553
rs11691685
rs10798069
rs11713774
rs17694108
rs9491892
rs2395022
rs7438704
rs2538470
rs559928
rs181826
rs17391694
rs1456896
rs1292053
rs7608910
rs1646019
rs11167518
rs13001325
rs17388425
rs7236492
rs7848647
rs1250573
rs56163845
rs1363907
rs17293632
rs11793497
rs10995271
rs9494844
rs140143
rs7773324
rs10956252
rs3197999
rs6111031
rs12796489
rs2227551
rs1517352
rs9889296
rs11236797
rs6561151
rs7015630
rs6740462
rs11117431
rs12411259
rs640466
rs2413583
rs35320439
rs6702421
rs303429
rs1297258
rs6738490
rs259964
rs6651252
rs35164067
rs2153283
rs2847293
rs2024092
rs11159833
rs3801810
rs7517847
rs34779708
rs7194886
rs9264942
rs17129991
rs11152949
rs34787213
rs727563
rs6500315
rs26528
rs6908425
rs2270395
rs4795397
rs36016881
rs71624119
rs11768997
rs1569328
rs12694846
rs3853824
rs9594766
rs13407913
rs7085798
rs3776414
rs212388
rs35730213
rs9457247
rs6738394
rs6827756
rs11185982
rs6074022
rs2974935
rs72727394
rs438475
rs10878302
rs7969592
rs9554587
rs34804116
rs1847472
rs12949918
rs6456426
rs10758669
rs28999107
rs10800309
rs76906269
rs7786444
rs34592089
All − MR Egger
All − Inverse variance weighted
−2
0
2
MR effect size for
'Crohn's disease || id:ieu−a−12' on 'Intracerebral haemmorrhage || id:finn−b−I9_ICH'

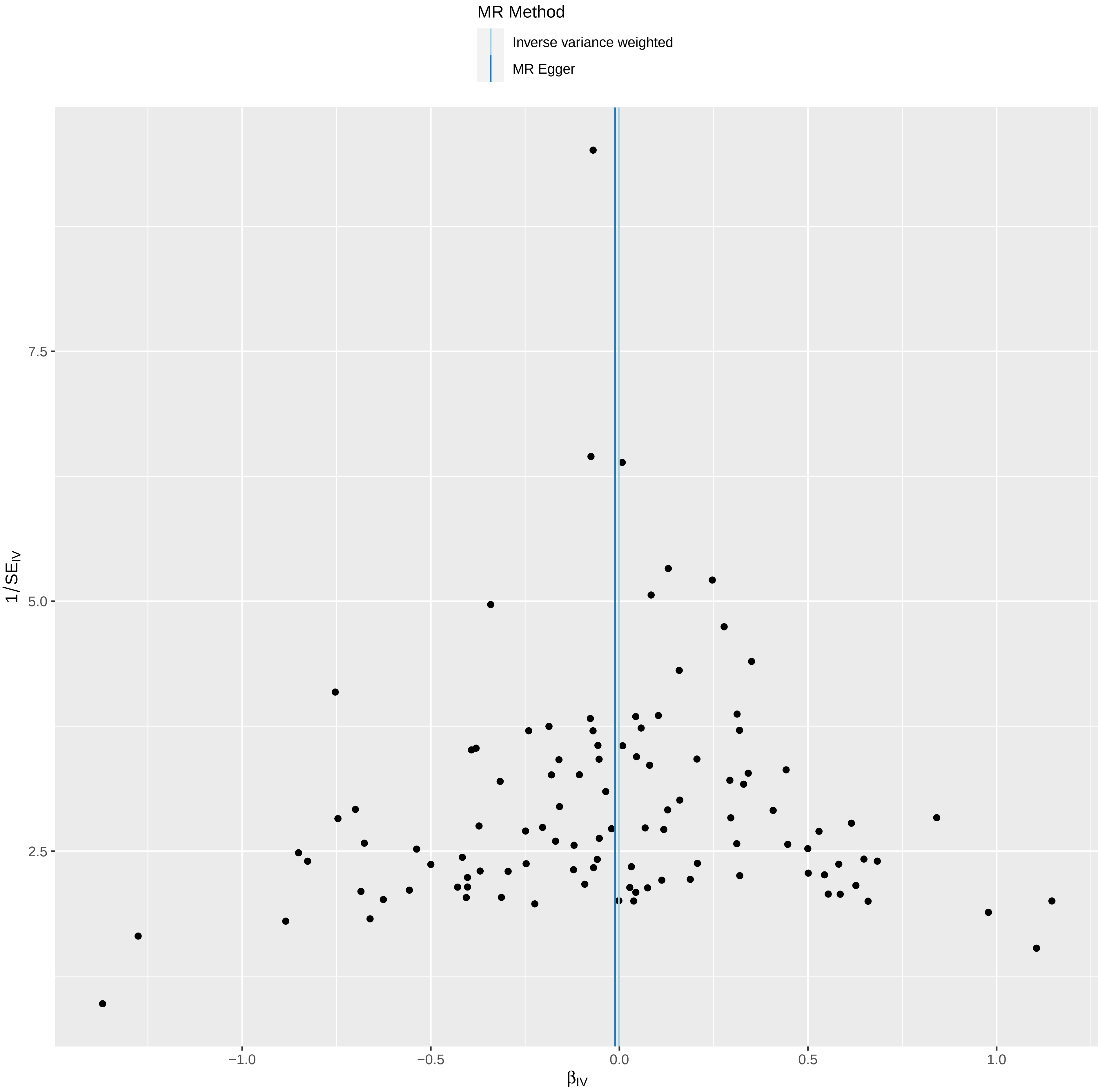

MR Method
Inverse variance weighted
MR Egger
1/SE_IV
7.5
5.0
2.5
−1.0
−0.5
0.0
0.5
1.0
β_IV

Figure 12 Leave-one-out analysis, MR effect size and funnel plot for Crohn's disease on subarachnoid haemmorrhage.

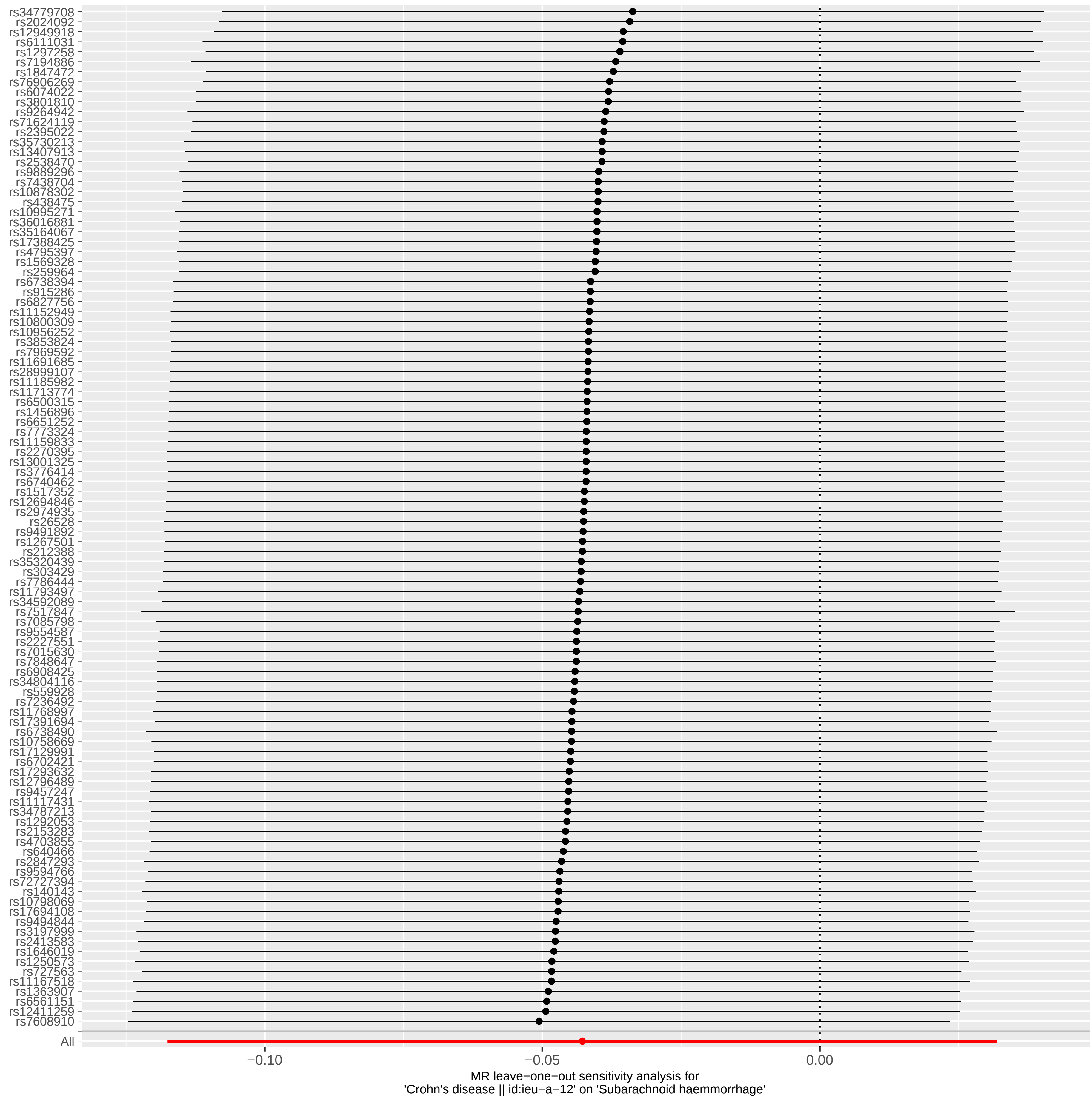

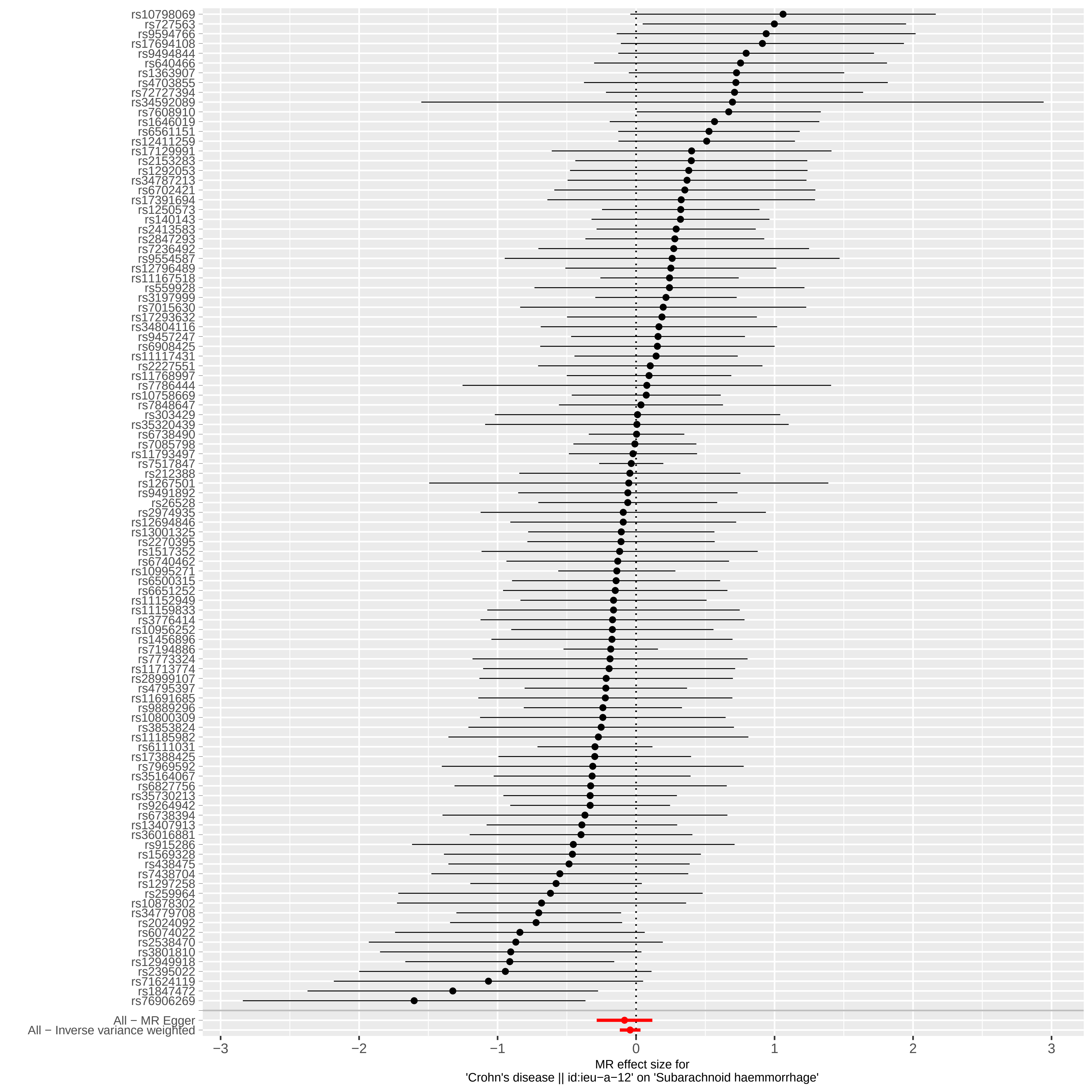

rs10798069
rs727563
rs9594766
rs17694108
rs9494844
rs640466
rs1363907
rs4703855
rs72727394
rs34592089
rs7608910
rs1646019
rs6561151
rs12411259
rs17129991
rs2153283
rs1292053
rs34787213
rs6702421
rs17391694
rs1250573
rs140143
rs2413583
rs2847293
rs7236492
rs9554587
rs12796489
rs11167518
rs559928
rs3197999
rs7015630
rs17293632
rs34804116
rs9457247
rs6908425
rs11117431
rs2227551
rs11768997
rs7786444
rs10758669
rs7848647
rs303429
rs35320439
rs6738490
rs7085798
rs11793497
rs7517847
rs212388
rs1267501
rs9491892
rs26528
rs2974935
rs12694846
rs13001325
rs2270395
rs1517352
rs6740462
rs10995271
rs6500315
rs6651252
rs11152949
rs11159833
rs3776414
rs10956252
rs1456896
rs7194886
rs7773324
rs11713774
rs28999107
rs4795397
rs11691685
rs9889296
rs10800309
rs3853824
rs11185982
rs6111031
rs17388425
rs7969592
rs35164067
rs6827756
rs35730213
rs9264942
rs6738394
rs13407913
rs36016881
rs915286
rs1569328
rs438475
rs7438704
rs1297258
rs259964
rs10878302
rs34779708
rs2024092
rs6074022
rs2538470
rs3801810
rs12949918
rs2395022
rs71624119
rs1847472
rs76906269
All − MR Egger
All − Inverse variance weighted
−3
−2
−1
0
1
2
3
MR effect size for
'Crohn's disease || id:ieu−a−12' on 'Subarachnoid haemmorrhage'

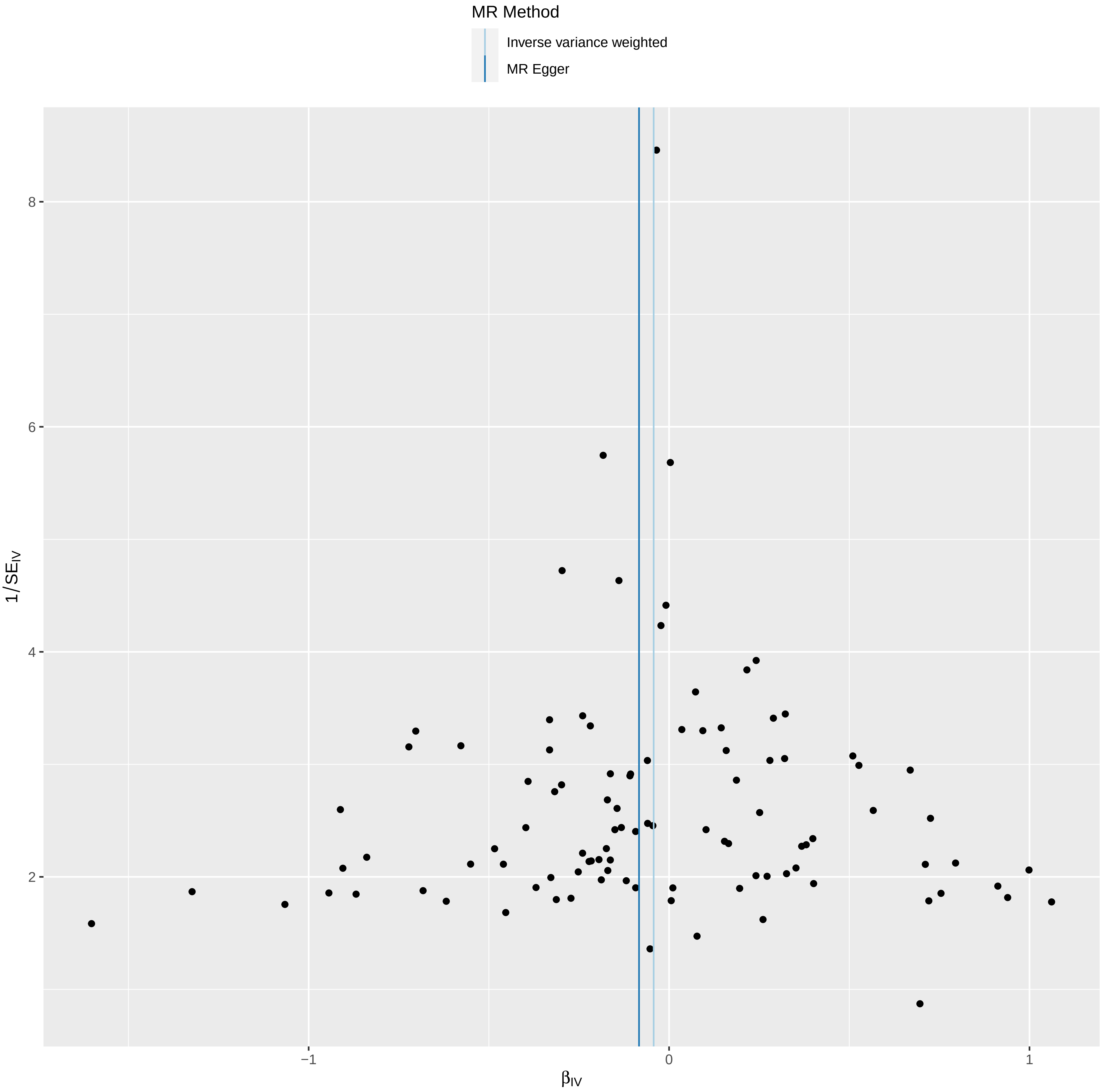

MR Method
Inverse variance weighted
MR Egger
1/SE$_{IV}$
8
6
4
2
−1
0
1
β$_{IV}$

Figure 13 Leave-one-out analysis, MR effect size and funnel plot for Crohn's disease on cerebral infarction.

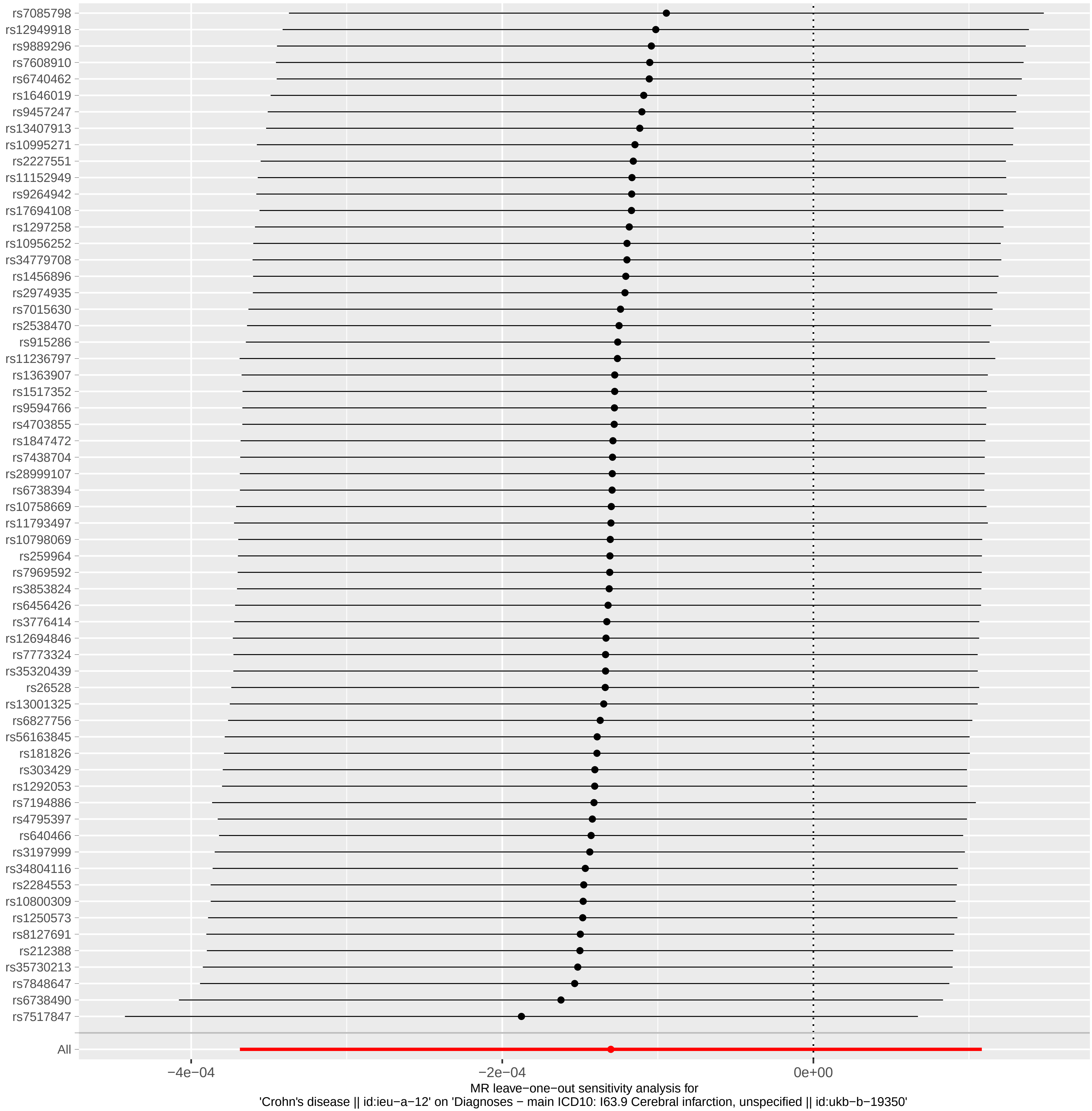

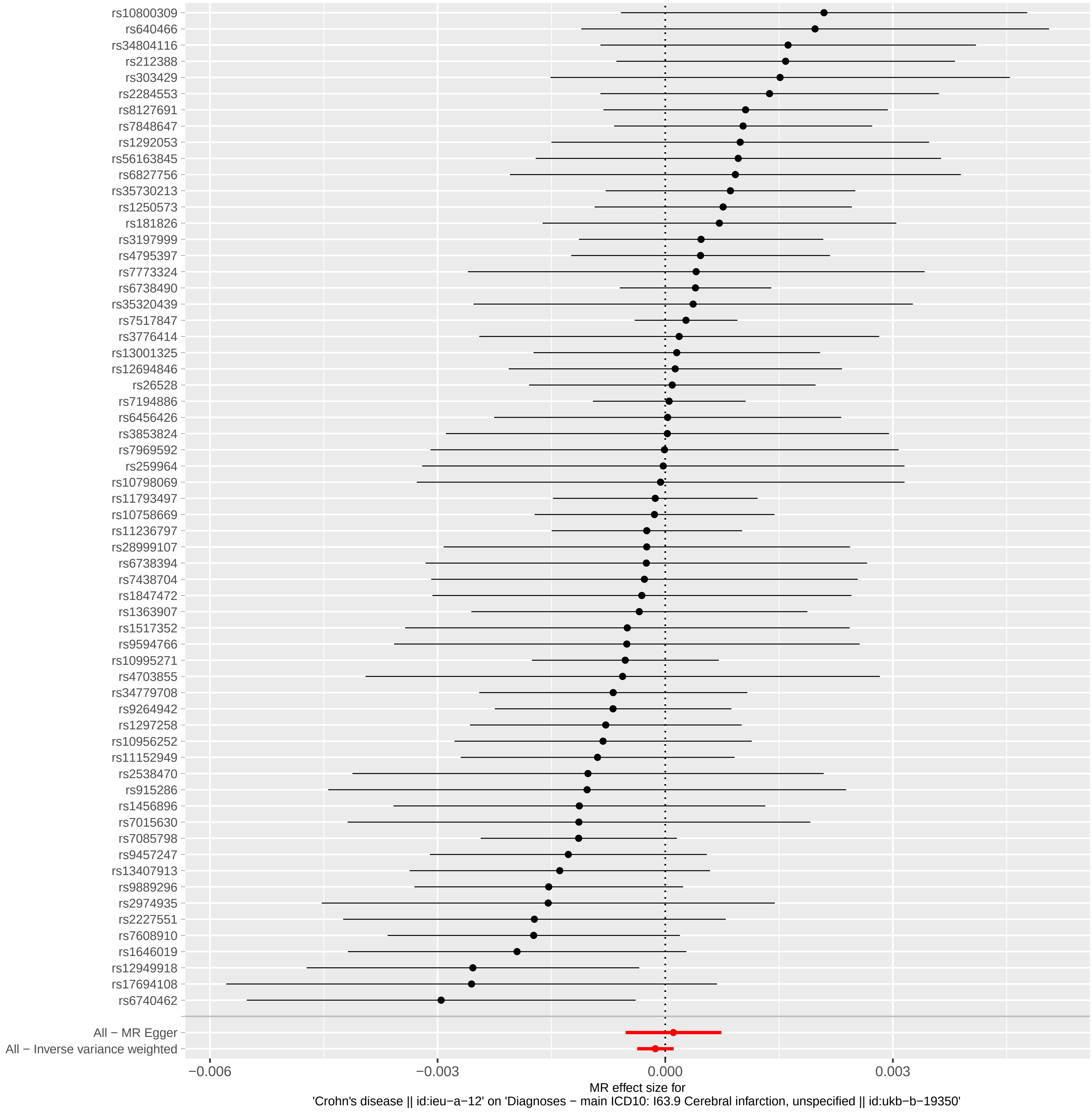
rs10800309
rs640466
rs34804116
rs212388
rs303429
rs2284553
rs8127691
rs7848647
rs1292053
rs56163845
rs6827756
rs35730213
rs1250573
rs181826
rs3197999
rs4795397
rs7773324
rs6738490
rs35320439
rs7517847
rs3776414
rs13001325
rs12694846
rs26528
rs7194886
rs6456426
rs3853824
rs7969592
rs259964
rs10798069
rs11793497
rs10758669
rs11236797
rs28999107
rs6738394
rs7438704
rs1847472
rs1363907
rs1517352
rs9594766
rs10995271
rs4703855
rs34779708
rs9264942
rs1297258
rs10956252
rs11152949
rs2538470
rs915286
rs1456896
rs7015630
rs7085798
rs9457247
rs13407913
rs9889296
rs2974935
rs2227551
rs7608910
rs1646019
rs12949918
rs17694108
rs6740462
All − MR Egger
All − Inverse variance weighted
−0.006
−0.003
0.000
0.003
MR effect size for
'Crohn's disease || id:ieu−a−12' on 'Diagnoses − main ICD10: I63.9 Cerebral infarction, unspecified || id:ukb−b−19350'

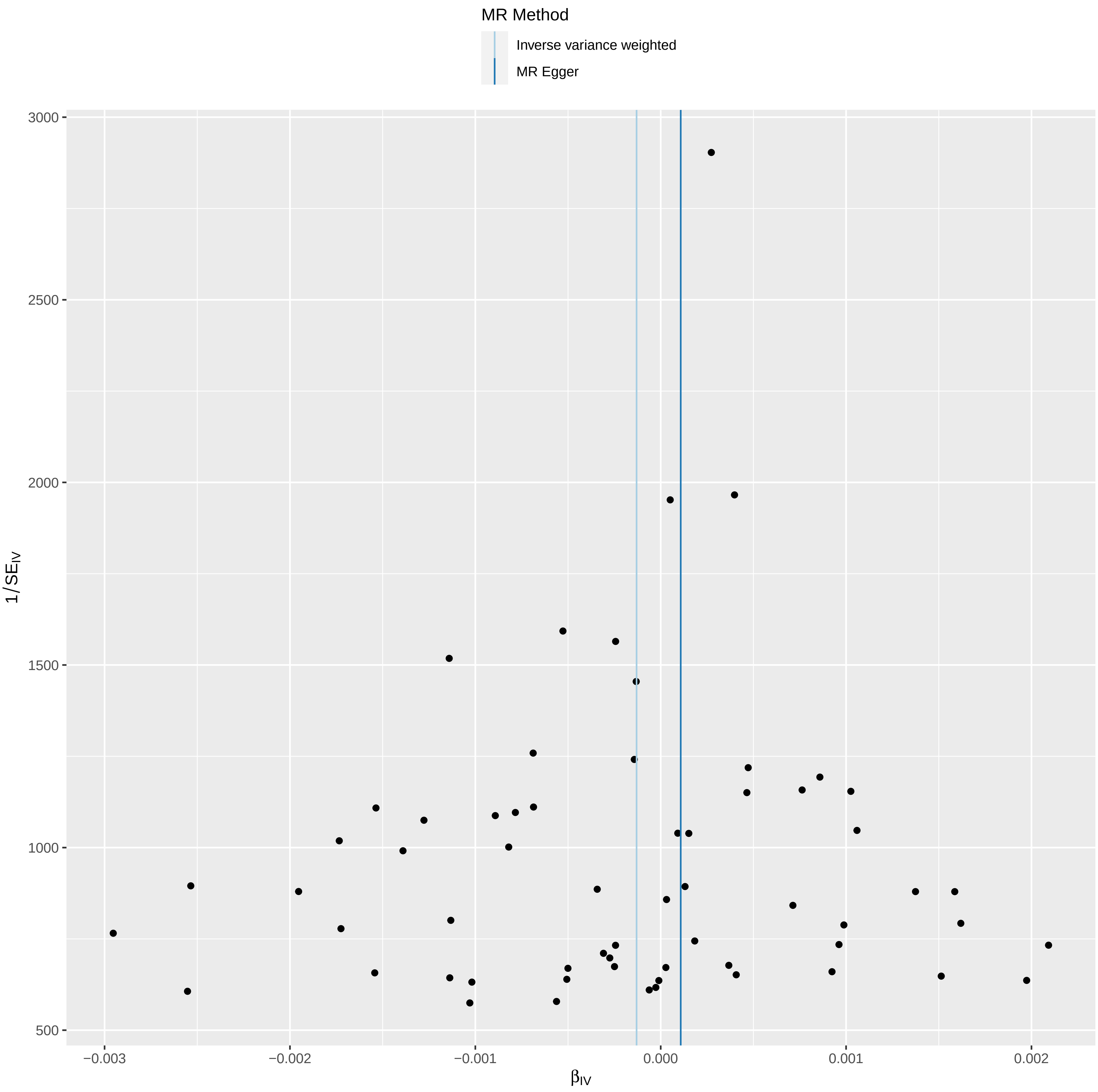
MR Method
Inverse variance weighted
MR Egger
3000
2500
2000
1500
1000
500
1/SE_IV
−0.003
−0.002
−0.001
0.000
0.001
0.002
β_IV

Figure 14 Leave-one-out analysis, MR effect size and funnel plot for Crohn's disease on atherosclerosis.

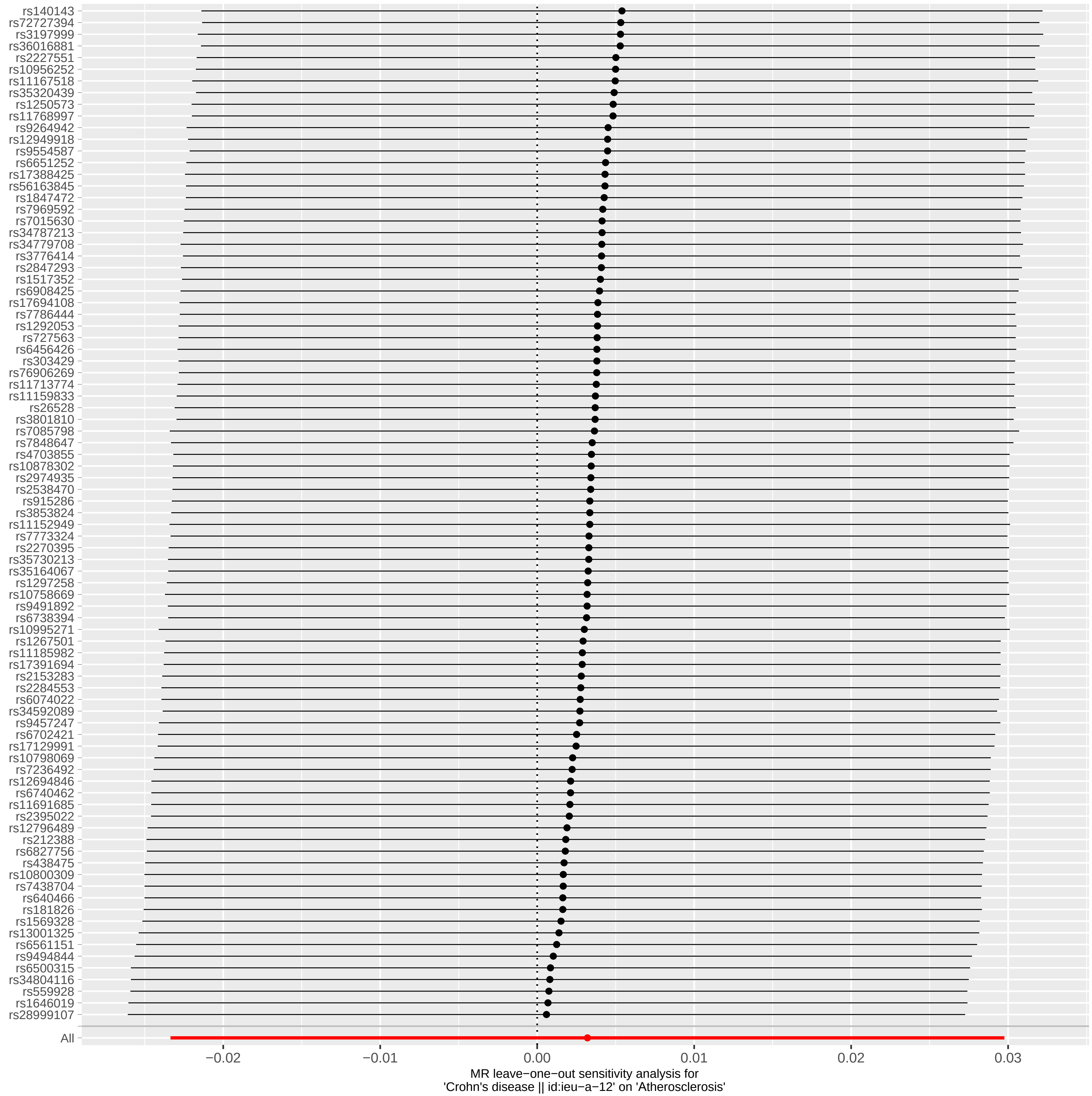

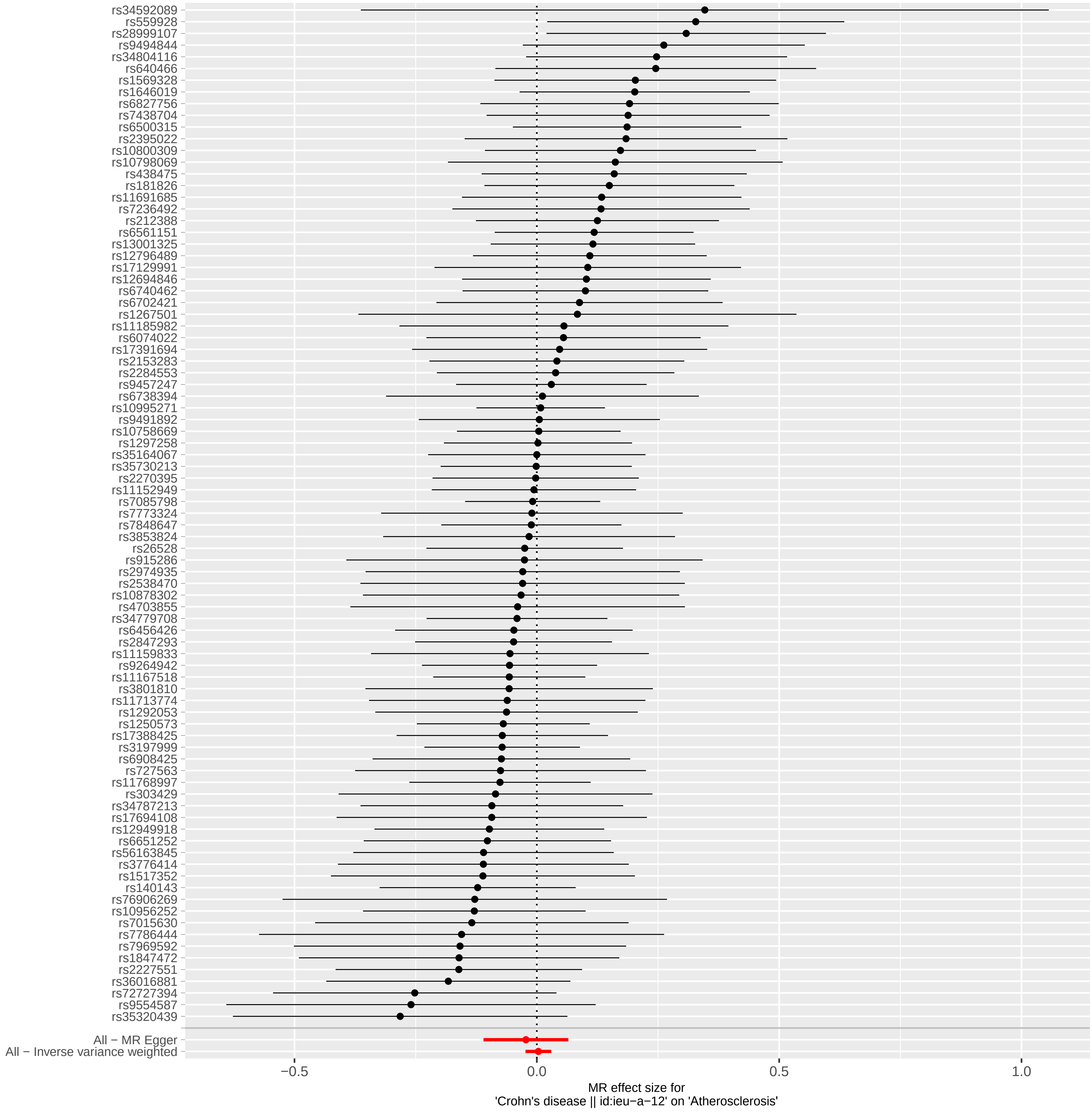
rs34592089
rs559928
rs28999107
rs9494844
rs34804116
rs640466
rs1569328
rs1646019
rs6827756
rs7438704
rs6500315
rs2395022
rs10800309
rs10798069
rs438475
rs181826
rs11691685
rs7236492
rs212388
rs6561151
rs13001325
rs12796489
rs17129991
rs12694846
rs6740462
rs6702421
rs1267501
rs11185982
rs6074022
rs17391694
rs2153283
rs2284553
rs9457247
rs6738394
rs10995271
rs9491892
rs10758669
rs1297258
rs35164067
rs35730213
rs2270395
rs11152949
rs7085798
rs7773324
rs7848647
rs3853824
rs26528
rs915286
rs2974935
rs2538470
rs10878302
rs4703855
rs34779708
rs6456426
rs2847293
rs11159833
rs9264942
rs11167518
rs3801810
rs11713774
rs1292053
rs1250573
rs17388425
rs3197999
rs6908425
rs727563
rs11768997
rs303429
rs34787213
rs17694108
rs12949918
rs6651252
rs56163845
rs3776414
rs1517352
rs140143
rs76906269
rs10956252
rs7015630
rs7786444
rs7969592
rs1847472
rs2227551
rs36016881
rs72727394
rs9554587
rs35320439
All − MR Egger
All − Inverse variance weighted
−0.5
0.0
0.5
1.0
MR effect size for
'Crohn's disease || id:ieu−a−12' on 'Atherosclerosis'

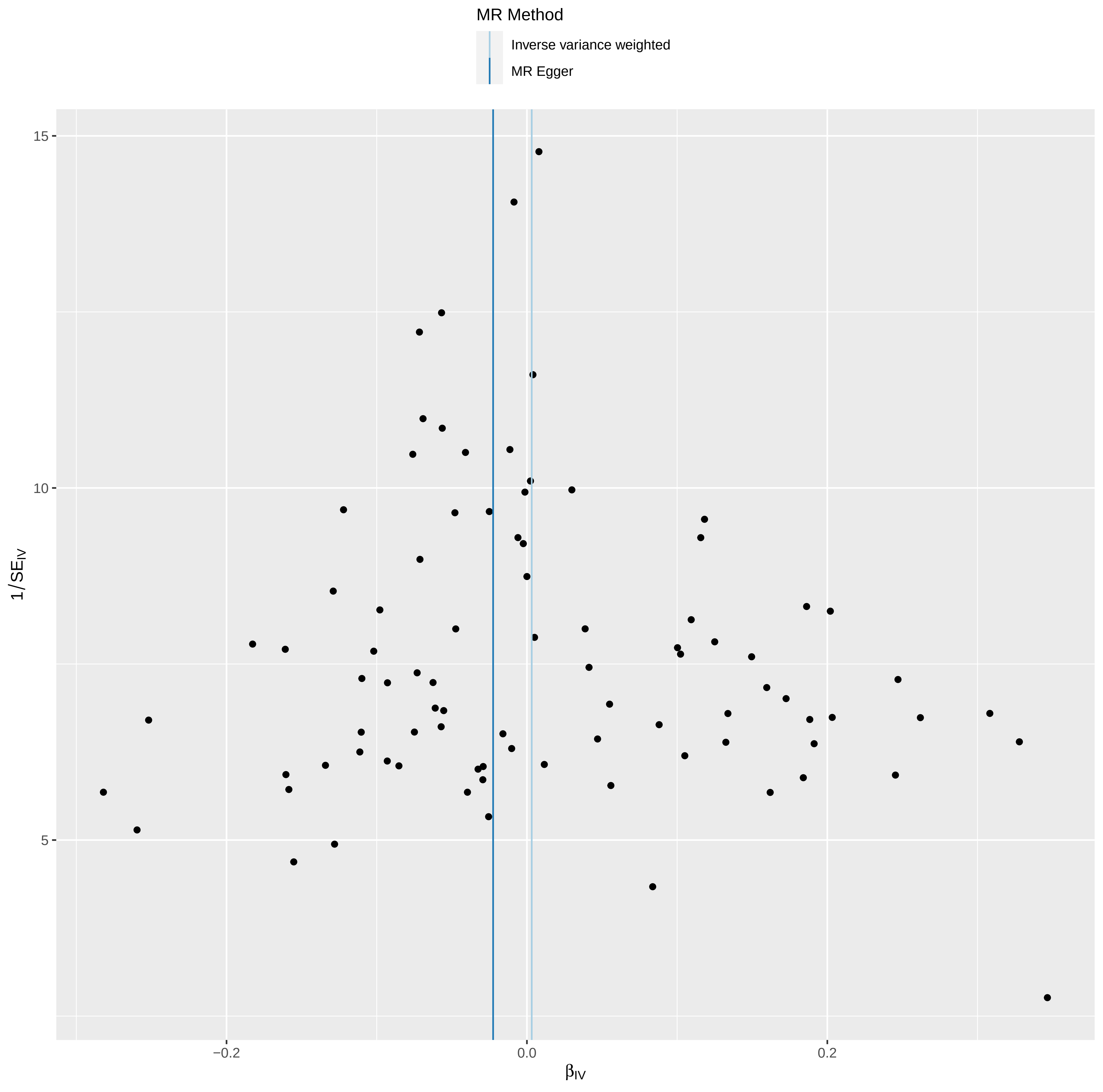

MR Method
Inverse variance weighted
MR Egger
15
10
5
1/SE$_{IV}$
−0.2
0.0
0.2
β$_{IV}$
