# Supplementary material for: Inflammatory bowel disease and cardiovascular disease: A two-sample Mendelian randomization analysis
Source: Front Cardiovasc Med. 2022 Sep 2;9:927120. doi: 10.3389/fcvm.2022.927120 (PMC9478388; doi:10.3389/fcvm.2022.927120)
Supplement: Supplementary material 1 — Instrumental SNPs from ulcerative colitis and Crohn’s disease GWASs. [file Data_Sheet_1.ZIP › Supplementary material 6.pdf]

Code:

1. MR methods

```
library(usethis)
library(devtools)
library(TwoSampleMR)
library(mr.raps)
MRdata <- read.csv(".csv")
exp_dat<- extract_instruments(outcomes="ieu-a-970",    clump=TRUE, access_token=NULL)
#exp_dat <- format_data(MRdata ,type='exposure',snp_col = "rsid",beta_col = "beta",se_col = "se",effect_allele_col ="effect_allele",other_allele_col =
"other_allele",eaf_col = "eaf",pval_col = "pval")
#exp_dath <-clump_data(exp_dat,clump_r2=0.001,clump_kb=10000)
#t2d_out <- extract_outcome_data(snps=exp_dat$SNP, outcomes="ebi-a-GCST90000618", access_token=NULL)
t2d_out <- format_data( dat=MRdata, type = "outcome", snps = exp_dat$SNP, header = TRUE, phenotype_col = "phenotype", snp_col = "rsids", beta_col =
"beta", se_col = "sebeta", effect_allele_col = "ref", other_allele_col = "alt", pval_col = "pval", ncase_col = "N_CASES", ncontrol_col = "N_CONTROLS",
chr_col = "CHROMOSOME", pos_col = "POSITION")
dat <- harmonise_data(exposure_dat=exp_dat, outcome_dat= t2d_out, action = 1)
res <- mr(dat)
```

```
res <- mr.raps(dat$beta.exposure, dat$beta.outcome, dat$se.exposure, dat$se.outcome,    TRUE, "tukey", diagnosis = TRUE)
#or
aaa <- extract_instruments(outcomes='', clump=TRUE, r2=0.001,kb=10000,access_token= NULL )
abc <- extract_outcome_data(
snps= $SNP,
outcomes='',
proxies = FALSE,
maf_threshold = 0.01,
access_token = NULL
)
Mydata <- harmonise_data(
          exposure_dat=,
          outcome_dat=abc,
          action= 2
            )
mr(Mydata, method_list=c("mr_ivw", "mr_ivw_fe", "mr_two_sample_ml", "mr_egger_regression", "mr_weighted_median", "mr_penalised_weighted_median",
"mr_simple_mode", "mr_weighted_mode"))
```

```
library(MRPRESSO)
mr_presso(BetaOutcome ="beta.outcome", BetaExposure = "beta.exposure", SdOutcome ="se.outcome", SdExposure = "se.exposure", OUTLIERtest =
TRUE,DISTORTIONtest = TRUE, data =dat, NbDistribution = 2000, SignifThreshold = 0.05)
mr(dat, method_list=c("mr_egger_regression", "mr_ivw","mr_two_sample_ml","mr_weighted_median","mr_ivw_fe"))
#or
library(MRPRESSO)
write.csv(aaa,file=" ")
write.csv(Mydata,file=" ")
```

2. Sensitivity analyses

2.1 Heterogeneity statistics

```
mr_heterogeneity(Mydata, method_list=c("mr_egger_regression", "mr_ivw"))
```

2.2 Horizontal pleiotropy

```
pleio <- mr_pleiotropy_test(Mydata)
pleio
```

2.3 Single SNP analysis

```
res <- mr(dat)
```

2.4 Leave-one-out analysis

```
single <- mr_leaveoneout(Mydata)
mr_leaveoneout_plot(single)
mr_scatter_plot(res,Mydata)
```

2.5 Forest plot

```
res_single <- mr_singlesnp(Mydata)
mr_forest_plot(res_single)
```

2.6 Funnel plot

```
mr_funnel_plot(res_single)
```
