# Supplementary material for: Inflammatory bowel disease and cardiovascular disease: A two-sample Mendelian randomization analysis
Source: Front Cardiovasc Med. 2022 Sep 2;9:927120. doi: 10.3389/fcvm.2022.927120 (PMC9478388; doi:10.3389/fcvm.2022.927120)
Supplement: Supplementary material 1 — Instrumental SNPs from ulcerative colitis and Crohn’s disease GWASs. [file Data_Sheet_1.ZIP › Supplementary material 7.pdf]

Checklist. STROBE-MR checklist of recommended items to address in reports of Mendelian randomisation studies.

	Item no.	Recommendation	Section (paragraph no.)
**Title and abstract**	1	Indicate Mendelian randomisation (MR) as the study's design in the title and/or the abstract if that is a main purpose of the study	Title, Abstract
**Introduction**			
Background	2	Explain the scientific background and rationale for the reported study. What is the exposure? Is a potential causal relationship between exposure and outcome plausible? Justify why MR is a helpful method to address the study question	Introduction (1,2 and 3)
Objectives	3	State specific objectives clearly, including pre-specified causal hypotheses (if any). State that MR is a method that, under specific assumptions, intends to estimate causal effects	Introduction (4)
**Methods**			
Study design and data sources	4	Present key elements of the study design early in the article. Consider including a table listing sources of data for all phases of the study. For each data source contributing to the analysis, describe the following:	Methods (1, Figure 1 and Data availability statement)
		(a) Setting: Describe the study design and the underlying population, if possible. Describe the setting, locations, and relevant dates, including periods of	Methods (1, Figure 1 and Data availability statement)

		recruitment, exposure, follow-up, and data collection, when available	
		(b) Participants: Give the eligibility criteria, and the sources and methods of selection of participants. Report the sample size, and whether any power or sample size calculations were carried out prior to the main analysis	Methods (1, Figure 1 and Data availability statement)
		(c) Describe measurement, quality control and selection of genetic variants	Methods (2)
		(d) For each exposure, outcome, and other relevant variables, describe methods of assessment and diagnostic criteria for diseases	Methods (1 and 2)
		(e) Provide details of ethics committee approval and participant informed consent, if relevant	NA
Assumptions	5	Explicitly state the three core IV assumptions for the main analysis (relevance, independence, and exclusion restriction) as well assumptions for any additional or sensitivity analysis	Figure 2
Statistical methods: main analysis	6	Describe statistical methods and statistics used	Methods (2,3 and 4)
		(a) Describe how quantitative variables were handled in the analyses (i.e., scale, units, model)	Methods (3)
		(b) Describe how genetic variants were handle]d in the analyses and, if applicable, how their weights were selected	Methods (2,3 and 4)
		(c) Describe the MR estimator (e.g., two-stage least squares, Wald ratio) and related statistics. Detail the included covariates and, in case of two-sample MR,	Methods (3 and 4)

		whether the same covariate set was used for adjustment in the two samples	
		(d) Explain how missing data were addressed	NA
		(e) If applicable, indicate how multiple testing was addressed	NA
Assessment of assumptions	7	Describe any methods or prior knowledge used to assess the assumptions or justify their validity	Methods (4)
Sensitivity analyses and additional analyses	8	Describe any sensitivity analyses or additional analyses performed (e.g., comparison of effect estimates from different approaches, independent replication, bias analytic techniques, validation of instruments, simulations)	Methods (4)
Software and pre-registration	9	(a) Name statistical software and package(s), including version and settings used	Methods (4)
		(b) State whether the study protocol and details were pre-registered (as well as when and where)	NA
**Results**			
Descriptive data	10	(a) Report the numbers of individuals at each stage of included studies and reasons for exclusion.	Results (1)
		(b) Report summary statistics for phenotypic exposure(s), outcome(s), and other relevant variables (e.g., means, SDs, proportions)	Results (1), Figure 1 and Supplementary material 2
		(c) If the data sources include meta-analyses of previous studies, provide the assessments of heterogeneity across these studies	NA

Main results	11	(a) Report the associations between genetic variant and exposure, and between genetic variant and outcome, preferably on an interpretable scale	Results (2), Figure 1 and Supplementary material 2
		(b) Report MR estimates of the relationship between exposure and outcome, and the measures of uncertainty from the MR analysis, on an interpretable scale, such as odds ratio or relative risk per SD difference	Results (2), Figure 1 and Supplementary material 2
		(c) If relevant, consider translating estimates of relative risk into absolute risk for a meaningful time period	Results (2), Figure 1 and Supplementary material 2
		(d) Consider plots to visualize results (e.g., forest plot, scatterplot of associations between genetic variants and outcome versus between genetic variants and exposure)	Supplementary materials 3 and 4
Assessment of assumptions	12	(a) Report the assessment of the validity of the assumptions	Results (3)
		(b) Report any additional statistics (e.g., assessments of heterogeneity across genetic variants, such as I2, Q statistic or E-value)	Supplementary material 5
Sensitivity analyses and additional analyses	13	(a) Report any sensitivity analyses to assess the robustness of the main results to violations of the assumptions	Results (2 and 3)
		(b) Report results from other sensitivity analyses or additional analyses	Results (2 and 3)
		(c) Report any assessment of direction of causal relationship (e.g., bidirectional MR)	NA
		(d) When relevant, report and compare with estimates from non-MR analyses	Disscussion (3)

		(e) Consider additional plots to visualize results (e.g., leave-one-out analyses)	Figure 1
**Discussion**			
Key results	14	Summarise key results with reference to study objectives	Discussion (1)
Limitations	15	Discuss limitations of the study, taking into account the validity of the IV assumptions, other sources of potential bias, and imprecision. Discuss both direction and magnitude of any potential bias and any efforts to address them	Discussion (6)
Interpretation	16	(a) Meaning: Give a cautious overall interpretation of results in the context of their limitations and in comparison with other studies	Discussion (2-6)
		(b) Mechanism: Discuss underlying biological mechanisms that could drive a potential causal relationship between the investigated exposure and the outcome, and whether the gene-environment equivalence assumption is reasonable. Use causal language carefully, clarifying that IV estimates may provide causal effects only under certain assumptions	Discussion (2-55)<br>Conclusion
		(c) Clinical relevance: Discuss whether the results have clinical or public policy relevance, and to what extent they inform effect sizes of possible interventions	Discussion (3,4 and 5)<br>Conclusion
Generalisability	17	Discuss the generalisability of the study results (a) to other populations, (b) across other exposure periods/timings, and (c) across other levels of exposure	Discussion (3,4 and 5)<br>Conclusion

**Other information**			
Funding	18	Describe sources of funding and the role of funders in the present study and, if applicable, sources of funding for the databases and original study or studies on which the present study is based	Data availability statement
Data and data sharing	19	Provide the data used to perform all analyses or report where and how the data can be accessed, and reference these sources in the article. Provide the statistical code needed to reproduce the results in the article, or report whether the code is publicly accessible and if so, where	Methods (1), Data availability statement
Conflicts of interest	20	All authors should declare all potential conflicts of interest	No
