# Supplementary material for: Inflammatory bowel disease and cardiovascular disease: A two-sample Mendelian randomization analysis
Source: Front Cardiovasc Med. 2022 Sep 2;9:927120. doi: 10.3389/fcvm.2022.927120 (PMC9478388; doi:10.3389/fcvm.2022.927120)
Supplement: Supplementary material 1 — Instrumental SNPs from ulcerative colitis and Crohn’s disease GWASs. [file Data_Sheet_1.ZIP › Supplementary material 1.docx]

Supplementary material 1: Instrumental SNPs from ulcerative colitis and Crohn's disease GWASs.

1. Instrumental SNPs from ulcerative colitis GWASs.

| SNP | Gene | Chr | POS | Effect Allele | Other Allele | EAF | β | SE | P value | R^2^ | F statistic |
| --- | --- | --- | --- | --- | --- | --- | --- | --- | --- | --- | --- |
| rs10910092 | TNFRSF14 | 1 | 2501516 | G | A | 0.468 | -0.086 | 0.013 | 1.42E-11 | 0.0113 | 134.8081 |
| rs111830527 | RP11-415K20.2 | 1 | 22687173 | A | G | 0.053 | -0.192 | 0.029 | 5.09E-11 | 0.0136 | 191.6034 |
| rs12103 | INTS11 | 1 | 1247494 | C | T | 0.817 | -0.100 | 0.016 | 9.96E-10 | 0.0037 | 43.1433 |
| rs12132349 | C1orf106 | 1 | 200875242 | A | T | 0.281 | -0.167 | 0.014 | 3.64E-31 | 0.0306 | 288.8827 |
| rs16841904 | DENND1B | 1 | 197701992 | T | C | 0.203 | 0.086 | 0.015 | 1.90E-08 | 0.003 | 37.3321 |
| rs1801274 | FCGR2A | 1 | 161479745 | G | A | 0.496 | -0.171 | 0.013 | 1.43E-41 | 0.0037 | 45.638 |
| rs3024493 | IL10 | 1 | 206943968 | A | C | 0.157 | 0.226 | 0.016 | 1.42E-43 | 0.0058 | 64.3557 |
| rs35223180 | RP11-431K24.1 | 1 | 8185902 | T | G | 0.179 | -0.141 | 0.018 | 1.04E-15 | 0.0269 | 341.0467 |
| rs4656958 | ITLN1 | 1 | 160856964 | G | A | 0.682 | 0.082 | 0.014 | 2.82E-09 | 0.0029 | 35.3066 |
| rs6426833 | RP11-91K11.2 | 1 | 20171860 | A | G | 0.536 | 0.232 | 0.013 | 3.77E-76 | 0.0146 | 182.4218 |
| rs7547569 | RNU4ATAC4P | 1 | 67731368 | C | T | 0.067 | -0.496 | 0.029 | 8.71E-65 | 0.0024 | 31.5906 |
| rs10185424 | IL1R2 | 2 | 102662888 | G | T | 0.540 | -0.097 | 0.013 | 1.47E-14 | 0.0077 | 100.3914 |
| rs10460566 | DNMT3A | 2 | 25483121 | A | G | 0.761 | -0.082 | 0.014 | 1.60E-08 | 0.0035 | 40.6936 |
| rs11676348 | SERPINE2 | 2 | 219010146 | T | C | 0.476 | 0.074 | 0.012 | 2.08E-09 | 0.0024 | 32.195 |
| rs13430791 | THADA | 2 | 43481013 | A | G | 0.120 | 0.106 | 0.019 | 1.39E-08 | 0.0047 | 58.3869 |
| rs1517352 | STAT4 | 2 | 191931464 | C | A | 0.605 | 0.078 | 0.013 | 2.10E-09 | 0.0024 | 31.9322 |
| rs1990760 | IFIH1 | 2 | 163124051 | T | C | 0.609 | -0.086 | 0.013 | 1.78E-10 | 0.0024 | 31.2631 |
| rs4676410 | GPR35 | 2 | 241563739 | A | G | 0.204 | 0.142 | 0.016 | 1.85E-19 | 0.0046 | 59.1324 |
| rs4973341 | NEU2 | 2 | 228660362 | T | C | 0.663 | 0.073 | 0.013 | 2.25E-08 | 0.0029 | 35.8786 |
| rs7608910 | PUS10 | 2 | 61204856 | G | A | 0.391 | 0.127 | 0.013 | 1.25E-23 | 0.0065 | 81.3876 |
| rs9941524 | AC019330.1 | 2 | 199499443 | G | A | 0.456 | 0.098 | 0.013 | 2.15E-14 | 0.0028 | 35.9006 |
| rs9836291 | BSN | 3 | 49697459 | A | G | 0.288 | 0.170 | 0.013 | 8.20E-38 | 0.0119 | 165.2166 |
| rs13136827 | KIAA1109 | 4 | 123171318 | C | T | 0.162 | -0.112 | 0.018 | 2.35E-10 | 0.0034 | 40.155 |
| rs3774937 | NFKB1 | 4 | 103434253 | C | T | 0.326 | 0.099 | 0.013 | 4.61E-14 | 0.0043 | 56.8903 |
| rs272882 | SLC22A4 | 5 | 131669161 | T | G | 0.673 | 0.146 | 0.014 | 6.67E-26 | 0.0089 | 115.156 |
| rs36070529 | RNF145 | 5 | 158619835 | A | G | 0.200 | -0.092 | 0.016 | 1.04E-08 | 0.0094 | 110.7637 |
| rs3776414 | DAP | 5 | 10689562 | G | T | 0.376 | 0.070 | 0.013 | 4.10E-08 | 0.0027 | 32.7636 |
| rs4976646 | RGS14 | 5 | 176788570 | C | T | 0.342 | 0.079 | 0.013 | 2.52E-09 | 0.0028 | 35.5256 |
| rs56167332 | AC008697.1 | 5 | 158827769 | A | C | 0.338 | 0.141 | 0.013 | 7.27E-27 | 0.0023 | 30.1 |
| rs7711427 | RP11-386E5.1 | 5 | 40414886 | C | A | 0.613 | 0.089 | 0.013 | 3.67E-12 | 0.0038 | 48.2914 |
| rs2516440 | HCP5 | 6 | 31440497 | A | G | 0.322 | -0.100 | 0.014 | 4.40E-13 | 0.0069 | 116.6019 |
| rs34659678 | TRAF3IP2 | 6 | 111888540 | T | C | 0.057 | 0.210 | 0.025 | 5.95E-17 | 0.0026 | 39.44 |
| rs4712520 | CDKAL1 | 6 | 20640871 | C | T | 0.818 | 0.093 | 0.017 | 2.21E-08 | 0.0075 | 89.9703 |
| rs4947328 | NCR3 | 6 | 31561747 | G | A | 0.024 | 0.239 | 0.038 | 3.38E-10 | 0.0026 | 31.302 |
| rs6920220 | RP11-95M15.1 | 6 | 138006504 | A | G | 0.209 | 0.147 | 0.015 | 4.78E-22 | 0.0043 | 52.4542 |
| rs7738430 | DDX39B | 6 | 31508836 | C | T | 0.026 | 0.368 | 0.034 | 3.51E-27 | 0.0319 | 425.834 |
| rs9271255 | TBC1D22B | 6 | 32580357 | T | C | 0.732 | -0.285 | 0.014 | 1.31E-94 | 0.0048 | 69.9926 |
| rs9271858 | - | 6 | 32595223 | G | A | 0.511 | 0.123 | 0.013 | 2.42E-21 | 0.0071 | 93.1779 |
| rs1077773 | RP11-507K12.1 | 7 | 17442679 | A | G | 0.524 | 0.072 | 0.012 | 5.96E-09 | 0.0026 | 33.846 |
| rs1182188 | GNA12 | 7 | 2869985 | C | T | 0.299 | -0.108 | 0.014 | 5.03E-15 | 0.0025 | 32.1715 |
| rs12718244 | C7orf72 | 7 | 50175654 | A | G | 0.408 | 0.072 | 0.013 | 1.41E-08 | 0.0049 | 61.2486 |
| rs2395022 | SMURF1 | 7 | 98750379 | C | A | 0.959 | -0.184 | 0.029 | 2.88E-10 | 0.0027 | 39.7549 |
| rs4728142 | IRF5 | 7 | 128573967 | A | G | 0.439 | 0.097 | 0.013 | 1.92E-14 | 0.0046 | 58.6144 |
| rs6466198 | PIGCP2 | 7 | 107480126 | T | A | 0.386 | 0.134 | 0.013 | 1.90E-25 | 0.0025 | 41.7277 |
| rs76546301 | RNU6-1091P | 7 | 50498389 | A | G | 0.018 | 0.265 | 0.041 | 1.05E-10 | 0.0085 | 108.6913 |
| rs13255292 | PVT1 | 8 | 129076573 | T | C | 0.328 | -0.075 | 0.014 | 3.82E-08 | 0.0025 | 30.2414 |
| rs10758669 | JAK2 | 9 | 4981602 | A | C | 0.650 | -0.143 | 0.013 | 1.04E-28 | 0.0063 | 78.5515 |
| rs10870077 | CARD9 | 9 | 139263891 | G | C | 0.572 | -0.136 | 0.013 | 5.77E-27 | 0.009 | 115.615 |
| rs4366152 | TNFSF15 | 9 | 117564875 | C | T | 0.680 | 0.120 | 0.014 | 7.79E-19 | 0.0093 | 123.5875 |
| rs4743820 | LINC00484 | 9 | 93928416 | T | C | 0.702 | 0.081 | 0.014 | 4.05E-09 | 0.0027 | 34.6004 |
| rs10748783 | RP11-129J12.1 | 10 | 101285872 | A | C | 0.524 | -0.165 | 0.013 | 7.73E-39 | 0.0135 | 169.9142 |
| rs10761659 | ZNF365 | 10 | 64445564 | G | A | 0.540 | 0.117 | 0.013 | 1.50E-20 | 0.0025 | 29.7576 |
| rs2274351 | SUFU | 10 | 104264107 | T | C | 0.537 | 0.071 | 0.013 | 4.90E-08 | 0.0025 | 32.5757 |
| rs2497318 | EIF2S2P3 | 10 | 94432000 | T | C | 0.450 | -0.071 | 0.013 | 1.15E-08 | 0.0026 | 32.924 |
| rs4747886 | PFKFB3 | 10 | 6176166 | T | C | 0.408 | 0.074 | 0.013 | 9.58E-09 | 0.0025 | 32.1192 |
| rs59418206 | CUL2 | 10 | 35331624 | A | G | 0.351 | 0.074 | 0.013 | 1.45E-08 | 0.0068 | 86.3628 |
| rs11229555 | GLYAT | 11 | 58408687 | T | G | 0.252 | -0.082 | 0.014 | 1.21E-08 | 0.0204 | 146.1274 |
| rs11230563 | CD6 | 11 | 60776209 | T | C | 0.348 | -0.075 | 0.013 | 1.90E-08 | 0.0072 | 93.2531 |
| rs12796489 | CARS | 11 | 3059360 | A | C | 0.023 | -0.676 | 0.056 | 1.22E-33 | 0.0026 | 32.4633 |
| rs483905 | MAML2 | 11 | 96023427 | A | G | 0.289 | 0.085 | 0.014 | 3.16E-10 | 0.0026 | 31.5912 |
| rs61893460 | RP11-672A2.7 | 11 | 76291154 | A | G | 0.445 | 0.121 | 0.013 | 4.60E-22 | 0.007 | 84.8728 |
| rs661054 | NXPE1 | 11 | 114430410 | G | A | 0.341 | -0.125 | 0.014 | 3.18E-20 | 0.003 | 39.5738 |
| rs12318183 | IFNG-AS1 | 12 | 68503836 | A | C | 0.385 | 0.162 | 0.013 | 1.44E-37 | 0.0125 | 164.0926 |
| rs76904798 | LRRK2 | 12 | 40614434 | T | C | 0.137 | 0.105 | 0.018 | 2.78E-09 | 0.0026 | 35.3324 |
| rs1927681 | RP11-545M8.4 | 13 | 27558881 | A | T | 0.443 | -0.479 | 0.013 | 1.00E-200 | 0.0044 | 54.7183 |
| rs941823 | LINC00598 | 13 | 41013977 | C | T | 0.751 | 0.109 | 0.015 | 1.39E-13 | 0.1131 | 1429.644 |
| rs55808324 | GALC | 14 | 88444752 | A | G | 0.093 | 0.127 | 0.021 | 1.47E-09 | 0.0027 | 36.5722 |
| rs11150589 | ITGAL | 16 | 30482494 | C | T | 0.527 | -0.080 | 0.013 | 3.28E-10 | 0.0025 | 32.0223 |
| rs11641184 | LITAF | 16 | 11704651 | A | C | 0.476 | 0.078 | 0.012 | 4.24E-10 | 0.0026 | 32.1418 |
| rs7404095 | PRKCB | 16 | 23864590 | C | T | 0.580 | 0.072 | 0.013 | 1.52E-08 | 0.0032 | 39.498 |
| rs79045992 | RPL35AP33 | 16 | 68518992 | A | G | 0.103 | 0.118 | 0.021 | 1.43E-08 | 0.003 | 38.9988 |
| rs17780256 | SLC39A11 | 17 | 70642923 | C | A | 0.193 | -0.115 | 0.016 | 6.13E-13 | 0.0097 | 123.6487 |
| rs4795397 | IKZF3 | 17 | 38023745 | G | A | 0.471 | 0.140 | 0.013 | 1.01E-28 | 0.0037 | 45.2623 |
| rs9891119 | STAT3 | 17 | 40507980 | C | A | 0.354 | -0.090 | 0.013 | 1.72E-11 | 0.0041 | 51.8054 |
| rs7240004 | CTIF | 18 | 46395022 | G | A | 0.380 | -0.082 | 0.013 | 2.50E-10 | 0.0032 | 40.0321 |
| rs8096327 | PTPN2 | 18 | 12887750 | G | A | 0.384 | 0.094 | 0.013 | 2.24E-13 | 0.0042 | 53.7805 |
| rs11083840 | PTGIR | 19 | 47119910 | G | T | 0.403 | 0.069 | 0.013 | 3.41E-08 | 0.0037 | 47.2751 |
| rs12720356 | TYK2 | 19 | 10469975 | C | A | 0.086 | 0.153 | 0.023 | 1.67E-11 | 0.0037 | 45.3215 |
| rs17694108 | SLC7A10 | 19 | 33731551 | A | G | 0.280 | 0.096 | 0.014 | 6.17E-12 | 0.0023 | 30.4589 |
| rs4812833 | LINC01620 | 20 | 43068996 | A | G | 0.519 | 0.103 | 0.013 | 1.87E-16 | 0.0182 | 187.1509 |
| rs6062496 | TNFRSF6B | 20 | 62329099 | A | G | 0.569 | 0.114 | 0.013 | 9.14E-19 | 0.0064 | 78.2365 |
| rs6111031 | SIRPB3P | 20 | 1682037 | T | C | 0.159 | -0.261 | 0.019 | 1.33E-42 | 0.0025 | 32.4405 |
| rs913678 | RP13-379L11.3 | 20 | 48955424 | C | T | 0.329 | -0.076 | 0.013 | 1.23E-08 | 0.0053 | 67.7343 |
| rs1297256 | AJ006998.2 | 21 | 16805676 | T | C | 0.425 | -0.101 | 0.013 | 2.10E-15 | 0.005 | 62.9729 |
| rs2836883 | PCP4 | 21 | 40466744 | A | G | 0.273 | -0.227 | 0.015 | 1.47E-53 | 0.005 | 65.114 |
| rs4456788 | AP001057.1 | 21 | 45616324 | A | G | 0.611 | -0.103 | 0.013 | 7.07E-16 | 0.0205 | 237.3711 |
| rs140143 | - | 22 | 30173109 | T | G | 0.390 | -0.130 | 0.014 | 1.19E-19 | 0.0051 | 61.779 |
| rs9611131 | SCUBE1 | 22 | 39662480 | C | T | 0.148 | -0.143 | 0.018 | 3.84E-15 | 0.008 | 82.2713 |

(2) Instrumental SNPs from Crohn's disease GWASs.

| SNP | Gene | Chr | POS | Effect allele | Other allele | EAF | β | SE | P value | R^2^ | F statistic |
| --- | --- | --- | --- | --- | --- | --- | --- | --- | --- | --- | --- |
| rs10798069 | PLA2G4A | 1 | 186875459 | T | G | 0.493 | -0.070 | 0.012 | 4.25E-09 | 0.0036 | 39.8000 |
| rs10800309 | FCGR2A | 1 | 161472158 | G | A | 0.658 | -0.090 | 0.013 | 8.48E-13 | 0.0030 | 35.4492 |
| rs12411259 | RP1-15D23.2 | 1 | 172866210 | A | G | 0.240 | 0.134 | 0.014 | 1.43E-22 | 0.0029 | 38.3889 |
| rs17129991 | IL12RB2 | 1 | 67862986 | T | C | 0.022 | -0.284 | 0.045 | 2.81E-10 | 0.0037 | 51.1683 |
| rs17391694 | RNFT1P2 | 1 | 78623626 | T | C | 0.123 | -0.119 | 0.020 | 2.62E-09 | 0.0025 | 34.5074 |
| rs2641348 | ADAM30 | 1 | 120437884 | G | A | 0.108 | -0.121 | 0.020 | 9.65E-10 | 0.0060 | 70.4724 |
| rs2974935 | MTX1 | 1 | 155181843 | T | G | 0.495 | 0.076 | 0.012 | 5.80E-10 | 0.0028 | 37.3938 |
| rs3024505 | IL10 | 1 | 206939904 | A | G | 0.157 | 0.165 | 0.016 | 3.95E-25 | 0.0066 | 95.5698 |
| rs35730213 | C1orf106 | 1 | 200874229 | C | G | 0.281 | -0.151 | 0.014 | 7.84E-28 | 0.0072 | 107.2390 |
| rs36016881 | PARK7 | 1 | 8051241 | G | A | 0.175 | -0.109 | 0.017 | 1.60E-10 | 0.0042 | 60.7362 |
| rs6679677 | PHTF1 | 1 | 114303808 | A | C | 0.098 | -0.185 | 0.022 | 4.67E-17 | 0.0554 | 124.5374 |
| rs6702421 | DENND1B | 1 | 197559324 | T | C | 0.226 | 0.110 | 0.014 | 6.53E-15 | 0.0092 | 119.5755 |
| rs7517847 | IL23R | 1 | 67681669 | G | T | 0.435 | -0.336 | 0.012 | 1.38E-159 | 0.0034 | 40.8987 |
| rs11691685 | TEX41 | 2 | 145481827 | G | A | 0.080 | -0.158 | 0.023 | 1.35E-11 | 0.0045 | 67.4216 |
| rs12694846 | SP140 | 2 | 231148128 | G | A | 0.259 | 0.115 | 0.014 | 2.50E-17 | 0.0071 | 95.2514 |
| rs13001325 | IL1RL1 | 2 | 102939036 | T | C | 0.376 | -0.123 | 0.013 | 1.68E-22 | 0.0037 | 45.7379 |
| rs13407913 | ADCY3 | 2 | 25097644 | G | A | 0.431 | 0.115 | 0.012 | 9.64E-22 | 0.0031 | 41.2884 |
| rs1517352 | STAT4 | 2 | 191931464 | C | A | 0.605 | 0.080 | 0.012 | 1.31E-10 | 0.0030 | 42.0325 |
| rs35320439 | GAL3ST2 | 2 | 242737341 | C | T | 0.310 | 0.084 | 0.014 | 9.89E-10 | 0.0051 | 71.7024 |
| rs6738394 | ARPC2 | 2 | 219110625 | A | G | 0.452 | 0.078 | 0.012 | 8.98E-11 | 0.0069 | 98.6930 |
| rs6738490 | ATG16L1 | 2 | 234161583 | C | T | 0.527 | 0.226 | 0.012 | 4.26E-78 | 0.0255 | 349.9869 |
| rs6740462 | AC074391.1 | 2 | 65667272 | A | C | 0.738 | 0.100 | 0.014 | 1.74E-12 | 0.0064 | 91.7905 |
| rs7608910 | PUS10 | 2 | 61204856 | G | A | 0.391 | 0.121 | 0.012 | 2.95E-23 | 0.0065 | 93.2720 |
| rs77981966 | THADA | 2 | 43777964 | T | C | 0.073 | 0.183 | 0.022 | 2.19E-16 | 0.0030 | 37.3453 |
| rs780094 | GCKR | 2 | 27741237 | C | T | 0.605 | -0.116 | 0.012 | 4.56E-22 | 0.0038 | 49.7615 |
| rs11713774 | SATB1-AS1 | 3 | 18765978 | C | T | 0.143 | 0.133 | 0.017 | 1.09E-14 | 0.0043 | 59.7290 |
| rs3197999 | MST1 | 3 | 49721532 | A | G | 0.281 | 0.155 | 0.013 | 2.05E-33 | 0.0097 | 145.0905 |
| rs34592089 | - | 4 | 102926923 | A | G | 0.053 | 0.199 | 0.025 | 1.41E-15 | 0.0032 | 43.9207 |
| rs6827756 | KIAA1109 | 4 | 123184411 | C | T | 0.625 | -0.079 | 0.013 | 3.27E-10 | 0.0040 | 63.7524 |
| rs7438704 | SLAIN2 | 4 | 48363245 | G | A | 0.644 | 0.084 | 0.013 | 3.42E-11 | 0.0029 | 39.5060 |
| rs11167518 | - | 5 | 150258920 | A | C | 0.081 | 0.280 | 0.020 | 3.21E-45 | 0.0051 | 66.2906 |
| rs1363907 | ERAP2 | 5 | 96252803 | A | G | 0.421 | 0.103 | 0.013 | 3.89E-16 | 0.0037 | 52.1883 |
| rs17388425 | AC008697.1 | 5 | 158824174 | G | C | 0.160 | -0.157 | 0.017 | 6.54E-20 | 0.0292 | 395.535 |
| rs17622378 | C5orf56 | 5 | 131778452 | G | A | 0.419 | 0.190 | 0.012 | 7.17E-56 | 0.0042 | 54.3296 |
| rs181826 | NDFIP1 | 5 | 141526057 | A | C | 0.627 | 0.100 | 0.013 | 4.53E-15 | 0.0031 | 38.1431 |
| rs34804116 | RP11-60A8.1 | 5 | 72539850 | A | C | 0.387 | -0.094 | 0.013 | 1.27E-13 | 0.0117 | 199.143 |
| rs3776414 | DAP | 5 | 10689562 | G | T | 0.376 | 0.089 | 0.012 | 5.04E-13 | 0.0023 | 30.6879 |
| rs4703855 | JMY | 5 | 71693899 | T | C | 0.300 | -0.073 | 0.013 | 3.03E-08 | 0.0176 | 247.9762 |
| rs56163845 | CPEB4 | 5 | 173373948 | G | A | 0.310 | -0.092 | 0.013 | 9.40E-12 | 0.0042 | 54.8953 |
| rs71624119 | ANKRD55 | 5 | 55440730 | A | G | 0.242 | -0.092 | 0.015 | 6.57E-10 | 0.0046 | 61.4532 |
| rs7711427 | RP11-386E5.1 | 5 | 40414886 | C | A | 0.613 | 0.248 | 0.012 | 5.17E-88 | 0.0066 | 83.4496 |
| rs79980175 | TTC33 | 5 | 40521892 | C | A | 0.136 | -0.134 | 0.018 | 1.70E-13 | 0.0036 | 46.4500 |
| rs11152949 | RP11-282C5.1 | 6 | 106449085 | G | A | 0.320 | 0.134 | 0.013 | 2.18E-25 | 0.0104 | 138.1428 |
| rs1267501 | RP11-146I2.1 | 6 | 14715257 | C | T | 0.811 | 0.087 | 0.015 | 9.69E-09 | 0.0030 | 39.0265 |
| rs1847472 | BACH2 | 6 | 90973159 | A | C | 0.342 | -0.085 | 0.013 | 1.09E-10 | 0.0030 | 37.2091 |
| rs212388 | RP1-111C20.3 | 6 | 159490436 | T | C | 0.604 | -0.102 | 0.012 | 1.80E-16 | 0.0033 | 41.6538 |
| rs3129871 | HLA-DRA | 6 | 32406342 | C | A | 0.660 | -0.088 | 0.013 | 1.80E-11 | 0.0076 | 99.3850 |
| rs438475 | NOTCH4 | 6 | 32186245 | A | G | 0.132 | 0.159 | 0.017 | 3.42E-20 | 0.0037 | 47.7641 |
| rs6456426 | ZFP57 | 6 | 21438889 | A | C | 0.498 | -0.099 | 0.012 | 1.37E-16 | 0.0049 | 68.3528 |
| rs6908425 | CDKAL1 | 6 | 20728731 | C | T | 0.784 | 0.104 | 0.015 | 4.81E-12 | 0.0078 | 108.4159 |
| rs7773324 | RP11-157J24.2 | 6 | 382559 | A | G | 0.600 | 0.079 | 0.013 | 1.06E-09 | 0.0058 | 84.7328 |
| rs9264942 | HLA-B | 6 | 31274380 | C | T | 0.353 | 0.151 | 0.013 | 6.78E-32 | 0.0023 | 32.9022 |
| rs9457247 | RP1-167A14.2 | 6 | 167392174 | T | C | 0.540 | 0.124 | 0.012 | 2.08E-23 | 0.0035 | 45.1814 |
| rs9491892 | RP11-394G3.2 | 6 | 128280358 | G | T | 0.150 | 0.138 | 0.016 | 3.80E-17 | 0.0048 | 70.8773 |
| rs9494844 | AL356739.1 | 6 | 137983469 | A | C | 0.252 | -0.089 | 0.014 | 4.18E-10 | 0.0050 | 67.8156 |
| rs11768997 | - | 7 | 50533716 | T | G | 0.135 | 0.226 | 0.021 | 1.83E-27 | 0.0040 | 56.1741 |
| rs1456896 | AC020743.3 | 7 | 50304461 | T | C | 0.689 | 0.098 | 0.013 | 1.03E-13 | 0.0041 | 55.2998 |
| rs2395022 | SMURF1 | 7 | 98750379 | C | A | 0.959 | -0.177 | 0.028 | 3.13E-10 | 0.0119 | 117.8879 |
| rs2538470 | RP5-958B11.2 | 7 | 148220448 | G | A | 0.638 | -0.075 | 0.012 | 1.05E-09 | 0.0025 | 39.5888 |
| rs3801810 | SKAP2 | 7 | 26892531 | A | G | 0.234 | 0.105 | 0.014 | 6.63E-14 | 0.0026 | 37.2374 |
| rs7786444 | JAZF1 | 7 | 28154384 | T | C | 0.116 | 0.112 | 0.018 | 9.83E-10 | 0.0026 | 37.3578 |
| rs10956252 | RP11-136O12.2 | 8 | 126536137 | G | C | 0.619 | 0.119 | 0.012 | 8.34E-22 | 0.0028 | 37.5307 |
| rs6651252 | LINC00824 | 8 | 129567181 | C | T | 0.130 | -0.149 | 0.018 | 3.86E-16 | 0.0050 | 66.3069 |
| rs7015630 | SCb-64M4.1 | 8 | 90875918 | C | T | 0.266 | -0.084 | 0.014 | 9.00E-10 | 0.0066 | 92.0756 |
| rs10758669 | JAK2 | 9 | 4981602 | A | C | 0.650 | -0.150 | 0.012 | 4.19E-34 | 0.0088 | 118.2245 |
| rs11793497 | SNAPC4 | 9 | 139271850 | G | A | 0.423 | 0.169 | 0.012 | 9.80E-44 | 0.0102 | 148.2498 |
| rs7848647 | TNFSF15 | 9 | 117569046 | C | T | 0.675 | 0.141 | 0.013 | 1.55E-27 | 0.0139 | 192.3405 |
| rs10995271 | ZNF365 | 10 | 64438486 | C | G | 0.610 | -0.188 | 0.012 | 4.92E-53 | 0.0082 | 117.8204 |
| rs11185982 | SH2D4B | 10 | 82305627 | C | T | 0.152 | -0.109 | 0.017 | 1.05E-10 | 0.0087 | 111.0199 |
| rs1250573 | ZMIZ1 | 10 | 81042475 | A | G | 0.316 | -0.142 | 0.013 | 5.86E-26 | 0.0152 | 209.7891 |
| rs2153283 | IPMK | 10 | 59972299 | A | C | 0.217 | -0.109 | 0.016 | 2.39E-12 | 0.0036 | 53.0866 |
| rs2227551 | PLAU | 10 | 75669190 | T | G | 0.729 | 0.099 | 0.014 | 4.72E-13 | 0.0028 | 37.6707 |
| rs303429 | MAP3K8 | 10 | 30708441 | T | C | 0.600 | 0.076 | 0.012 | 8.38E-10 | 0.0031 | 41.7187 |
| rs34779708 | CREM | 10 | 35466185 | G | T | 0.351 | 0.134 | 0.012 | 1.90E-27 | 0.0040 | 49.1310 |
| rs61839660 | IL2RA | 10 | 6094697 | T | C | 0.090 | 0.148 | 0.020 | 3.19E-13 | 0.0168 | 234.9667 |
| rs7085798 | NKX2-3 | 10 | 101288347 | A | C | 0.518 | -0.174 | 0.012 | 1.53E-47 | 0.0039 | 52.3161 |
| rs11236797 | RP11-672A2.7 | 11 | 76299649 | A | C | 0.444 | 0.181 | 0.012 | 8.54E-51 | 0.0280 | 225.7827 |
| rs12796489 | CARS | 11 | 3059360 | A | C | 0.023 | -0.792 | 0.053 | 4.96E-51 | 0.0030 | 39.2411 |
| rs34787213 | CD6 | 11 | 60799046 | T | C | 0.140 | -0.150 | 0.018 | 2.85E-16 | 0.0161 | 224.699 |
| rs559928 | RPS6KA4 | 11 | 64150370 | C | T | 0.813 | 0.099 | 0.016 | 3.75E-10 | 0.0054 | 66.9014 |
| rs10878302 | LRRK2 | 12 | 40669826 | A | T | 0.929 | 0.157 | 0.024 | 4.20E-11 | 0.0023 | 31.7988 |
| rs28999107 | LTBR | 12 | 6493100 | T | G | 0.439 | 0.086 | 0.013 | 1.29E-11 | 0.0027 | 37.2555 |
| rs3184504 | SH2B3 | 12 | 111884608 | C | T | 0.507 | -0.068 | 0.012 | 1.71E-08 | 0.0033 | 43.5161 |
| rs76906269 | LRRK2 | 12 | 40607709 | G | A | 0.019 | 0.394 | 0.037 | 1.75E-26 | 0.0036 | 45.8249 |
| rs7969592 | IFNG-AS1 | 12 | 68579649 | G | A | 0.475 | -0.073 | 0.012 | 1.04E-09 | 0.0057 | 113.419 |
| rs1927681 | RP11-545M8.4 | 13 | 27558881 | A | T | 0.443 | 0.089 | 0.012 | 2.42E-13 | 0.0031 | 42.1883 |
| rs6561151 | LINC00284 | 13 | 44484706 | A | G | 0.224 | 0.147 | 0.014 | 4.68E-25 | 0.0027 | 36.6894 |
| rs915286 | LINC00598 | 13 | 40695992 | A | G | 0.549 | 0.067 | 0.012 | 2.59E-08 | 0.0039 | 53.6266 |
| rs9554587 | UBAC2 | 13 | 100040654 | G | A | 0.224 | -0.095 | 0.015 | 8.29E-11 | 0.0022 | 30.9943 |
| rs9594766 | RP11-413N19.2 | 13 | 43040043 | A | G | 0.529 | -0.074 | 0.012 | 1.39E-09 | 0.0075 | 106.9028 |
| rs11159833 | GPR65 | 14 | 88476004 | T | C | 0.087 | 0.155 | 0.021 | 7.59E-14 | 0.0038 | 55.9082 |
| rs1569328 | FOS | 14 | 75741751 | T | C | 0.170 | -0.109 | 0.017 | 6.47E-11 | 0.0034 | 42.6716 |
| rs17293632 | SMAD3 | 15 | 67442596 | T | C | 0.236 | 0.128 | 0.014 | 3.70E-20 | 0.0034 | 47.5795 |
| rs72727394 | RASGRP1 | 15 | 38847022 | T | C | 0.201 | 0.103 | 0.015 | 5.28E-12 | 0.0060 | 84.5743 |
| rs11117431 | RP11-542M13.2 | 16 | 86015316 | G | A | 0.198 | -0.149 | 0.016 | 1.09E-19 | 0.0074 | 99.2933 |
| rs1646019 | RMI2 | 16 | 11359680 | T | C | 0.303 | -0.111 | 0.013 | 8.62E-17 | 0.0052 | 69.2627 |
| rs2270395 | LINC02168 | 16 | 50846832 | T | C | 0.762 | 0.124 | 0.014 | 8.93E-18 | 0.0056 | 73.7369 |
| rs26528 | IL27 | 16 | 28517709 | C | T | 0.458 | 0.120 | 0.012 | 1.29E-22 | 0.0071 | 82.4366 |
| rs6500315 | RP11-21B23.3 | 16 | 50508101 | G | A | 0.775 | 0.146 | 0.015 | 2.18E-23 | 0.0253 | 347.5901 |
| rs7194886 | NOD2 | 16 | 50725193 | T | C | 0.436 | -0.227 | 0.012 | 1.42E-77 | 0.0071 | 95.7654 |
| rs1292053 | TUBD1 | 17 | 57963537 | G | A | 0.442 | 0.091 | 0.012 | 1.75E-14 | 0.0081 | 107.8122 |
| rs12949918 | STAT3 | 17 | 40526273 | C | T | 0.419 | -0.104 | 0.012 | 3.47E-17 | 0.0087 | 120.9925 |
| rs3853824 | C17orf67 | 17 | 54880993 | C | T | 0.639 | 0.081 | 0.013 | 1.17E-10 | 0.0053 | 71.0556 |
| rs4795397 | IKZF3 | 17 | 38023745 | G | A | 0.471 | 0.132 | 0.012 | 3.84E-28 | 0.0041 | 58.7980 |
| rs9889296 | AC005549.3 | 17 | 32570547 | A | G | 0.272 | -0.143 | 0.014 | 2.96E-25 | 0.0031 | 41.5093 |
| rs2847293 | PTPN2 | 18 | 12782448 | T | A | 0.840 | -0.167 | 0.016 | 6.14E-26 | 0.0075 | 110.9284 |
| rs7236492 | NFATC1 | 18 | 77220616 | T | C | 0.154 | -0.100 | 0.017 | 9.09E-09 | 0.0026 | 33.0258 |
| rs17694108 | SLC7A10 | 19 | 33731551 | A | G | 0.280 | 0.080 | 0.013 | 3.29E-09 | 0.0067 | 84.8688 |
| rs2024092 | SBNO2 | 19 | 1124031 | A | G | 0.216 | 0.148 | 0.014 | 7.13E-25 | 0.0026 | 35.0041 |
| rs35164067 | CDC37 | 19 | 10525181 | A | G | 0.204 | -0.143 | 0.016 | 3.19E-20 | 0.0027 | 36.8028 |
| rs516246 | FUT2 | 19 | 49206172 | T | C | 0.465 | 0.115 | 0.012 | 1.33E-20 | 0.0074 | 106.068 |
| rs640466 | LSM14A | 19 | 34670725 | C | T | 0.374 | -0.076 | 0.012 | 1.31E-09 | 0.0065 | 86.6010 |
| rs259964 | ZNF831 | 20 | 57824309 | G | A | 0.541 | -0.071 | 0.012 | 2.08E-09 | 0.0213 | 242.8066 |
| rs6062496 | TNFRSF6B | 20 | 62329099 | A | G | 0.569 | 0.120 | 0.012 | 3.82E-22 | 0.0035 | 48.8980 |
| rs6074022 | LINC01754 | 20 | 44740196 | T | C | 0.750 | -0.096 | 0.014 | 2.70E-12 | 0.0025 | 35.8951 |
| rs6111031 | SIRPB3P | 20 | 1682037 | T | C | 0.159 | -0.282 | 0.018 | 9.61E-55 | 0.0071 | 93.6247 |
| rs1297258 | AJ006998.2 | 21 | 16806709 | T | C | 0.425 | -0.127 | 0.012 | 2.11E-25 | 0.0079 | 108.4841 |
| rs2284553 | IFNGR2 | 21 | 34776695 | G | A | 0.590 | 0.103 | 0.012 | 5.63E-17 | 0.0052 | 70.1034 |
| rs8127691 | - | 21 | 45614860 | C | T | 0.613 | -0.123 | 0.012 | 4.48E-24 | 0.0072 | 102.4266 |
| rs140143 | - | 22 | 30173109 | T | G | 0.390 | -0.127 | 0.014 | 7.95E-21 | 0.0122 | 156.1843 |
| rs2413583 | AL031590.1 | 22 | 39659773 | T | C | 0.165 | -0.210 | 0.017 | 7.72E-36 | 0.0077 | 87.6161 |
| rs727563 | ACO2 | 22 | 41867377 | T | C | 0.797 | -0.092 | 0.014 | 1.88E-10 | 0.0027 | 40.5845 |
